# The deubiquitinase USP5 prevents accumulation of protein aggregates in cardiomyocytes

**DOI:** 10.1126/sciadv.ado3852

**Published:** 2025-01-22

**Authors:** Yvonne Eibach, Silke Kreher, Mareike S. Poetsch, Ay Lin Kho, Ulrich Gaertner, Christoph S. Clemen, Rolf Schröder, Kai Guo, Hendrik Milting, Benjamin Meder, Michael Potente, Manfred Richter, Andre Schneider, Silke Meiners, Mathias Gautel, Thomas Braun

**Affiliations:** ^1^ Department of Cardiac Development and Remodeling, Max Planck Institute for Heart and Lung Research, Bad Nauheim, Germany.; ^2^ German Center for Cardiovascular Research (DZHK), Berlin, Germany.; ^3^ Member of the Cardio-Pulmonary Institute (CPI), Bad Nauheim, Frankfurt, Giessen, Germany.; ^4^ Randall Centre for Cell and Molecular Biophysics, King’s College London, BHF Centre of Excellence, London, UK.; ^5^ University of Giessen, Institute of Anatomy and Cell Biology, Giessen, Germany.; ^6^ Institute of Aerospace Medicine, German Aerospace Center (DLR), Cologne, Germany.; ^7^ Institute of Vegetative Physiology, Medical Faculty, University of Cologne, Cologne, Germany.; ^8^ Institute for Neuropathology, University Hospital Erlangen, Friedrich-Alexander University Erlangen-Nürnberg, Erlangen, Germany.; ^9^ Research Center Borstel/Leibniz Lung Center, Airway Research Center North (ARCN), Member of the German Center for Lung Research (DZL), 23845 Borstel, Germany.; ^10^ Institute of Experimental Medicine, Christian-Albrechts University, Kiel, Germany.; ^11^ Erich and Hanna Klessmann Institute for Cardiovascular Research and Development, Clinic for Thoracic and Cardiovascular Surgery, Heart and Diabetes Center NRW, Bad Oeynhausen, Germany.; ^12^ Department of Medicine III, University of Heidelberg, INF 410, 69120 Heidelberg, Germany.; ^13^ Berlin Institute of Health (BIH) and Charité–Universitätsmedizin Berlin, corporate member of Freie Universität Berlin, Humboldt-Universität zu Berlin, Berlin, Germany.; ^14^ Max Delbrück Center for Molecular Medicine (MDC), Berlin, Germany.; ^15^ Department of Cardiac Surgery, Kerckhoff-Clinic, Bad Nauheim, Germany.

## Abstract

Protein homeostasis is crucial for maintaining cardiomyocyte (CM) function. Disruption of proteostasis results in accumulation of protein aggregates causing cardiac pathologies such as hypertrophy, dilated cardiomyopathy (DCM), and heart failure. Here, we identify ubiquitin-specific peptidase 5 (USP5) as a critical determinant of protein quality control (PQC) in CM. CM-specific loss of *mUsp5* leads to the accumulation of polyubiquitin chains and protein aggregates, cardiac remodeling, and eventually DCM. USP5 interacts with key components of the proteostasis machinery, including PSMD14, and the absence of USP5 increases activity of the ubiquitin-proteasome system and autophagic flux in CMs. Cardiac-specific *hUSP5* overexpression reduces pathological remodeling in pressure-overloaded mouse hearts and attenuates protein aggregate formation in titinopathy and desminopathy models. Since CMs from humans with end-stage DCM show lower USP5 levels and display accumulation of ubiquitinated protein aggregates, we hypothesize that therapeutically increased USP5 activity may reduce protein aggregates during DCM. Our findings demonstrate that USP5 is essential for ubiquitin turnover and proteostasis in mature CMs.

## Teaser

The deubiquitinase USP5 prevents accumulation of polyubiquitin chains and protein aggregates in cardiomyocytes.

## INTRODUCTION

Cellular functions rely on a sophisticated network of molecular chaperones and other factors, assisting in the acquisition of proper protein conformations (*1*). Gene mutations, but also cellular stress conditions, can result in the formation of misfolded proteins that cannot be rescued by chaperones but need to be eliminated by degradation (*2*). This task is mainly accomplished by the ubiquitin-proteasome system (UPS) and to a lesser extent by autophagy, although the ratio between UPS and autophagy differs depending on cellular states (*3*). Disease conditions, such as the presence of damaged proteins due to mutations or degenerative aging processes, can overload the protein degradation machinery, resulting in the accumulation of mutated, critically misfolded, or damaged proteins, which compromise cellular functions or cause cellular death (*2*). Cardiomyocytes (CMs) are particularly susceptible to disturbed protein quality control (PQC) due to high basal metabolic and mechanical stress (*2*). The necessity for highly efficient PQC in CMs is apparent in pressure-overloaded hearts, where increased proteotoxic stress induces formation of intracellular protein aggregates, triggering activation of proteasomal and autophagic clearance pathways (*4*). Likewise, mutations in genes coding for cytoskeletal, nuclear envelope, and sarcomere proteins often give rise to protein aggregates exerting proteotoxicity (*5*), which eventually cause cardiomyopathy, for example, dilated cardiomyopathy (DCM) (*6*–*8*).

The ubiquitin-dependent protein degradation machinery, including the proteasome and autophagy, has been intensively investigated (*9*). The 26*S* proteasome is mainly composed of the 20*S* proteolytic core particle and the 19*S* regulatory particle (*10*), which recognize and deubiquitinate K48-linked ubiquitin-conjugated proteins, thereby releasing unanchored polyubiquitin chains that are further processed by deubiquitinating enzymes (DUBs) (*11*, *12*). Accumulation of unanchored polyubiquitin chains causes dysfunction of the UPS, resulting in detrimental consequences for cells including CMs (*13*). Autophagy degrades larger protein aggregates and damaged organelles and is mainly facilitated by K63-linked polyubiquitin conjugates (*14*). However, fundamental questions about the cellular function of DUBs in mammals, their role in specific cell types, and disease processes remain unanswered. Given the important role of DUBs in ubiquitin metabolism and the paramount function of the UPS in various heart diseases, we assumed that DUBs might exert a crucial function in cardiac PQC (*15*).

DUBs are a class of (mostly) cysteine proteases that play important roles as regulators of the ubiquitin system (*16*). Several studies demonstrated that DUBs process inactive ubiquitin precursors, proofread ubiquitin-protein conjugates, remove ubiquitin adducts from cellular proteins, and keep the 26*S* proteasome free of inhibitory polyubiquitin chains (*17*, *18*). For example, in vitro assays indicated that the highly conserved proteasomal DUB ubiquitin-specific peptidase 5 (USP5; also known as isopeptidase T) disassembles the majority of free, protein unanchored K48-, K63-, K29/K6-linked and linear polyubiquitin chains derived from PSMD14-mediated deubiquitination (*11*, *19*–*21*). USP5 disassembles polyubiquitin by sequential hydrolysis from the proximal end of the chain to yield monoubiquitin (*11*, *19*, *20*). USP5 has been claimed to act as a recycling DUB, required to maintain cellular ubiquitin homeostasis (*22*), which was later confirmed by inactivation of *Usp5* homologs in *Saccharomyces cerevisiae* (*23*) and *Dictyostelium discoideum* (*24*), and by short hairpin RNA (shRNA)–mediated knockdown of *Usp5* in ARN8 melanoma cells (*25*). Recently, it was reported that USP5 interacts with Atg1/ULK1 and regulates autophagy (*26*). Although USP5 has been implicated in several pathological conditions, including tumor formation, pathological pain, developmental abnormalities, inflammatory diseases, and viral infections (*27*), a potential role in heart diseases has not yet been explored.

Here, we describe a crucial function of USP5 for maintaining cardiac proteostasis. We demonstrate that CM-specific inactivation of *Usp5* causes DCM and lethal heart failure in mice. We observed a profound imbalance of the cellular ubiquitin pool, which results in the accumulation of ubiquitinated protein aggregates. In addition, we found that CMs of end-stage DCM patients (ejection fraction <30%) show reduced levels of USP5 protein, which is accompanied with accumulation of ubiquitinated protein aggregates. Overexpression of *Usp5* in CMs exerted beneficial effects in pressure-overloaded hearts and attenuated protein aggregate formation in models of desmino- and titinopathies.

## RESULTS

### Components of the cellular PQC machinery including PSMD14 interact with USP5

The efficient function of protein catabolism depends on ubiquitin conjugate disassembly, which is essential for the recycling of ubiquitin. UPS-dependent removal of polyubiquitin chains from proteins before proteasomal degradation is mainly catalyzed by the 19*S* proteasomal DUB PSMD14 (also known as RPN11/POH1). We wondered whether additional DUBs collaborate with PSMD14 to regulate ubiquitin turnover and reutilization. Hence, we screened for potential interaction partners of PSMD14 by coimmunoprecipitation, using a panel of 40 different ubiquitin-specific peptidases (USPs). We found that 11 of the 40 USPs coimmunoprecipitated with PSMD14, including USP5 (fig. S1, A to C). This observation drew our attention, since USP5 was reported to regulate PQC (*23*–*28*).

**Fig. 1.**
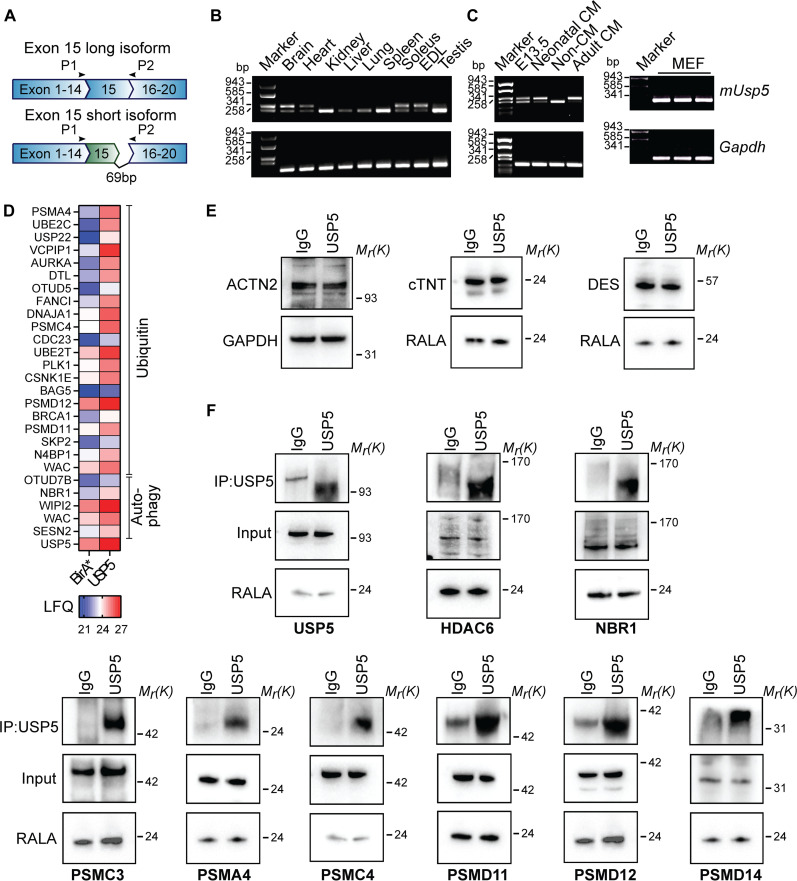
Differentially spliced isoforms of USP5 interact with parts of the PQC machinery. (**A**) Scheme depicting alternative spliced transcripts of *Usp5*. The short isoform of *Usp5* lacks 69 base pairs (bp) of exon 15. Location of primers (P1 and P2) used for semiquantitative RT-PCR are indicated by arrows. (**B**) RT-PCR showing spliced variants of *mUsp5* in diverse, adult mouse tissues. (**C**) RT-PCR showing spliced variants of *mUsp5* in CMs at different developmental stages (E13.5: embryonic day 13.5, neonatal and adult), adult non-CMs, and MEFs. [(B) and (C)] *Gapdh* was used as loading control. (**D**) Heatmap showing concentrations of biotinylated proteins identified in BirA*- and USP5-BirA*–transduced cells (BioID screen). Only proteins related to ubiquitin- and autophagy-related pathways are shown. Concentrations were determined by label-free quantification (LFQ). (**E**) Western blot analysis of cardiac markers ACTN2, cTNT, and desmin in HL-1 CMs. (**F**) Western blot analysis of input and coimmunoprecipitation (coIP) samples to verify selected interaction partners of endogenous USP5 in HL-1 CMs. [(E) and (F)] GAPDH or RALA was used as loading control for the input. Multiple antibodies were used to probe a single blot, allowing the repeated use of a single loading control as a reference for different reactions.

*Usp5* undergoes PTBP1-dependent differential splicing, resulting in the formation of a long and a short isoform (*29*). To identify the *Usp5* isoforms that are present in different murine tissues and particularly in the heart, we performed reverse transcription polymerase chain reaction (RT-PCR) analysis using primers specific for mouse *Usp5* exon 15 (Fig. 1A). The long isoform of *Usp5* was present in adult organs with high numbers of postmitotic cells, e.g., the heart, skeletal muscles, and the brain, whereas the short isoform showed a broader expression pattern (Fig. 1B). Non-CMs in adult hearts, adult hepatocytes, and mouse embryonic fibroblasts (MEFs) exclusively produced the short isoform (Fig. 1C and fig. S5A). In contrast to expression of the long *Usp5* isoform in mature adult CMs, both isoforms were expressed in proliferating embryonic and neonatal CMs (Fig. 1C).

To characterize the role of USP5 in cellular PQC, we determined its interactome using a BioID approach (*30*) by transfecting USP5-BirA* fusion proteins into human embryonic kidney (HEK) 293 cells. The promiscuous BirA* biotin ligase biotinylates proteins proximate to USP5 in living cells and thereby complements coimmunoprecipitation techniques. Mass spectrometry (MS) analysis identified different ubiquitin ligases, DUBs, as well as elements of the 26*S* proteasome and the autophagic machinery as putative interaction partners of USP5 (Fig. 1D; fig. S2, A to D; and Table S1). We observed enrichment of proteins regulating protein catabolic processes (fig. S2E), while most of the detected proteins was associated with ubiquitin ligases or the proteasome complex (fig. S2F). To validate the results, selected putative interaction partners were analyzed by coimmunoprecipitation with endogenous USP5 in HL-1 CMs (Fig. 1, E and F) (*31*). The BioID approach did not detect PSMD14, although PSMD14 robustly coimmunoprecipitated with USP5 in HEK293 (fig. S2G) and cardiac HL-1 cells (Fig. 1F). Both USP5 isoforms interacted with the protein deacetylase HDAC6 (fig. S2H and Fig. 1F), which promotes autophagic clearance of protein aggregates in cells with impaired proteasomal capacity and binds to ubiquitin chains released by PSMD14 (*32*, *33*). We reason that the failure of the BioID approach to detect an interaction between USP5 and PSMD14 might be due to the specific conditions in HEK293 cells or technical issues with the biotinylation approach.

### USP5 prevents ubiquitin-proteasome and cardiac stress responses in CMs

**Fig. 2.**
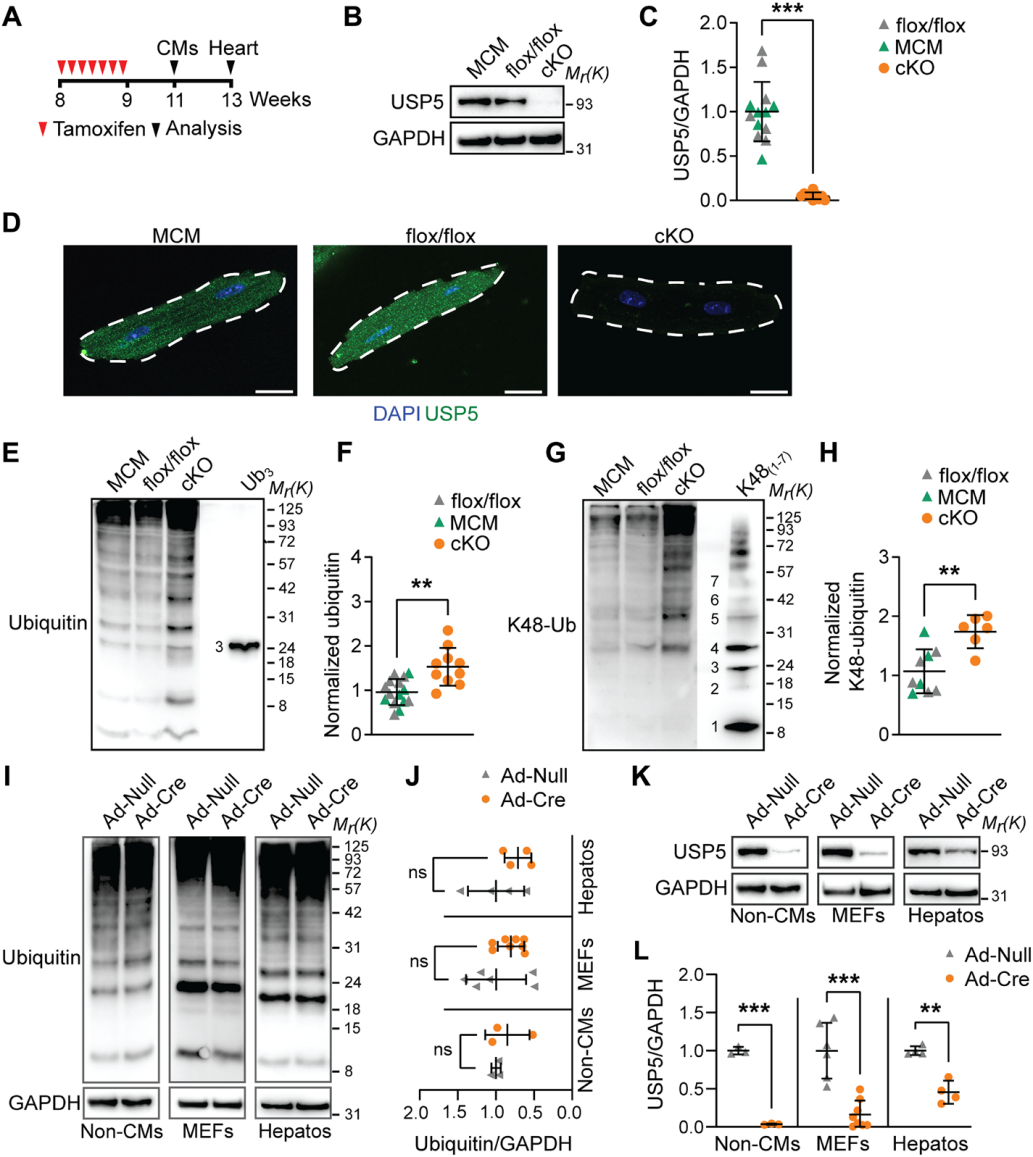
Inactivation of *Usp5* increases polyubiquitin levels in CMs but not in MEFs and hepatocytes. (**A**) Scheme for CM-restricted inactivation of *Usp5*. Eight-week-old flox/flox, MCM, and cKO mice were intraperitoneally injected for 7 days with tamoxifen (100 mg kg^−1^). CMs were isolated and analyzed 15 days after the last tamoxifen administration (= 11 weeks of age), while hearts were isolated and analyzed 24 days after the last tamoxifen administration (=13 weeks of age). (**B** and **C**) Immunoblotting (B) of USP5 in CMs from flox/flox, MCM, and cKO hearts and quantification (C), flox/flox (*n* = 7), MCM (*n* = 6), and cKO (*n* = 10). Mann-Whitney test. (**D**) Staining for USP5 (green) and nuclei (DAPI, blue) in CMs from flox/flox, MCM, and cKO mice. Scale bar, 20 μm. (**E** and **F**) Immunoblotting (E) and quantification (F) for ubiquitin in flox/flox (*n* = 6), MCM (*n* = 9), and cKO (*n* = 10) CMs with comigration of purified tri-ubiquitin (Ub_3_, 25 ng). (**G** and **H**) Immunoblotting (G) and quantification (H) for K48-linkage–specific ubiquitin chains in flox/flox (*n* = 4), MCM (*n* = 5), and cKO (*n* = 6) CMs with comigration of purified K48-linkage–specific polyubiquitin chains 1 to 7 [K48_(1–7)_, 25 ng]. [(F) and (H)] Welch’s unequal variances *t* test. (**I** and **J**) Immunoblotting (I) and quantification (J) for ubiquitin in primary non-CM, MEFs, and hepatocytes (Hepatos) after Ad-Null or Ad-Cre transduction (*n* ≥ 3). (**K** and **L**) Immunoblotting (K) and quantification (L) for USP5 non-CMs, MEFs, and hepatocytes after Ad-Null or Ad-Cre transduction (*n* ≥ 3). [(I) and (K)] After probing non-CMs and hepatocytes for ubiquitin and the GAPDH loading control, the same membrane was reprobed with anti-USP5 antibodies. [(J) and (L)] Two-way ANOVA with Sidak’s multiple comparison test. [(E) and (G)] Ub_3_ and K48_(1–7)_ controls were exposed shorter than the samples. Ubiquitin levels normalized to total protein. [(B), (I), and (K)] GAPDH was used as a loading control. **P ≤ 0.01; ***P ≤ 0.001.

To address the physiological role of USP5 in the heart, we generated *Usp5flox/flox* mice, which were crossed to *Myh6-MerCreMer* mice, allowing specific deletion of the *Usp5* gene in mature CMs. Treatment of the resulting *Myh6-MerCreMer//Usp5flox/flox* line (hereafter called cKO) with tamoxifen for seven consecutive days (Fig. 2A) completely abolished *Usp5* expression (fig. S3A) and USP5 protein levels in cKO CMs, but had no effects in control *Myh6-MerCreMer* (hereafter called MCM) and *Usp5flox/flox* (flox/flox) mice (Fig. 2, B to D).

Transcriptional profiling of *Usp5*-deficient CMs revealed expression differences of 229 genes (fig. S3B; Table S2), mainly in Gene Ontology (GO) terms related to structural components of the cytoskeleton and proteasome (fig. S3C). In addition to multiple PQC-linked genes, stress-response genes linked to cardiac remodeling, such as *Nppa* and *Myh7* (fig. S3D), and the ubiquitin precursors *Ubb* and *Ubc* (fig. S3E) were elevated. RT–quantitative PCR (RT-qPCR) analysis verified changes in expression of selected genes (fig. S3F).

Shotgun MS analysis of CMs lacking *Usp5* identified 186 differentially present proteins (fig. S4A and Table S3), primarily belonging to the ubiquitin ligase or proteasome complex (fig. S4, B to D). Immunoblot analysis verified increased levels of multiple 19*S* and 20*S* proteasome subunits (fig. S4, E to H). Together, the data indicate that inactivation of *Usp5* in adult CMs causes higher ubiquitin and cardiac stress responses, as well as alterations of the proteasome system.

### USP5 is indispensable to maintain PQC in adult CMs

In line with up-regulated *Ubb* and *Ubc* expression levels (fig. S3E), deletion of *Usp5* increased total ubiquitin concentrations in CMs, mainly due to elevated polyubiquitin levels (Fig. 2, E and F). Furthermore, we detected an increase of K48-linked ubiquitin chains, foremost at high molecular weights (Fig. 2, G and H). In contrast, no consistent changes in ubiquitin levels were found in *Usp5*-deficient non-CMs from the adult myocardium, MEFs, and adult hepatocytes (Fig. 2, I and J), which were obtained by transduction of a *Cre*-encoding adenovirus (Ad-Cre) into primary cells of *Usp5flox/flox* mice (Fig. 2, K and L). These data indicate that USP5 is crucial to maintain cellular ubiquitin conjugate levels in CMs but to a lesser extent in non-CMs from the heart, MEFs, or hepatocytes.

**Fig. 3.**
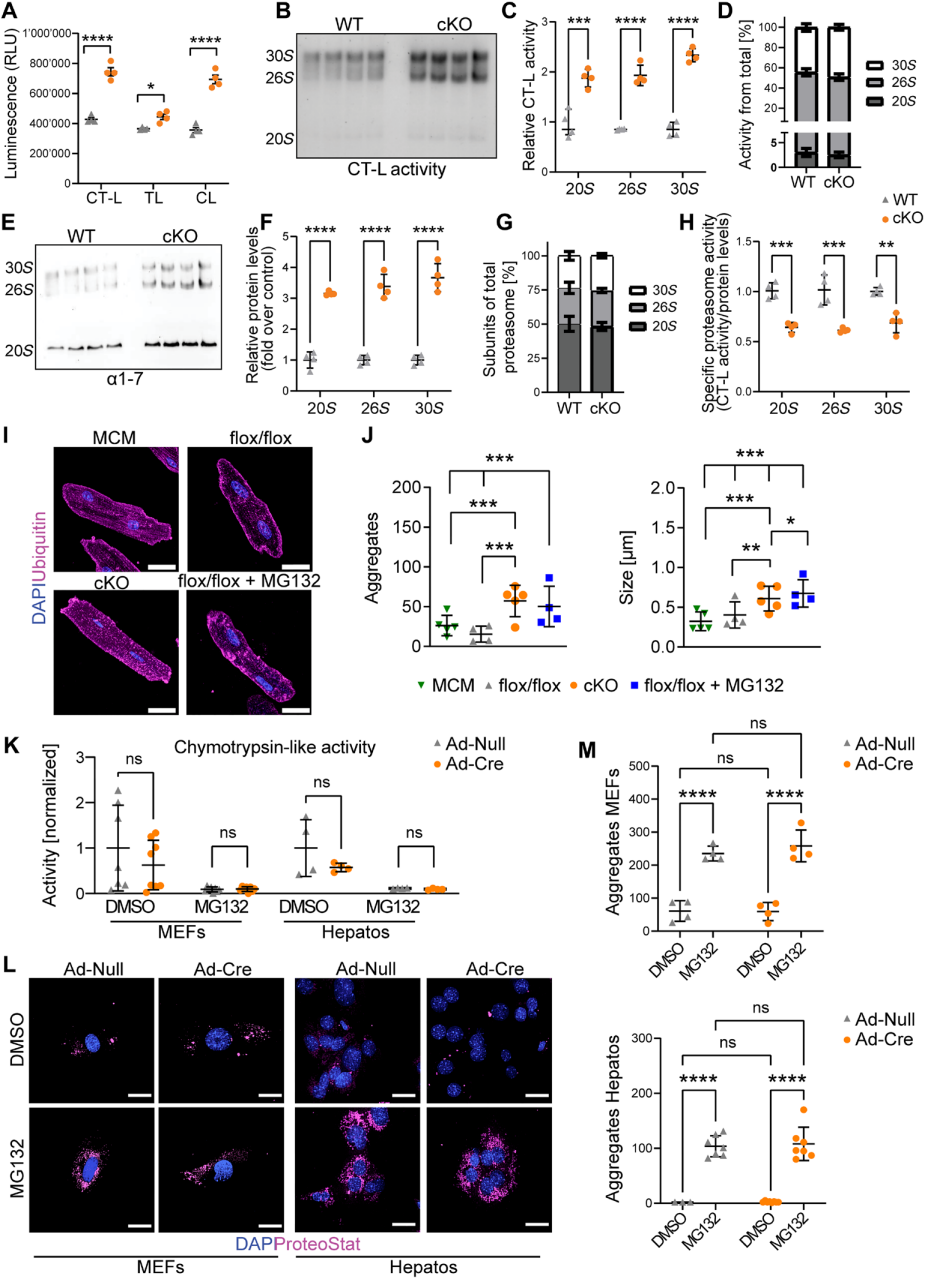
Deletion of *Usp5* results in imbalanced proteasomal activity and accumulation of protein aggregates in CMs but not in MEFs or hepatocytes. (**A**) Chymotrypsin-like (CT-L), trypsin-like (T-L), and caspase-like (C-L) activities in extracts of WT and cKO isolated CMs (*n* = 4), using catalytic site–specific substrates (GLO assay). (**B** and **E**) Native gel analysis of the same samples. (B) In-gel CT-L proteasome activity of *Usp5*-deficient and WT CMs (*n* = 4). (**C** and **D**) Quantification of CT-L activity (B) of proteasome complexes [30*S*, 26*S*, and 20*S*; (C)] and ratios (D). (E) Abundance of distinct proteasome complexes, determined by blotting of the native gel for α1 to α7 proteasome subunits in WT and cKO isolated CMs (*n* = 4). (**F** and **G**) Quantification of 30*S*, 26*S*, and 20*S* abundances in (F) and relative distribution (G) in WT and cKO CMs. (**H**) Specific activity of proteasome complexes (activity/abundance). (**I**) Staining for ubiquitin (magenta) and nuclei (DAPI, blue) in CMs of different groups as indicated. Scale bar, 50 μm. (**J**) Number and size of protein aggregates in flox/flox (*n* = 4), MCM (*n* = 5), and cKO (*n* = 5) CMs and flox/flox CMs treated with MG132 (*n* = 4). Two-way ANOVA with Dunn’s multiple comparison test. [(A) to (J)] WT mice received no tamoxifen treatment. (**K**) CT-L activity in MEFs and hepatocytes after Ad-Null or Ad-Cre transduction and with or without MG132 treatment. Ad-Null: MEFs *n* = 7, hepatocytes *n* = 4; Ad-Cre: MEFs *n* = 8, hepatocytes *n* = 4. [(A), (C), (F), (H), and (K)] Two-way ANOVA with Sidak’s multiple comparison test. (**L**) ProteoStat staining (magenta) and nuclei (DAPI, blue) in MEFs and hepatocytes after Ad-Null or Ad-Cre transduction, treated with or without MG132. Scale bar, 25 μm. (**M**) Number of protein aggregates in MEFs (*n* = 4) and hepatocytes (*n* = 3 to 8) after Ad-Null or Ad-Cre transduction and treatment with or without MG132. (M) Two-way ANOVA with Tukey’s multiple comparison test. *P ≤ 0.05; **P ≤ 0.01; ***P ≤ 0.001, ****P ≤ 0.0001.

The MS-based proteome analysis revealed an up-regulation of proteasome subunits after deletion of *Usp5* (fig. S4, B to H), which prompted us to evaluate proteasome proteolytic activity in isolated CMs. Inactivation of *Usp5* increased the total chymotrypsin-like (CT-L), trypsin-like (T-L), and caspase-like (C-L) peptidase activities (Fig. 3A). To further quantify proteasome function and distinguish proteasome complexes, we performed in-gel proteasome assays (*34*), which revealed a twofold increase in the CT-L activity of 30*S*, 26*S*, and 20*S* proteasome complexes in *Usp5*-deficient CMs compared to wild-type (WT) controls (Fig. 3, B and C). The relative contribution of the 30*S*, 26*S*, and 20*S* proteasome complexes to total activity was not substantially altered by the loss of USP5 (Fig. 3D). The elevated proteasome activity was accompanied by a threefold increase in the assembly of all three proteasome complexes as determined by blotting of the same gel for 20*S* α subunits (Fig. 3, E and F), without any changes in the relative distribution (Fig. 3G). However, when assessing the specific activity of the proteasome—calculated by dividing the relative CT-L activity by the relative abundance of the complexes—we observed an approximate 30% reduction in cKO CMs (Fig. 3H). We concluded that the elevated levels of (poly)ubiquitinated proteins lead to dysregulated UPS-dependent proteolysis in *Usp5*-deficient CMs. In accordance, immunofluorescence staining revealed an increase in the abundance and size of ubiquitin-containing protein aggregates in CMs lacking *Usp5* (Fig. 3, I and J). Here, treatment with MG132 further augmented the size of aggregates in the absence of USP5 (Fig. 3, I and J), but had no effects on cardiac *Usp5* expression (fig. S3A).

In contrast to CMs, inactivation of *Usp5* did not change the CT-L activity of the proteasome in MEFs and hepatocytes (Fig. 3K). Likewise, knockout (KO) of *Usp5* did not raise the number of ProteoStat reagent–stained protein aggregates in MEFs or hepatocytes, which were readily induced by additional treatment with MG132 (Fig. 3, L and M). Overall, we conclude that *Usp5* deletion impairs PQC in postmitotic CMs, but not in MEFs or hepatocytes, leading to the accumulation of protein aggregates. To further explore the function of USP5 in proliferating cells, we used HL-1 CMs (*31*), which have been used before to study PQC, protein aggregation, and autophagy (*35*–*39*). In contrast to adult postmitotic CMs, HL-1 CMs express the short isoform of *Usp5* (fig. S5A). Efficient knockdown of *Usp5* by small interfering RNAs (siRNAs) was verified by immunofluorescence (fig. S5B) and immunoblotting (fig. S5, C and D). Knockdown of *Usp5* did not affect the LC3I/II ratio or p62 protein levels, neither with nor without MG132 treatment (fig. S5, C and D). Likewise, protein aggregate formation did not change in HL-1 CMs (fig. S5, E and F) and ubiquitin levels were not altered (fig. S5G). The observation that knockdown of USP5 does not affect PQC in proliferating HL-1 CMs provides further support for the hypothesis that USP5 is more relevant for postmitotic than for proliferating CMs.

### *Usp5* deletion enhances autophagic flux in CMs

**Fig. 4.**
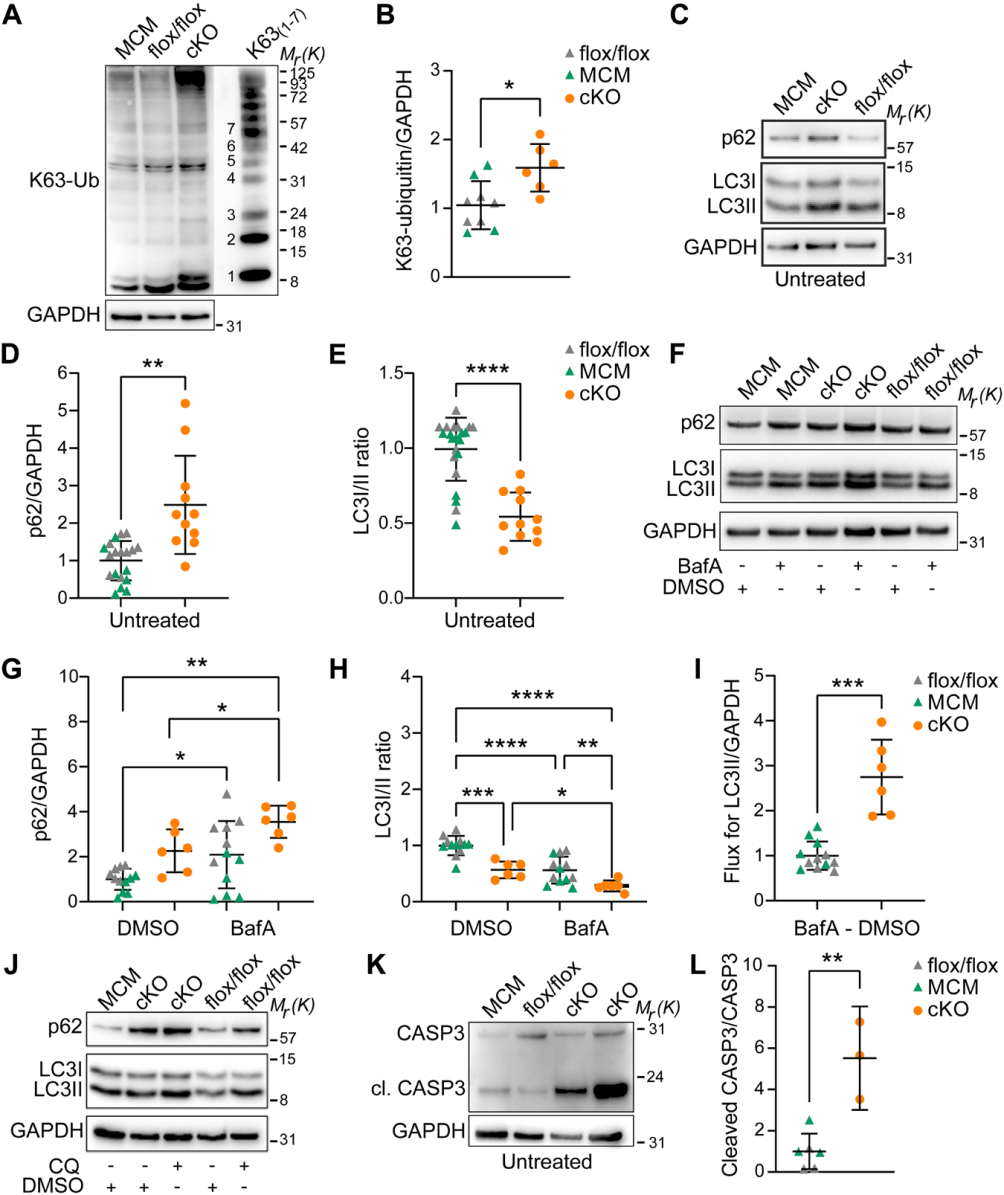
Loss of *Usp5* increases autophagic flux in CMs. (**A**) Immunoblotting for K63-linkage–specific ubiquitin chains in MCM, flox/flox, and cKO CMs with comigration of purified K63-linkage–specific polyubiquitin chains 1 to 7 [K63_(1–7)_, 25 ng, separated by a single lane, shown at a lower exposure time compared to the samples]. (**B**) Levels of K63-polyubiquitin chains normalized to total protein in flox/flox (*n* = 5), MCM (*n* = 4), and cKO (*n* = 6) CMs. (**C**) Immunoblotting for p62 and LC3I/II in CMs with the indicated genotypes without treatment. (**D**) p62 protein levels normalized to GAPDH in flox/flox (*n* = 11), MCM (*n* = 8), and cKO (*n* = 11) CMs. (**E**) Quantification of the LC3I/II ratio in CMs with the indicated genotypes (*n* = 11). Mann-Whitney test. (**F**) Immunoblotting for p62 and LC3I/II in flox/flox, MCM, and cKO CMs treated with either DMSO or bafilomycin A1 (BafA). (**G**) p62 protein levels normalized to GAPDH in flox/flox, MCM, and cKO CMs treated with DMSO or bafilomycin A1 (*n* = 6). (**H**) LC3I/II ratio in CMs with the indicated genotypes treated with either DMSO or bafilomycin A1 (*n* = 6). [(G) and (H)] Two-way ANOVA with Sidak’s multiple comparison test. (**I**) Normalized LC3II flux assessed by subtracting LC3II protein levels (normalized to GAPDH) in bafilomycin A1–treated versus DMSO-treated CMs (*n* = 6). [(B), (D), and (I)] Welch’s unequal variances *t* test. (**J**) Immunoblotting for p62 and LC3I/II in CMs with the indicated genotypes treated with either DMSO or chloroquine (CQ). (**K**) Immunoblotting for (cleaved) caspase-3 in untreated MCM, flox/flox, and cKO CMs. (**L**) Ratio of cleaved caspase-3 to total caspase-3 in MCM, flox/flox, and cKO CMs (*n* = 3). Unpaired *t* test. [(A), (C), (F), (J), and (K)] GAPDH was used as a loading control. *P ≤ 0.05; **P ≤ 0.01; ***P ≤ 0,001; ****P ≤ 0.0001.

Since the absence of USP5 increases K48-linked ubiquitin chains as well as ubiquitin-containing protein aggregates, we also analyzed K63-linked ubiquitin chains. Immunoblot analysis revealed increased levels of K63-polyubiquitin chains (Fig. 4, A and B) and p62 protein levels (Fig. 4, C and D) in *Usp5*-deficient CMs along with decreased LC3I/II ratios (Fig. 4, C and E). To explore the role of USP5 in autophagy-mediated clearance of protein aggregates in mature CMs, we examined autophagic flux in isolated CMs from cKO and controls, treated with either bafilomycin A1 (Fig. 4, F to I) or chloroquine (Fig. 4J). Chloroquine inhibits autophagic flux by inhibiting the fusion of autophagosomes with lysosomes (*40*), while bafilomycin A1 increases lysosomal pH, preventing the acidification of endosomes and lysosomes and thereby autophagy (*41*, *42*).

Addition of bafilomycin A1 further increased the already elevated levels of the autophagy substrate p62 in *Usp5*-deficient CMs (Fig. 4G) and reduced the LC3I/II ratio (Fig. 4H), which are clear signs for enhanced autophagic flux due to loss of USP5. Likewise, calculation of the autophagic flux [by subtracting glyceraldehyde-3-phosphate dehydrogenase (GAPDH)–normalized LC3II protein levels of dimethyl sulfoxide (DMSO)–treated from bafilomycin A1–treated samples and comparing control and *Usp5*-deficient CMs] revealed a substantial elevation of autophagic flux upon loss of USP5 (Fig. 4I). Similar results were obtained when CMs were treated with chloroquine instead of bafilomycin A1 (Fig. 4J). Chloroquine increased p62 and LC3II protein levels to a higher extent in *Usp5*-deficient compared to flox/flox and MCM CMs (Fig. 4J). Furthermore, *Usp5*-KO CMs showed increased levels of the apoptosis marker cleaved CASP3 (Fig. 4, K and L), suggesting that defects in PQC, initiating enhanced autophagic flux, also increase programmed cell death. We concluded that additional cytotoxic effects caused by the defective UPS in *Usp5*-deficient CMs may contribute to enhanced apoptosis.

### CM-specific inactivation of *Usp5* results in enhanced protein aggregate accumulation and DCM

**Fig. 5.**
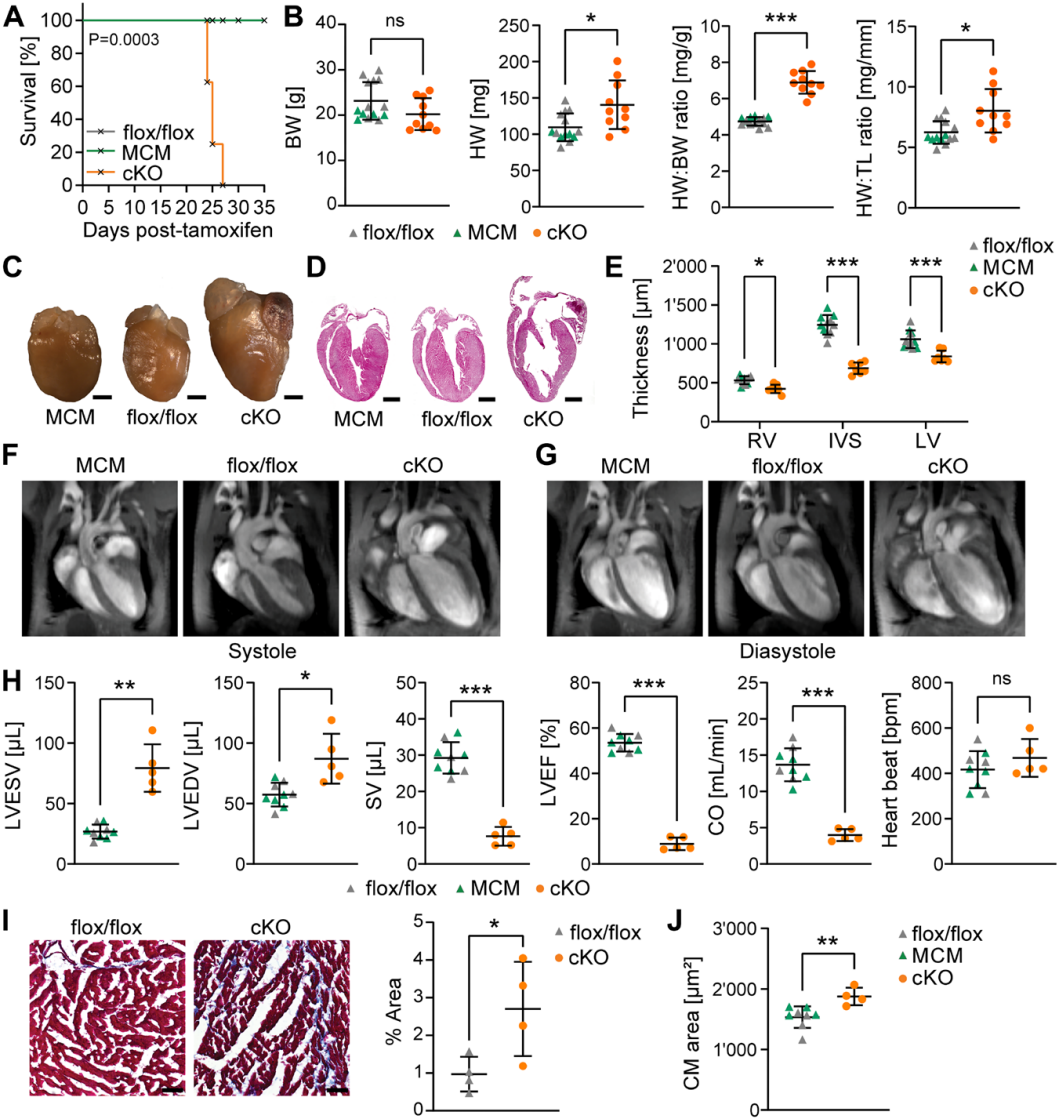
Absence of *Usp5* in CMs causes DCM in mice. (**A**) Kaplan-Meier survival curve of mice with the indicated genotypes after tamoxifen administration. cKO (*n* = 8) died within 2 to 3 days, beginning 24 days after the last tamoxifen injection. None of the control mice (MCM and flox/flox) died after injection of tamoxifen for seven consecutive days. (**B**) Effect of CM-specific *Usp5* deletion on body weight (BW), heart weight (HW), and heart weight to body weight (HW:BW) and heart weight to tibia length (HW:TL) ratio in flox/flox (*n* = 9), MCM (*n* = 5), and cKO (*n* = 10) mice. (**C** and **D**) Representative heart images (C) and H&E-stained heart cross sections of (D). Scale bar, 1000 μm. (**E**) Analysis of myocardial wall thickness (based on H&E-stained cross sections) of hearts from flox/flox (*n* = 6), MCM (*n* = 5), and cKO (*n* = 7) mice. RV, right ventricle; IVS, intraventricular septum; LV, left ventricle. Two-way ANOVA with Sidak’s multiple comparison test. (**F** and **G**) Long-axis views of end-systole (F) and end-diastole (G) of hearts from mice with the indicated genotypes. (**H**) Quantification of cardiac parameters in flox/flox (*n* = 4), MCM (*n* = 5), and cKO (*n* = 5) mice by MRI. ESV, end-systolic volume; EDV, end-diastolic volume; SV, stroke volume; LVEF, left ventricular ejection fraction; CO, cardiac output; HR, heart rate. (**I**) Masson’s trichrome staining of myocardial cross sections from cKO and littermate controls and corresponding quantifications (*n* = 4). Scale bar, 20 μm. (**J**) Quantification of the size of CMs from flox/flox (*n* = 4), MCM (*n* = 4), and cKO (*n* = 4) (for representative images, see Fig. 2D). [(B) and (H) to (J)] Welch’s unequal variances *t* test. For Fig. 5, only male mice were analyzed. *P ≤ 0.05; **P ≤ 0.01; ***P ≤ 0.001.

To explore the consequences of disturbed PQC due to *Usp5* inactivation for cardiac function, we administered tamoxifen for seven consecutive days to 8-week-old mice. All cKO animals died within 28 days after the last tamoxifen administration, but not the MCM and flox/flox controls (Fig. 5A), which was preceded by an increase in heart weight, and higher heart to body weight and heart weight to tibia length ratios (Fig. 5B). cKO mice showed enlarged ventricles, dilated atria, and substantial reduction of ventricular wall thickness (Fig. 5, C to E)—all typical hallmarks of DCM. Cardiac magnetic resonance imaging confirmed cardiac remodeling and progression of severe DCM in cKO mice, as indicated by increased left ventricular end-systolic (LVESV; Fig. 5F) and end-diastolic volumes (LVEDV; Fig. 5G), reduced stroke volume (SV), left ventricular ejection fraction (LVEF), and cardiac output (CO; Fig. 5H). The heart rate (HR) was not altered in cKO compared to the controls. Moreover, cKO hearts were fibrotic (Fig. 5I) and their CMs were profoundly hypertrophic (Figs. 2D and 5J). Further analysis of heart sections revealed an increase in the amount and size of ProteoStat-stained aggregates in cKO CMs (fig. S6, A to C). We also detected disrupted sarcomeres by electron microscopy (fig. S6D) and accumulation of lysosomal structures in cKO but not in control CMs (fig. S6, E and F). Together, the analysis uncovered pathological alterations in *Usp5*-deficient CMs, associated with rapid progression from DCM to heart failure in cKO mice.

### Human DCM hearts are characterized by reduced USP5 protein levels and accumulation of ubiquitin-positive protein aggregates

**Fig. 6.**
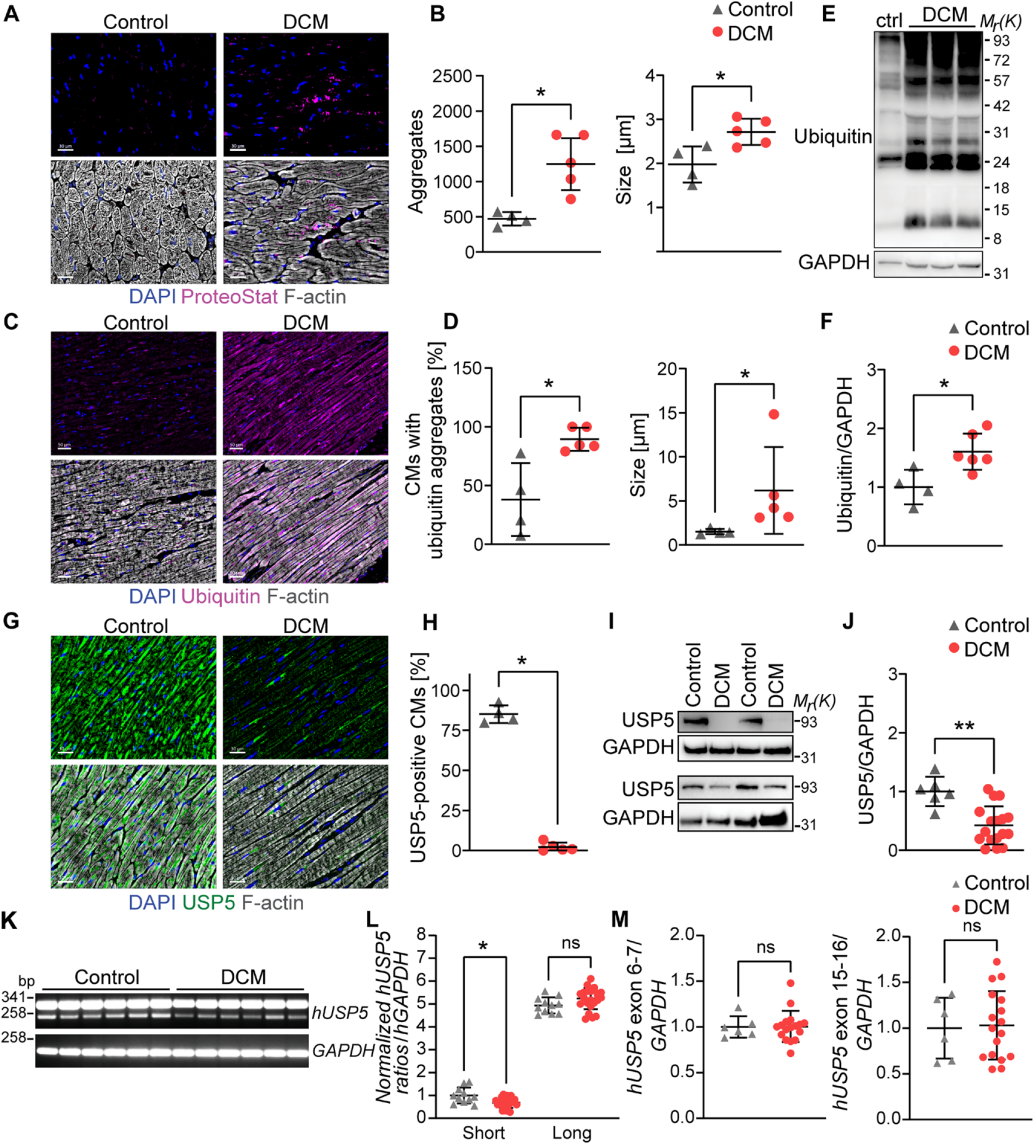
End-stage DCM in humans is characterized by reduced USP5 levels and increased ubiquitin-conjugated protein aggregates. (**A**) Staining for protein aggregates (ProteoStat, magenta), F-actin (gray), and nuclei (DAPI, blue) in cross-sectioned hearts from control and DCM persons. Scale bar, 30 μm. (**B**) Number and size of protein aggregates in control (*n* = 4) and DCM (*n* = 5) hearts. (**C**) Staining for ubiquitin (magenta), F-actin (gray), and nuclei (DAPI, blue) in cross-sectioned hearts from control and DCM individuals. Scale bar, 50 μm. (**D**) Percent of CMs containing ubiquitin-positive aggregates and corresponding size in hearts of control (*n* = 4) and DCM (*n* = 5) individuals. (**E** and **F**) Immunoblotting (E) and corresponding quantification (F) for ubiquitin in hearts from control (*n* = 4) and DCM (*n* = 6) individuals normalized to GAPDH. (**G**) Staining for USP5 (green), F-actin (gray), and nuclei (DAPI, blue) in heart cross sections from control and DCM individuals. Scale bar, 30 μm. (**H**) Percentage of USP5-positive CMs in control (*n* = 4) and DCM (*n* = 5) myocardium. (**I** and **J**) Immunoblotting of control and DCM hearts (I) and corresponding quantification (J) for USP5 in lysates from control (*n* = 6) and DCM hearts (*n* = 17) normalized to GAPDH. [(B), (D), (F), (H), and (J)] Welch’s unequal variances *t* test. (**K**) RT-PCR analysis of *hUSP5* splice variants in human control and DCM myocardium. *GAPDH* was used as loading control. (**L**) Quantification of short and long *hUSP5* in control (*n* = 11) and DCM (*n* = 23) myocardium, normalized to *GAPDH*. One-way ANOVA. (**M**) RT-qPCR of *hUSP5* exon boundaries 6 to 7 and exons 15 and 16 in control (*n* = 6) and DCM (*n* = 17) myocardium, normalized to *GAPDH*. Mann-Whitney test. [(E) and (I)] GAPDH was used as a loading control. *P ≤ 0.05; **P ≤ 0.01.

To investigate whether hUSP5-dependent processes play a role for progression of DCM in humans, we investigated a cohort of end-stage DCM patients undergoing heart transplantation. ProteoStat staining revealed an increase in the size and number of protein aggregates in CMs from DCM patients compared to controls (Fig. 6, A and B). These data are consistent with the hypothesis that DCM progression is accompanied by abnormal accretion of ubiquitin-positive protein aggregates in CMs (*43*). We also observed enhanced ubiquitin depositions by immunofluorescence staining (Fig. 6, C and D) and an increase of ubiquitinated proteins by immunoblotting in DCM myocardium (Fig. 6, E and F). We observed a significant reduction of hUSP5 in CMs of DCM patients by immunofluorescence staining compared to controls (Fig. 6, G and H). Western blot analysis confirmed an approximately 60% decrease of hUSP5 concentrations in a larger cohort of DCM patients, compared to control hearts (Fig. 6, I and J). In contrast to the reduction of USP5 protein levels, RT-PCR did not detect a lower expression of the long isoform of *hUSP5*, which is predominant in the human myocardium (Fig. 6, K and L). However, we observed a reduction in the expression of the short *hUSP5* isoform in DCM patients (Fig. 6M), which is probably caused by a change in cell composition, e.g., increased presence of fibroblasts and infiltrating cells. RT-qPCR ruled out changes in the overall expression of *hUSP5* (combination of long and short isoforms) (Fig. 6M), indicating a posttranscriptional regulation of USP5 in DCM. The results demonstrate a striking similarity to the mouse model. In mice, inactivation of *Usp5* results in DCM. In humans, the reduced presence of hUSP5 is associated with DCM.

### Cardiac-restricted overexpression of USP5 attenuates adverse effects of pressure overload in mice

**Fig. 7.**
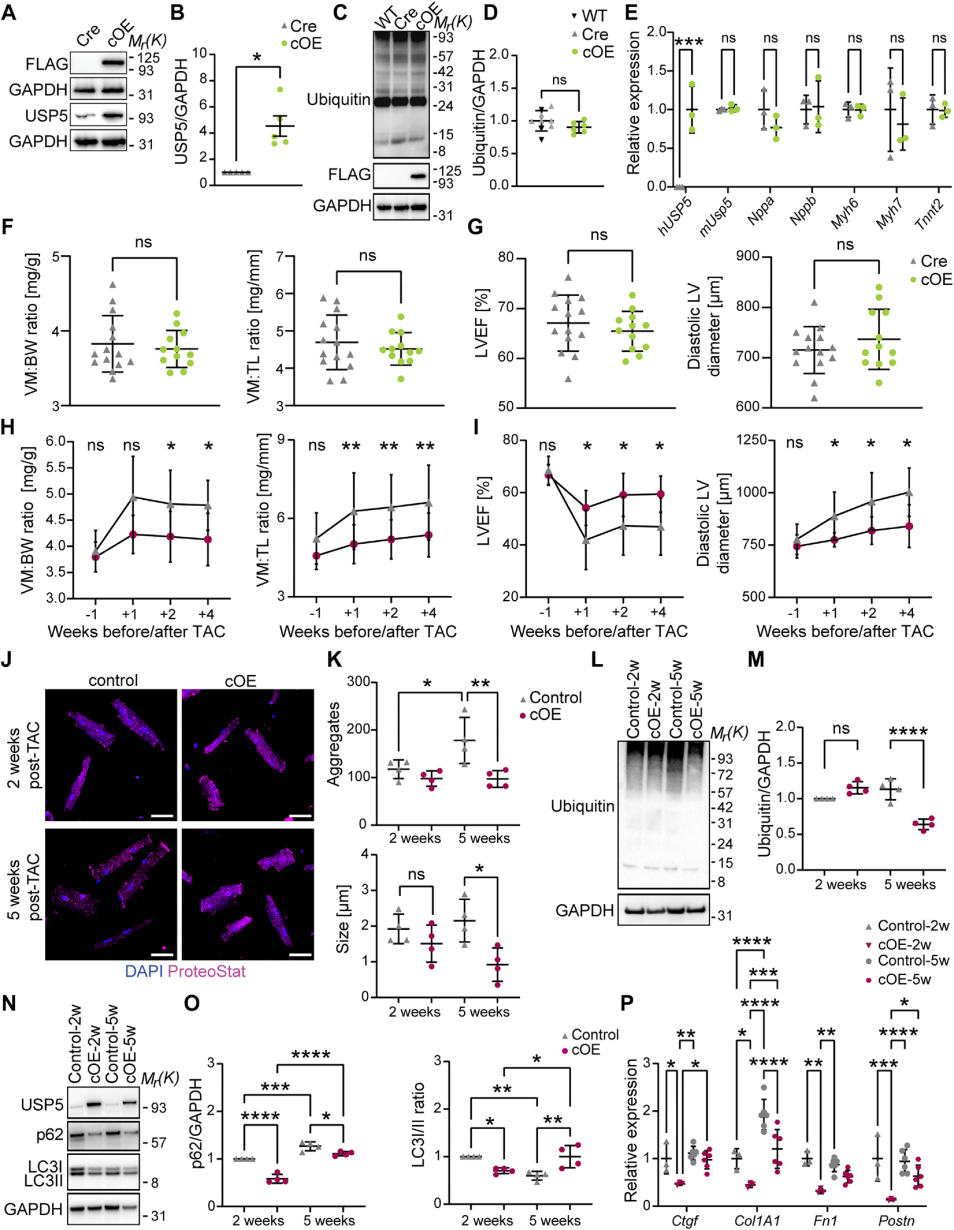
Overexpression of *USP5* attenuates pressure overload–induced cardiac hypertrophy. (**A** and **B**) Immunoblotting (A) and quantification (B) for USP5 in Cre and cOE CMs (*n* = 5). (**C** and **D**) Immunoblotting (C) and quantification (D) for ubiquitin in WT (*n* = 6), Cre (*n* = 3), and cOE (*n* = 6) CMs. The same membrane was reprobed with anti-FLAG and anti-GAPDH antibodies. (**E**) RT-qPCR analysis of *hUSP5*, *mUsp5*, and stress-response genes in Cre and cOE CMs (*n* = 3). Two-way ANOVA with Sidak’s multiple comparison test. (**F** and **G**) Heart function parameters in Cre (*n* = 14) and OE (*n* = 12) mice. Ventricular mass to body weight (VM:BW) and ventricular mass to tibia length (VM:TL) ratios (F), left ventricular ejection fraction (LVEF), and diastolic left ventricular (LV) diameter (G). (**H** to **P**) TAC surgeries were conducted with mice aged 14 ± 1 weeks. Control (*n* = 9, 5 females, 4 males), cOE (*n* = 8, 5 females, 3 males). [(H) and (I)] The cardiac function was monitored using MRI at −1, 1, 2, and 4 weeks relative to surgery: VM:BW and VM:TL ratios (H) and LVEF and LV diameter (I) (*n* ≤ 9). Mixed-effect analysis with Tukey’s multiple comparison test. [(J) to (P)] Molecular analyses were performed 2 and 5 weeks after TAC surgery. [(J) and (K)] ProteoStat staining (J) for aggregates (magenta) and nuclei (DAPI, blue) and number and size of protein aggregates in control and cOE CMs (*n* = 4). [(L) to (P)] Immunoblotting [(L) and (N)] and quantification [(M), (O), and (P)] for ubiquitin, USP5, p62, and LC3 levels in control and cOE CMs (*n* = 4). (P) RT-qPCR analysis of fibrosis marker genes in non-CMs from control and cOE hearts (2 weeks, *n* = 3; 5 weeks, *n* = 6). [(A), (C), (L), and (N)] GAPDH was used as a loading control. [(B), (D), (F), and (G)] Mann-Whitney test. [(K), (M), (O), and (P)] Two-way ANOVA with Tukey’s multiple comparison test. *P ≤ 0.05; **P ≤ 0.01; ***P ≤ 0.001, ****P ≤ 0.0001.

The reduction of hUSP5 in CMs of human DCM patients and the necessity of USP5 for maintaining PQC in mouse hearts raised the intriguing possibility that USP5 might be rate limiting to prevent or resolve protein aggregate formation in CMs. To explore this hypothesis, we created a transgenic mouse strain that conditionally expresses the FLAG-tagged long human *USP5* isoform from the ROSA26 locus after removal of a floxed stop cassette by *XMLC2-cre*. *XMLC2-cre^+/-^;hsUSP5^cOE/+^* mice (hereafter called cOE) were compared to *XMLC2-cre* mice (hereafter called Cre). CM-restricted *hUSP5* overexpression was verified by immunoblotting (Fig. 7, A and B) and RT-qPCR (Fig. 7E) in cOE CMs. Overexpression of *Usp5* had no effects on the ubiquitin pool (Fig. 7, C and D). In addition, we did not find an increase of cardiac stress markers in CMs (Fig. 7E). Cardiac functions were normal (Fig. 7F), and gross morphology of cOE hearts was unaltered (fig. S7E).

To explore potential protective effects of USP5 overexpression, we turned to the pressure-overload model, which is characterized by increased proteotoxic stress, inducing formation of intracellular protein aggregates, and thereby triggering activation of proteasomal and autophagic clearance pathways (*4*, *44*). To initiate pressure overload, transverse aortic constriction (TAC) surgeries were conducted in 14 ± 1–week–old control and cOE mice. No differences in body weights were observed between control and cOE mice after TAC surgery (fig. S7A), and cardiac hypertrophy was induced in both genotypes, as indicated by an increase in ventricular mass relative to both body weight and tibia length (fig. S7, B and C). However, the degree of hypertrophy was consistently lower in cOE mice compared to controls (Fig. 7H and fig. S7, B and F). cOE mice showed a significantly better LVEF after TAC compared to controls (Fig. 7I and fig. S7C), suggesting a beneficial effect of *USP5* overexpression on heart function after TAC. Moreover, the myocardium of cOE mice showed reduced wall thickening (Fig. 7I and fig. S7, C and D), although cOE mice were not completely protected from hypertrophic remodeling.

Two to 5 weeks after TAC, immunofluorescence staining revealed increased formation of protein aggregates in control and cOE groups. However, 5 weeks after TAC, cOE hearts showed fewer and smaller aggregates compared to controls (Fig. 7, J and K). We also detected lower amounts of ubiquitinated proteins 5 weeks after TAC (Fig. 7, L and M), which suggested that *USP5* overexpression promotes protein clearance or prevents formation of aggregates. p62 levels were up-regulated during pressure overload in control mice, whereas the LC3I/II ratio was decreased (Fig. 7, N and O), indicating enhanced autophagic activity in response to proteotoxic stress. In contrast, p62 levels were substantially lower in cOE CMs, indicating attenuated maladaptive autophagic responses (*45*). We also observed reduced expression of the fibrosis genes *Ctgf*, *Col1a1*, *Fn1*, and *Postn* in the non-CM cell fraction, 2 and 5 weeks after TAC (Fig. 7P). Together, our findings suggest that CM-specific overexpression of *USP5* reduces pressure overload–induced cardiac hypertrophy and promotes clearance of protein aggregates, thereby reducing activation of autophagic responses.

### Increased USP5 expression decreases protein aggregates in titinopathy and desminopathy models

**Fig. 8.**
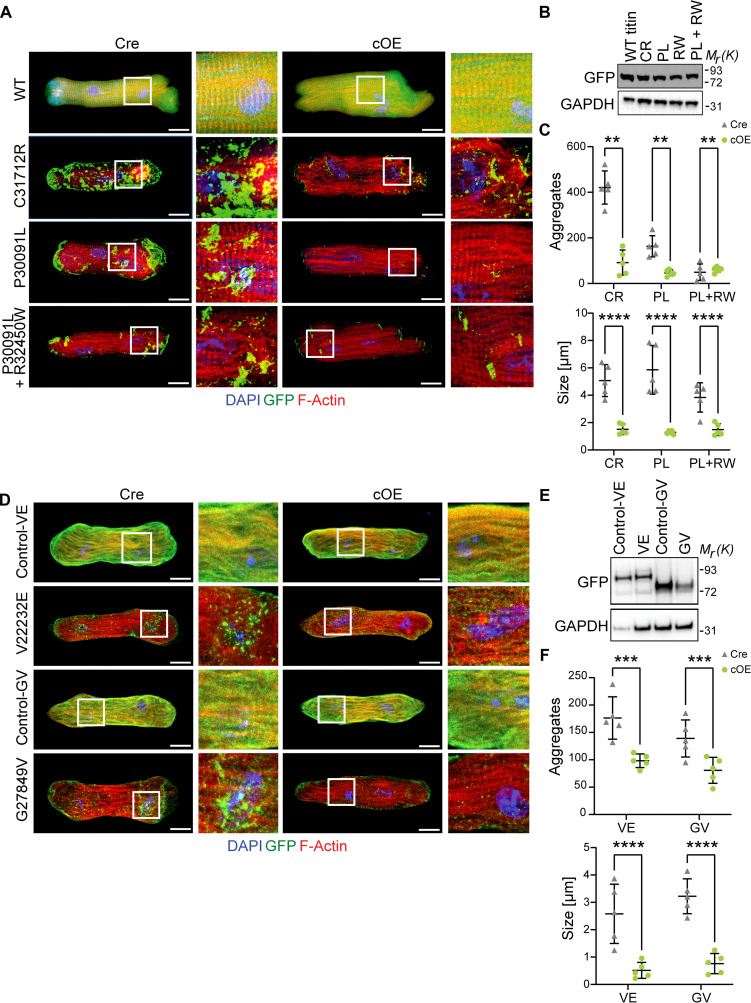
Augmented expression of *USP5* abolishes mutant titin-containing protein aggregates in CMs. (**A**) Staining for GFP-titin (green), F-actin (red), and nuclei (DAPI, blue) of CMs transduced with an adenovirus encoding WT titin and HMERF-linked mini-titins C31712R (CR), P30091L (PL), and P30091L + R32450W (PL + RW). Magnification of marked image sections is shown next to it. Scale bar, 25 μm. (**B**) Protein levels of GFP-tagged HMERF-linked mini-titins determined by immunoblotting. (**C**) Number and size of GFP-positive aggregates in Cre and cOE CMs (*n* = 5). (**D**) Staining for GFP-titin (green), F-actin (red), and nuclei (DAPI, blue) in CMs transduced with an adenovirus encoding DCM-linked mini-titins p.Val22232Glu (VE), p.Gly27849Val (GV), and corresponding controls. Magnification of marked image sections is shown. Scale bar, 25 μm. (**E**) Immunoblot analysis of GFP-tagged DCM-linked mini-titin protein levels. (**F**) Number and size of GFP-positive aggregates in Cre and cOE CMs (*n* = 5). [(C) and (F)] Two-way ANOVA with Tukey’s multiple comparison test. [(B) and (E)] GAPDH was used as a loading control. **P ≤ 0.01; ***P ≤ 0.001, ****P ≤ 0.0001.

We demonstrated that enhanced expression of USP5 reduces adverse hypertrophic remodeling in pressure-overloaded hearts by attenuating proteotoxic stress. Next, we wanted to know whether USP5 exerts similar effects in specific proteinopathies, such as titinopathy and desminopathy. In pursuit of this goal, we generated a model for HMERF (hereditary myopathy with early respiratory failure), a titinopathy that shows protein aggregate formation and ubiquitin-, p62-, and LC3-positive rimmed vacuoles (*46*). HMERF is predominantly linked to missense variants in the A150 (Fn3-119) domain of titin, of which the C31712R and P30091L, as well as the compound heterozygous P30091L + R32450W variants, are the most common forms, causing protein destabilization and aggregation (*46*–*48*). The C31712R mutation is tightly linked with a cardiac phenotype in HMERF patients, showing increased incidence of atrial fibrillation (*49*). Expression of the HMERF-linked mini-titins induced formation of protein aggregates in CMs, whereas expression of WT green fluorescent protein (GFP)–tagged titin fragments did not (Fig. 8A), although protein levels of mutant and WT mini-titins were comparable (Fig. 8B). Strikingly, the size and number of titin aggregates were much lower in *hUSP5*-overexpressing CMs (Fig. 8C), indicating that elevated levels of USP5 abrogate protein aggregate formation. To obtain further evidence for the beneficial role of enhanced USP5 expression in titinopathies, we also studied two titin variants that are directly implicated in human DCM: p.Val22232Glu and p.Gly27849Val (*50*, *51*). Expression of the p.Val22232Glu and p.Gly27849Val variants but not the control mini-titins robustly induced aggregate formation in control CMs (Fig. 8, D to F). In contrast, aggregate formation was strongly reduced in *hUSP5*-overexpressing CMs (Fig. 8, D to F), essentially recapitulating the effects observed with the HMERF-linked mini-titins.

**Fig. 9.**
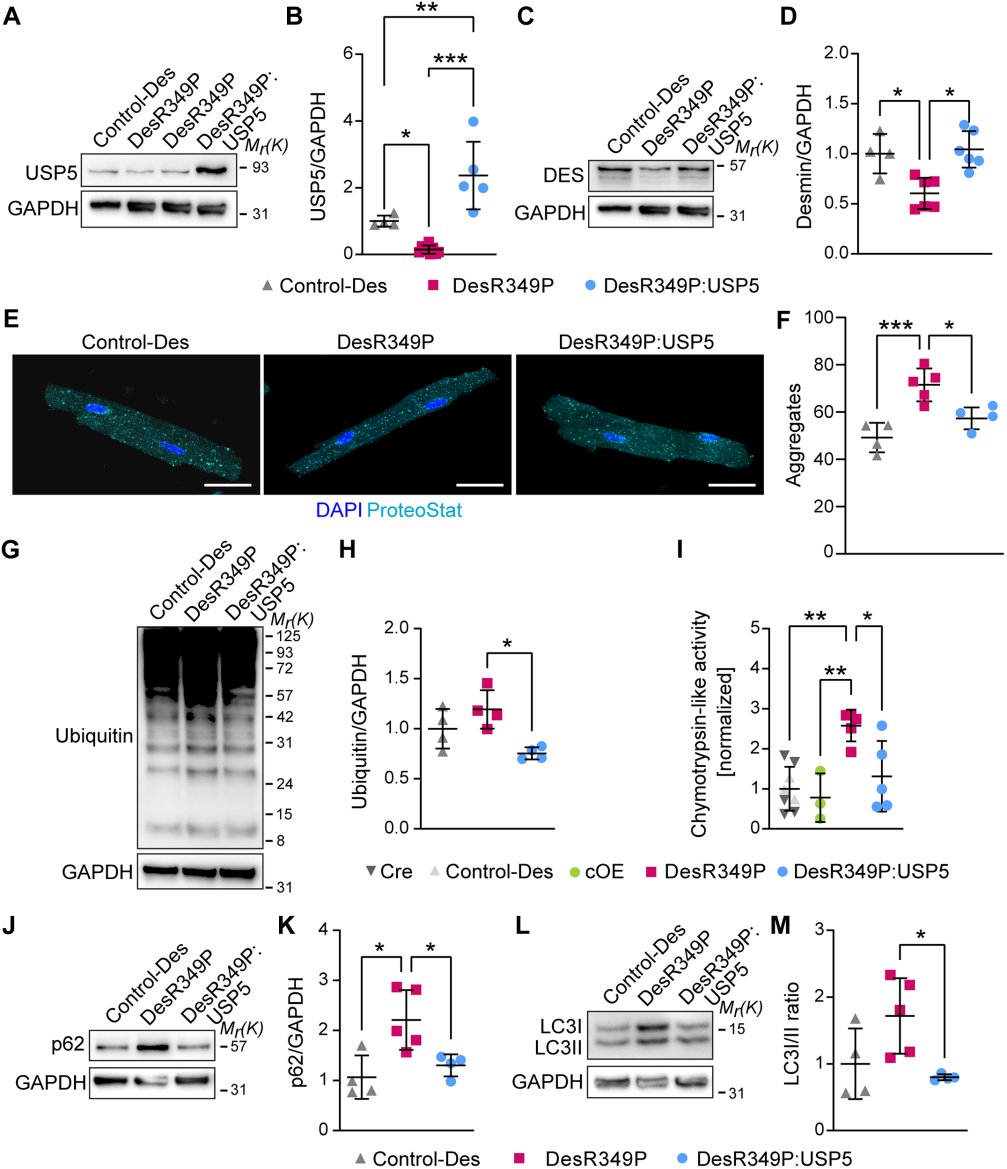
Elevated expression of *USP5* restores PQC defects in CMs expressing mutant desmin. (**A** to **D**) Immunoblotting and corresponding quantifications for USP5 [(A) and (B)] and desmin [(C) and (D)] in CMs of Control-Des (*n* = 4), DesR349P (*n* ≥ 6), and DesR349P:USP5 (*n* ≥ 5) mice normalized to GAPDH. (**E**) Staining for protein aggregates (ProteoStat, cyan) and nuclei (DAPI, blue) in CMs with the indicated genotypes. Scale bar, 25 μm. (**F**) Number of protein aggregates in Control-Des (*n* = 4), Des-R349P (*n* = 5), and DesR349P:USP5 (*n* = 4) CMs. (**G**) Immunoblotting for ubiquitin in Control-Des, DesR349P, and DesR349P:USP5 CMs. (**H**) Ubiquitin levels in Control-Des, DesR349P, and DesR349P:USP5 CMs (*n* = 4, each). (**I**) Chymotrypsin-like activity in control (*n* = 8), cOE (*n* = 3), R349P-Des (*n* = 5), and R349P-Des:USP5 (*n* = 5) CMs determined using AMC. Control groups include Control-Des (*n* = 3) and Cre (*n* = 5). (**J** and **L**) Immunoblotting for p62 (J) and LC3 (L) in Control-Des, DesR349P, and DesR349P:USP5 CMs. (**K** and **M**) Protein levels of p62 (K) and LC3I/II ratio (M) normalized to GAPDH in CMs of Control-Des (*n* = 4), DesR349P (*n* = 5), and DesR349P:USP5 (*n* = 4) mice. [(A), (C), (G), (J), and (L)] GAPDH served as a loading control. [(B), (D), (F), (H), (I), (K), and (M)] One-way ANOVA with Tukey’s multiple comparison test. *P ≤ 0.05; **P ≤ 0.01; ***P ≤ 0.001.

To further investigate the potency of increased *hUSP5* expression for reduction of protein aggregate formation in specific proteinopathies, we also examined an in vivo model of desminopathy. We used homozygous *R349P desmin* knock-in mice (hereafter called *DesR349P*), which harbor the orthologue of the most frequent human desmin missense mutation *DesR350P*, showing (ubiquitinated) protein aggregates and enhanced proteasome activity (*52*–*54*). *R349P desmin* knock-in mice were analyzed at time points (4 to 6 months), at which aggregates are clearly present but signs of DCM have not yet developed, thereby avoiding secondary effects from DCM (*55*). We found decreased levels of USP5 in *DesR349P* compared to control CMs (Fig. 9, A and B), similar to the diminished USP5 protein abundance in human end-stage DCM patients. Overexpression of *hUSP5* normalized desmin protein levels, which were reduced in hearts of 4- to 6-month-old homozygous *DesR349P* mutant mice (Fig. 9, C and D), and also normalized the number of protein aggregates in DesR349P CMs (Fig. 9, E and F), indicating prevention of aggregate formation or enhanced clearance. Moreover, polyubiquitin levels were lowered in *DesR349P:USP5* compared to *DesR349P* CMs (Fig. 9, G and H) and overexpression of *hUSP5* reduced the increased CT-L peptidase activity of the proteasome in *DesR349P* CMs, which now matched activities in control and cOE CMs (Fig. 9I). We also investigated a potential impact of *hUSP5* overexpression on the increased autophagic flux in desmin-related cardiomyopathies (*54*, *56*). Overexpression of *hUSP5* restored levels of the autophagy marker p62 (Fig. 9, J and K) and normalized the LC3I/II ratio (Fig. 9, L and M), indicating that overexpression of USP5 in CMs alleviates protein aggregation and various aspects of PQC in desminopathy, before the onset of DCM. In conclusion, overexpression of *hUSP5* prevents formation of not only protein aggregates forming during pressure overload but also aggregates that emerge as a consequence of specific mutations in titin and desmin.

## DISCUSSION

Dysfunction of the UPS and/or autophagy pathways plays important roles in various diseases, but the specific function of individual components of the UPS machinery for the pathogenesis of DCM is still mostly elusive (*15*). Here, we provide evidence that the ubiquitin-specific peptidase USP5 is critical for maintaining ubiquitin-dependent proteostasis in CMs. We demonstrated that inactivation of *Usp5* in mouse CMs leads to accumulation of protein aggregates and DCM, whereas increased levels of *hUSP5* in CMs attenuate pressure overload–induced cardiac hypertrophy and promote clearance of protein aggregates. Similarly, overexpression of *hUSP5* reduces protein aggregates in models of titinopathy and desminopathy. Our findings correspond well to previous biochemical studies, demonstrating that USP5 is an important enzyme for the cleavage of polyubiquitin chains, critical for preventing accumulation of free polyubiquitin chains, which disrupt ubiquitin recycling and potentially interfere with normal PQC (*25*). In line with previous reports (*11*, *22*), we defined a central function of USP5 for rapid trimming of PSMD14-released polyubiquitin chains, keeping the 26*S* proteasome free of ubiquitin chains, which otherwise would promote proteasomal degradation (*54*).

Inactivation of *Usp5* altered ubiquitin levels and results in protein aggregation primarily in mature CMs but to a lower extent in proliferating HL-1 CMs, MEFs, myocardial non-CMs, and hepatocytes. The requirement of USP5 for sustained UPS-dependent proteolysis in postmitotic CMs corresponds to the presence of the long *Usp5* isoform, which is enriched in postmitotic cells. We assume that the differential requirement of USP5 depends on different levels of mono- and polyubiquitin and the presence of other DUBs, which may partially compensate for the absence of USP5. While the ubiquitin pool in MEFs contains ~11% polyubiquitin chains, the mouse brain (*57*) and the human frontal cortex (*58*) contain only ~4 to 5% polyubiquitin chains, which might suggest that postmitotic cells such as CMs are less tolerant for longer polyubiquitin chains and require efficient disassembly of polyubiquitin to maintain proteasome function. In CMs, such efficient disassembly of polyubiquitin apparently relies on the presence of USP5, enabling the proteasome to handle cell type–specific proteolytic demands (*59*).

We noted that loss of USP5 enhances assembly and activity of the proteasome, probably induced by accumulation of ubiquitinated proteins. However, the compensatory increase in proteasome assembly and activity is not sufficient to clear the accumulation of ubiquitinated proteins resulting from the failure to efficiently disassemble long poly-ubiquitin chains, leading to increased formation of protein aggregates. Such imbalances also occur in pathological conditions such as pressure-overloaded hypertrophic hearts. The proteasome activity increases during cardiac hypertrophy, responding to the need for enhanced protein turnover (*60*), but is not sufficient to completely prevent aggregate formation pathways (*4*). Increased 26*S* proteasome activity has also been reported for patients with left ventricular hypertrophy and DCM (*61*). We observed a clear attenuation of adverse hypertrophic remodeling in hUSP-overexpressing mice subjected to pressure overload, which was accompanied by reduced aggregate formation and reduced maladaptive autophagic responses. Previous studies demonstrated that amplification of autophagic response to pressure overload increases hypertrophic growth of the heart, which seems to identify enhanced autophagy as a driver of hypertrophy (*4*, *45*). However, removal of protein aggregates by enhanced proteasomal and autophagy activity will also reduce proteotoxicity, causing maladaptive responses. Thus, it is currently difficult to distinguish whether normalization of PQC pathways in *hUSP5*-overexpressing hearts or improved removal of proteotoxic aggregates is responsible for the improved outcome.

The elevated autophagic flux after inactivation of *Usp5* in CMs might be a compensatory response of CMs, caused by the dysregulated UPS to cope with increased load of protein aggregates. An alternative explanation is the direct stimulation of autophagy by accumulation of K63-linked (poly-)ubiquitin chains. USP5 is well known to cleave not only K48- but also K63-linked chains (*27*), which correspond to the increased concentration of K63-linked (poly-)ubiquitin chains in *Usp5*-deficient CMs. However, the profound imbalance of the cellular ubiquitin pool, initiated by loss of USP5, results in the accumulation of ubiquitinated protein aggregates, essentially rendering stimulation of proteasomal and autophagic activities useless.

The increase of ubiquitinated protein aggregates and the development of lethal DCM in cKO mice shows clear parallels to human cardiomyopathies where similar changes have been observed (*4*, *62*). Specifically, adaptive cardiac hypertrophic remodeling increases proteasome activity and 26*S* proteasome assembly, whereas progressive heart failure in human patients is accompanied by loss of proteasome functions (*63*). We found that human patients with end-stage DCM have reduced USP5 protein but not mRNA levels together with the increased presence of polyubiquitin chains and ubiquitinated protein. The diminished levels of USP5 proteins are apparently not caused by reduced *hUSP5* gene activity but by posttranscriptional mechanisms, since the mRNA level of *hUSP5* does not decline. Reduced USP5 levels (isopeptidase T) have previously been reported by Kostin *et al*. in idiopathic DCM patients (*64*). However, re-evaluation of the published data reveals that the claim for reduced levels of USP5 in human DCM was based on the decline of a 24-kDa protein band, which does not correspond to the molecular weight of USP5 (96 kDa) (*64*). Although it is currently not clear whether the down-regulation of USP5 during DCM is simply the consequence of an overloaded and deregulated PQC system, we reason that depletion of USP5 during DCM further compromises the ability of CMs to cope with increased PQC stress and thus further enhances protein aggregate formation. This view is supported by the ability of USP5 to attenuate pressure overload–induced cardiac hypertrophy and prevent or revert formation of protein aggregates occurring in titinopathy and desminopathy models. We also found a down-regulation of USP5 in CMs of homozygous p.R349P desmin knock-in mice, carrying the orthologue of the most frequent human desmin missense mutation p.R350P. The low expression of USP5 in CMs of DesR349P knock-in mice may expedite enhanced formation of protein aggregates, alter proteasome activity, and impair autophagy (*53*, *65*).

**Fig. 10.**
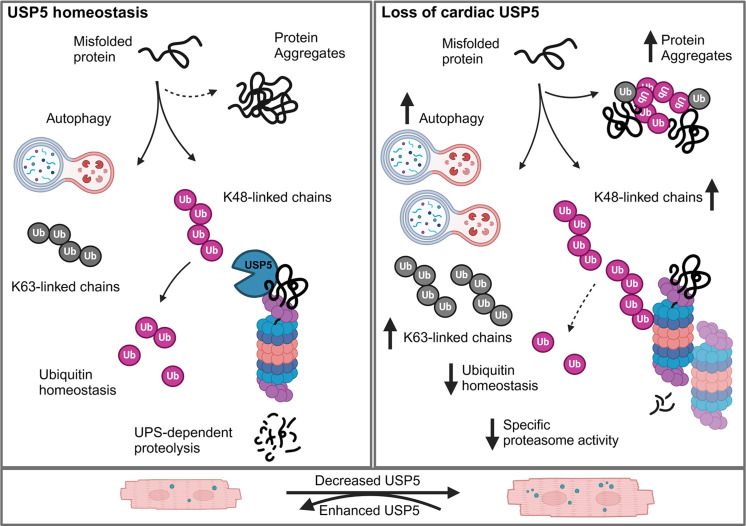
Schematic representation of the effects of USP5 in PCQ of CMs. Model of the role of USP5 in cardiac PQC. Schematic representation of the effects of USP5 inactivation on proteostasis in CMs. (**Left**) At steady state, USP5 maintains PQC and proteostasis by the disassembly of free polyubiquitin chains. (**Right**) Loss of USP5 in CMs alters PQC due to an imbalance in the ubiquitin pool, which increases autophagy and proteasome activity, resulting in elevated protein aggregation. (**Bottom**) Augmented expression of USP5 abolishes protein aggregates in CMs after pressure overload or in proteinopathies.

We reason that USP5 maintains PQC and proteostasis under steady-state conditions by disassembly of free polyubiquitin chains (Fig. 10, left panel), thereby promoting proteolysis of misfolded, damaged, or non-used proteins. Loss of USP5 in CMs alters PQC and dysregulates UPS-dependent proteolysis due to an imbalance in the ubiquitin pool, reducing specific proteasomal activity despite an increase in proteasomal protein levels and total enzymatic activity (Fig. 10, right panel). Manipulations to increase USP5 concentrations, either by overexpression or by preventing its down-regulation in DCM conditions, seem to have a therapeutic potential, alternative and complementary to approaches targeting specific gene defects (*66*). Overexpression of *hUSP5* in CMs appears to be safe, not causing any adverse effects in the cardiovascular system. Increased levels of hUSP5 in CMs efficiently prevented malfunctions of the PQC machinery and reduced abnormal protein aggregate formation in pressure overload–induced cardiac hypertrophy as well as in models of titinopathy and desminopathy, which gives hope for the future. Nevertheless, extensive further research is necessary to determine whether forced or induced expression of USP5 improves clinical manifestations of cardiomyopathy.

## MATERIALS AND METHODS

### Genetically modified and WT mice

Animals were maintained in a specific pathogen–free environment of the mouse facility of the Max Planck Institute for Heart and Lung Research in Bad Nauheim, Germany. Compliance with institutional guidelines and German animal welfare laws was assured for all animal health care and experimental procedures after approval from the local governmental animal protection committee (Regierungspräsidium Darmstadt; B2/1136, B2/2018, and B2/2053). C57BL/6J WT mice are bred at this institute. Mice were euthanized using either CO_2_ or ketamine-xylazine overdose. Organ and embryo harvest procedures were conducted postmortem, unless stated otherwise. Age-matched mice or littermates were used for each experiment, unless stated otherwise. The number of animals for each experiment is specified in the figure legends.

The *Usp5* conditional KO allele was generated in V6.5 embryonic stem (ES) cells using the commercially available targeting construct *Usp5*^*tm1a*(*EUCOMM*)*Hmgu*^ (EuMMCR), which inserts two loxP sites flanking exons 3 to 6. Selection of targeted ES clones was accomplished with G418, followed by Southern blot analysis using the restriction enzyme Sma I. Verified, targeted ES cell clones were introduced into blastocysts from C57BL/6 (B6) mice. Chimeric *Usp5^flLacZneo/+^* mice were mated with transgenic mice expressing *FLPe* recombinase (*67*) to remove the neomycin selection and LacZ cassettes from the targeted allele, resulting in *Usp5flox/flox* mice. The mice were backcrossed to C57BL/6J.

CM-specific inactivation of *Usp5* was achieved by breeding of *Usp5flox/flox* mice with *Myh6-MerCreMer* (*68*), which harbor the cardiac-specific α-myosin heavy chain promoter directing expression of a tamoxifen-inducible *Cre* recombinase. Control animals were age-matched littermates without *Cre* expression (*flox/flox*) and littermates expressing *Myh6-MerCreMer* (MCM). Animals received injections of tamoxifen (100 mg kg^−1^, intraperitoneally) for 7 days, starting 8 weeks after birth.

The allele for conditional overexpression of the human *USP5* exon 15 long isoform was generated by applying a ROSA26 targeting construct (*69*), in which a synthetic CAG promoter replaced the SA-site of pBigT. The human Flag–hemagglutinin (HA)–*USP5* exon 15 long isoform was PCR-amplified from heart cDNA.

V6.5 ES cells received the final construct by electroporation. Selection of targeted stem cell clones was achieved by G418 treatment, followed by screening via Southern blot analysis using the Eco RV restriction enzyme. C57BL/6 (B6) blastocysts were injected with correctly targeted ES cell clones. Resulting progeny were backcrossed to WT C57BL/6J mice to generate conditional, heterozygous mice (*hsUSP5^cOE/+^*). *hsUSP5^cOE/+^* mice were cross-bred with *XMLC2-cre* transgenic mice (*70*) to allow conditional overexpression of *USP5* in CMs (*XMLC2-cre^+/-^;hsUSP5^cOE^*). Age-matched WT littermates expressing *XMLC2-cre* (Cre) were used as controls.

Homozygous DesR349P (c.1045_1047delAGG>insCCC) desmin knock-in mice (B6J.129Sv-*Des*^tm1.1Ccrs^; http://www.informatics.jax.org/allele/MGI:5708562) and DesR349P:*USP5* were analyzed at an age of 4 to 6 months (*65*, *71*, *72*).

Since no significant differences were present between male and female mice, both sexes were combined into single groups for subsequent analyses. One exception occurred. For experiments shown in Fig. 5, only male mice were used for technical reasons. We exclusively used aged-matched mice for comparisons.

### Isolation of murine embryonic and postnatal CMs

CMs were isolated from embryos at embryonic day 13.5 (E13.5) or from neonates at postnatal day 0 to 1 (P0 to P1) using a combination of the Neonatal Heart Dissociation Kit and the Mouse Neonatal CM Isolation Kit from Miltenyi Biotec.

### Isolation of adult CMs and myocardial non-CMs

Isolation of adult CMs was carried out as previously described (*73*). Briefly, dissected hearts were cannulated via the aorta and retrogradely perfused with calcium-free buffer. Hearts were enzymatically digested by perfusion with enzyme buffer and cut off from the cannula. Ventricles were minced in stop buffer, followed by gentle pipetting to create cell suspensions. After centrifugation for 1 min at 500 rpm, myocardial non-CMs were collected from the supernatant, whereas stop buffer was used to resuspend CM-containing cell pellets. The calcium content of the cell suspension was gradually increased to 1 mM. For culturing, adult CMs were resuspended in M199 cell culture and maintained at 37°C in a humidified environment at 5% CO_2._

### RNA isolation, cDNA synthesis, RT-PCR, and RT-qPCR

RNA was extracted using the Direct-zol RNA Kit according to the manufacturer’s protocol. RNA was reverse-transcribed using the Invitrogen SuperScript II Reverse Transcriptase or the PrimeScript RT Reagent Kit with gDNA Eraser from Takara. RT-qPCR was done with TaqMan Gene Expression Assays on a StepOnePlus Real-Time PCR System with TaqMan Gene Expression Assays indicated below. Technical triplicates were run for each sample, and relative mRNA values were determined using the ∆∆*C*_t_ method with normalization to *Gapdh* or *Actb* as internal controls.

### TaqMan gene expression assays

TaqMan gene expression assays were used as follows: *mActa1* Mm00808218_g1, *mCtgf* Mm01192933_g1, *mCol1A1* Mm00801666_g1, *mFbxo32* Mm00499523_m1, *mFn1* Mm01256744_m1, *mMyh6* Mm00440359_m1, *mMyh7* Mm00600555_m1, *mNppa* Mm01255748_g1, *mNppb* Mm01255770_g1, *mPsmd4* Mm01263490_m1, *mPostn* Mm01284919_m1, *mUbb* Mm01622233_g1, *mUbc* Mm02525934_g1, *mUchl1* Mm00495900_m1, *mUsp5* Mm00496734_g1, and *mTnnt2* Mm01290254_g1, and normalized to *mGapdh* Mm99999915_g1 or *mActb* Mm00607939_s1. For human transcripts, the following TaqMan gene expression assays were used: *hUSP5* exons 6 and 7 Hs00185822_m1, *hUSP5* exons 14 and 15 Hs01088041_m1, and *hUSP5* exons 15 and 16 Hs01092106_g1, and normalized to *hGAPDH* (4310884E).

### Reverse transcription PCR

RT-PCR was performed using primers designed to specifically target murine *Usp5* exon 15: USP5_Exon15_fw: CATTGAGATGCCAGAGGAGCTCG and USP5_Exon15_rv: CATCCATGTGTGACATGACCCAG. For human myocardial tissue samples, RT-PCR was conducted using primers specific to human USP5 exon 15: USP5_Exon15_fw: ATCGAGATGCCAGAGGAGCTCG and USP5_Exon15_rv: GTGTGACATGACCCAGTTCATGGCG.

### Cultivation, transfection, and immunoprecipitation of HEK293 cells

HEK293 cells were cultured in Dulbecco′s modified Eagle′s medium containing supplements and transiently cotransfected with PSMD14-GFP and a V5-tagged USP library, or with PSMD15-V5 and either hs*USP5*^long^-GFP or hs*USP5*^short^-GFP, using TurboFect following the manufacturer’s protocol. After 24 hours, cells were lysed in radioimmunoprecipitation assay (RIPA) buffer with protease and phosphatase inhibitors, briefly sonicated, and centrifuged at 14,000*g* for 20 min. Protein concentration was measured using RotiQuant Universal. For immunoprecipitation, cell lysate (1 mg ml^−1^) was incubated with 40 μg of anti-V5 agarose beads at 4°C for 90 min. Protein complexes were washed with 1× phosphate-buffered saline (PBS), and V5-tagged USPs were eluted by heating agarose beads in sample buffer [2× NuPAGE lithium dodecyl sulfate (LDS)] at 95°C for 5 min.

hs*USP5* variants tagged with GFP were precipitated by incubation of cell lysate (1 mg ml^−1^) with 5 μg of anti-GFP or immunoglobulin G (IgG) antibody for 75 min at 4°C with rotation. This was followed by an additional 60-min incubation with 40 μg of protein G–Sepharose beads at 4°C on rotation. After washing in RIPA buffer and 50 mM tris-HCl, (pH 7.6), elution of GFP-tagged hs*USP5* variants was achieved by boiling the beads in 2× NuPAGE LDS sample buffer at 95°C for 3 min. The precipitated proteins were loaded onto SDS-PAGE (polyacrylamide gel electrophoresis) and subsequently analyzed by immunoblotting.

### Plasmids

V5-tagged hsUSP5 plasmids were obtained from the Center for Cancer Systems Biology Human ORFeome8.1 V8.1 Library (GE Healthcare Dharmacon). PSMD14-GFP and PSMD14-V5 constructs were created by PCR amplification of mouse Psmd14 open reading frame (ORF), followed by three-piece ligation into a modified pPyCAG-IP backbone vector. GFP-hsUSP5 was PCR-amplified and ligated together with the modified pPyCAG-IP vector. The HA-BioID fusion plasmid was constructed by amplifying the human USP5 ORF (long isoform) and BirA*(R118G)-HA from the pcDNA3.1 MCS-BirA*(R118G)-HA vector (Addgene 36047, Kyle Roux Lab), followed by three-piece ligation into a modified pPyCAG-IP backbone. Similarly, the Myc-BioID fusion plasmid was generated by amplifying the USP5 ORF (long isoform) and Myc-BirA*(R118G) from the pcDNA3.1 mycBirA* vector (Addgene 35700), with final assembly by three-piece ligation into the modified pPyCAG-IP vector.

### Protein extraction, immunoblot analysis, and antibodies

Cells were lysed by homogenization in RIPA buffer, to which protease and phosphatase inhibitors had been added, followed by 20 s of sonication, and 30-min incubation at 4°C with rotation. The extraction buffer for sonication of human myocardial samples for 20 s contained protease and phosphatase inhibitors. Homogenates were clarified by centrifugation at 14,000 rpm for 20 min at 4°C, and protein concentration was determined. Proteins (20 to 30 μg) were subjected to SDS-PAGE and transferred onto nitrocellulose membranes. Proteins bound by antibodies were detected via chemiluminescence by the SuperSignal West Pico Chemiluminescent Substrate kit or the WesternBright Chemiluminescence Substrate Sirius. Multiple antibodies were used to probe a single blot, allowing the repeated use of a single loading control as a reference for different reactions. Such loading controls are therefore shown several times, together with different antibody reactions. In Fig. 1 (E and F), the following proteins in the input were detected on a single membrane: DES (52 kDa), PSMA4 (29 kDa), PSMD14 (35 kDa), and RALA (26 kDa) as loading control; USP5 (96 kDa), PSMC4 (47 kDa), and RALA (26 kDa) as loading control; PSMC3 (49 kDa), PSMD12 (53 kDa), and RALA as loading control (26 kDa). In Fig. 2 (I and K), the same membrane was reprobed with antibodies against USP5 (96 kDa) and GAPDH (36 kDa) as loading control after probing for ubiquitin (multiple sizes). Quantification was accomplished using the ChemiDoc MP Imaging System. Densitometry analyses of immunoblots were performed using ImageJ software.

Antibodies were used at the indicated dilutions as follows: rabbit anti-cleaved caspase-3 (Asp^175^) (5A1E) (Cell Signaling Technology; 9664), rabbit anti-desmin (Sigma-Aldrich; D8281), mouse-anti FLAG, clone M2 (Sigma-Aldrich; F3165), rabbit anti-GAPDH (Cell Signaling Technology; 2118), rabbit anti-GFP (Abcam; ab6556), mouse anti-GFP (Roche/Merck; 11814460001), goat anti-GFP (custom-made), rabbit anti-LC3A/B=LC3I/II (D3U4C) (Cell Signaling Technology; 12741), rabbit anti-HDAC6 (Abnova; PAB18362), rabbit anti-NBR1 (Novus Biologicals; NBP1-71703SS), rabbit anti-PSMA4 (Abcam; ab191403), mouse anti-PSMA7 (Enzo Life Sciences; BML-PW9140-0100), rabbit anti-PSMB1 (Atlas Antibodies; HPA029635), rabbit anti-PSMB2 (Sigma-Aldrich; HPA026322), rabbit anti-PSMC3 (Sigma-Aldrich; HPA006065), rabbit anti-PSMC4 (OriGene; TA332530), rabbit anti-PSMD4 (Cell Signaling Technology; 12441), rabbit anti-PSMD11 (Novus Biologicals; NBP1-46192), rabbit anti-PSMD12 (Biorbyt; orb214470), rabbit anti-PSMD14 (Abcam; ab109123), mouse anti-SQSTM1/p62 (Abcam; ab56416), mouse anti–α-tubulin (Sigma-Aldrich; T6074), rabbit anti-USP5 (Bethyl Laboratories; A301-542A), or rabbit anti–V5-tag (Abcam; ab9116).

### BioID analysis

BioID fusion proteins [Myc-BirA*(R118G)-hsUSP5 and hsUSP5-BirA*(R118G)-HA, ~132 kDa] and their BirA* controls (~37 kDa) were transfected into HEK293 cells using TurboFect, following the manufacturer’s instructions. After 24 hours, cells were treated with 50 μM biotin and incubated for 18 hours. Cells were then lysed, and biotinylated proteins were captured on streptavidin-coated magnetic beads (*30*). Elution was performed by heating beads in NuPAGE LDS buffer at 98°C for 10 min. Proteins were separated by SDS-PAGE, digested in-gel, and analyzed by liquid chromatography–MS/MS (LC-MS/MS), with protein identification based on the UniProt human database. BioID analysis was conducted in technical triplicates, and GO enrichment was assessed with GOrilla. Comparisons were made between BirA*-USP5 and BirA* alone; HA tags on C-terminal fusions and Myc tags on N-terminal fusions enabled differentiation by Western blotting.

### Cultivation, siRNA knockdown, and immunoprecipitation of HL-1 CMs

HL-1 CMs (*31*) (SCC065, purchased from Merck) were cultured as reported by manufacturer instructions under 5% CO_2_ atmosphere and used for up to 10 passages after initial thawing. siRNA knockdown was performed using control siRNA (ON-TARGETplus Non-targeting Control Pool, D-001810-10-05, Dharmacon) and USP5 siRNA (ON-TARGETplus siRNA against murine USP5; L-046492-01-0005, Dharmacon) with Lipofectamine RNAiMAX (13778075, Thermo Fisher Scientific) according to the manufacturer’s protocol. Immunoprecipitations were performed with the Pierce Classic Magnetic IP/Co-IP Kit (88804, Thermo Fisher Scientific) according to the manufacturer, using anti-USP5 (A301-542M, Bethyl) or IgG. Beads were boiled in 2× NuPAGE LDS sample buffer at 95°C for 10 min for elution of proteins.

### Microarray and GO term analysis

Quality assessment of CM RNA was analyzed by the RNA 6000 Nano Kit on an Agilent 2100 Bioanalyzer. Labeling of RNA followed the Affymetrix Whole Transcript Sense Target Labeling protocol. RNA was hybridized using Affymetrix Clariom D assays. Data processing and analysis were conducted using the RMA algorithm in the Affymetrix Expression Console. Statistical significance of differentially expressed transcripts was determined using a Student’s *t* test on log_2_-transformed data, with fold-change (FC) values calculated using DNAStar Arraystar 11 (*74*). A total of 229 transcripts were identified with FC ≥ 1.5 or FC ≤ 0.75 (*P* ≤ 0.05). GO annotations for the identified gene sets were analyzed using GOrilla (http://cbl-gorilla.cs.technion.ac.il/).

### Whole-proteome analysis combined with LC-MS/MS

Proteins (40 μg) of isolated CMs were separated by SDS-PAGE, stained with InstantBlue Protein Stain, and sliced into uniform pieces for in-gel digestion as previously described (*75*). Purification of extracted peptides was accomplished via C-18 stop and go extraction using in-house manufactured stage tips (*76*) and analyzed by LC-MS/MS using an LC system coupled to an LTQ-Orbitrap Velos mass spectrometer equipped with a nano-electrospray ionization source. Peptide separation was assessed on a linear gradient for 130 min using a binary buffer system. A resolution of 70,000 at 200 mass/charge ratio (*m*/*z*) was used for acquisition of MS spectra in the Orbitrap. Raw data analysis was performed using MaxQuant in combination with the implemented Andromega search engine. Correlation of obtained spectra with the UniProt murine database allowed assignment of proteins. Searches were performed with tryptic specificity and default settings for missed cleavages and mass tolerance for MS and MS/MS datasets. Carbamidomethylation at cysteine residues of proteins was set as a fixed modification, and oxidation of methionine and acetylation at the N terminus were set as variable modifications. The minimal peptide length was defined as seven amino acids, and the false discovery rate at protein and peptide level was set to 1%. *P* values ≤ 0.05 were considered statistically significant.

### Immunofluorescence staining

Cells were cultured on coverslips, transduced with the respective adenovirus, and fixed for 15 min in 4% paraformaldehyde. Permeabilization was done with 0.1% Triton X-100 for 10 min. Mouse and human cardiac tissue sections were only permeabilized in 0.1% Triton X-100 for 10 min. Nonspecific binding was blocked with 2% fetal calf serum and 2% bovine serum albumin in PBS for 30 min. Samples were incubated with primary antibodies at 4°C overnight, followed by incubation with secondary antibody conjugated with Alexa Fluor 488, 594, 633, or 647 (Thermo Fisher Scientific) for 2 hours. Counterstaining of nuclei was done with 4′,6-diamidino-2-phenylindole (DAPI; Invitrogen) for 10 to 15 min. Immunostaining of cells and mouse cardiac tissues was carried out from littermates and processed under the same conditions. Primary antibodies were used as follows: rabbit anti-USP5 (Bethyl Laboratories, A301-543A), phalloidin–fluorescein isothiocyanate (FITC) (Sigma-Aldrich, P5282), phalloidin–tetramethyl rhodamine isothiocyanate (TRITC) (Sigma-Aldrich, P1951), mouse anti-ubiquitin (P4D1) (Cell Signaling Technology, 3936), and mouse anti-SQSTM1/p62 (Abcam, ab56416).

### Immunoblot analysis to detect ubiquitin

For Western blot analysis of ubiquitin, nitrocellulose membranes were boiled for 30 min in double-distilled H_2_O and blocked with 0.5% gelatin in PBS with 0.05% Tween 20. Membranes were incubated with mouse anti-ubiquitin antibody (P4D1) (Cell Signaling Technology; 3936) or rabbit anti-ubiquitin K48-specific antibody (clone Apu2, Millipore; 05-1307) at 4°C overnight. For the rabbit anti-ubiquitin K63-specific antibody (clone Apu3, Millipore; 05-1308), membranes were incubated for 1 hour at room temperature, followed by 2 hours at room temperature with HRP-conjugated secondary antibodies.

### Isolation and transduction of MEFs and adult hepatocytes

MEFs were harvested from E13.5 embryos as described (*77*) and maintained in supplemented Dulbecco’s modified Eagle’s medium (DMEM). Adult hepatocytes were isolated as previously published (*78*) with slight modifications and maintained in supplemented M199 cell culture medium. *Usp5* inactivation was accomplished through adenoviral transduction using an engineered adenovirus, expressing codon-optimized Cre recombinase, driven by the cytomegalovirus (CMV) promoter (Ad-Cre), for 6 hours. A type 5 adenovirus with E1/E3 deletion (Ad-Null) served as a mock control. Adenoviral infected cells were analyzed 6 days after infection.

### Proteasome activity assay: GLO assay

Activities of CT-L, T-L, and C-L were measured in CMs isolated from either WT or *Usp5*-KO mice using Proteasome-Glo Assays (Promega: G8622 for CT-L, G8632 for T-L, and G8642 for C-L). In brief, cells were lysed using 0.2% NP-40–supplied TSDG buffer (10 mM tris-HCl, 1.1 mM MgCl_2_, 10 mM NaCl, 0.1 mM EDTA, 1 mM NaN_3_, 1 mM dithiothreitol, 2 mM adenosine triphosphate (ATP), 10% glycerol) on ice for 20 min. Subsequently, cells were centrifuged at 4°C with 13,000 rpm for 20 min to extract proteins. Native lysate (1 μg) was diluted in 20 μl of nuclease-free water and subsequently mixed with 20 μl of substrate at a white-bottom 96-well plate. Luminescence was measured after 60-min incubation using the GloMax-Multi plate reader according to the manufacturer’s instructions (Promega). Equal volumes of nuclease-free water were used to determine the background. Of note, the same samples were used for the in-gel proteasome assay.

### In-gel proteasome assay

The detailed method for detecting in-gel proteasome activity was reported previously (*34*). For native PAGE, 25 μg of native protein lysate was loaded into gel (NuPAGE 3 to 8%, Tris-Acetate Mini Protein Gels, Thermo Fisher Scientific) pockets. Empty pockets were filled with an equal volume of loading buffer. After completion of electrophoresis, transfer of proteins to a polyvinylidene difluoride membrane was done by standard wet blotting, following protein solubilization. Membranes were blocked at room temperature for an hour and then incubated with a primary antibody overnight at 4°C: proteasome 20*S* α1, 2, 3, 5, 6, and 7 subunit monoclonal antibody (MCP231), BML-PW8195-0100, Enzo Life Science, 1:1500 dilution. Membrane development was conducted using ECL substrates (SuperSignal West Pico PLUS Chemiluminescent Substrate, 34579, Thermo Fisher Scientific) the next day after secondary antibody [anti-mouse IgG, horseradish peroxidase (HRP)–linked antibody, #7076S, Cell Signaling Technology, 1:25,000 dilution] binding and proper washing procedures. The specific activity was calculated by dividing the relative CT-L activity by the relative protein levels, which were determined from the same gel. Notably, the identical samples were used for the GLO assay.

### Proteasome activity assay: AMC assay

Proteasome activity was analyzed as previously described (*79*). In brief, cells were harvested and lysed in ice-cold proteasome activity assay buffer, subjected to sonication, and clarified by centrifugation at 13,000*g* for 15 min at 4°C. The lysate (15 to 25 μg) was transferred to a black 96-well plate, and 40 μM of specific activity–based fluorogenic peptides, diluted in proteasome assay buffer, was added. Samples were incubated for 30 min at 37°C, and the fluorescence intensity was determined using a Mithras LB 940 Multimode Multiplate Reader (380-nm excitation and 460-nm emission). CT-L activity was determined using the substrate Suc-Leu-Leu-Val-Tyr-AMC (7-amino-4-methylcoumarin) (BML-P802, Enzo).

### Detection of protein aggregates

Protein aggregates were visualized using the ProteoStat Aggresome Detection Kit following the guidelines of the manufacturer (*80*). The kit contains a 488/594-nm excitable red fluorescent molecular rotor dye, which selectively binds to denatured proteins in fixed and permeabilized cells. Binding triggers a reaction, which makes protein aggregates brightly fluorescent.

### Pharmacological treatment

Cells were treated with 10 μM MG132, 20 μM chloroquine, 100 nM bafilomycin A1, or 0.2% DMSO as a mock control for 4 hours.

### Cardiac magnetic resonance imaging

Cardiac magnetic resonance imaging (MRI) was done using a 7.05 T Bruker PharmaScan, with a 760 mT m^−1^ gradient system and a cryogenically cooled receiver coil (CryoProbe, Bruker) with a four-channel–phased array element 1H in combination with a 72-mm room temperature volume resonator for transmission and self-gated cardiac imaging (Intragate, Bruker) (*81*). MRI was performed by the gradient echo method (repetition time: 6.3 ms; echo time: 1.6 ms; field of view: 2.20 × 2.20 cm; slice thickness: 1.0 mm; matrix: 128 × 128; repetitions: 100), while animals were kept under isoflurane anesthesia (1.5% to 2.0%). Scout images showing two- and four-chamber views of the heart were used to localize the imaging plane. Images were acquired in short-axis view, orthogonal to the septum in both scout images. Seven to 10 slices were acquired from multiple contiguous short-axis slices to completely cover the left ventricle. Qmass digital imaging software (Medis, Leiden, The Netherlands) was used for analysis of cardiac parameters.

### Morphological analysis

Dissected mouse hearts were fixed in 4% paraformaldehyde at 4°C overnight. Next, the samples were kept in 30% sucrose at 4°C for 24 hours and were subsequently embedded in FSC 22 Frozen Section Compound for further storage at −80°C. Cross sections of hearts at the middle of the ventricle were obtained at a thickness of 6 μm using a CM1950 cryostat manufactured by Leica Biosystems, and placed onto SuperFrost Plus slides. Heart morphology was analyzed after hematoxylin and eosin (H&E; hematoxylin: Roth T865.2; eosin: Roth X883.2) or Masson’s trichrome (Sigma-Aldrich; HT15) staining according to routine procedures.

### Electron microscopy

Before removal of hearts, animals were anaesthetized and subsequently perfused with 1.5% glutaraldehyde and 1.5% paraformaldehyde in 0.15 M Hepes (pH 8.0). Dissected hearts were stored at 4°C in the same fixative for at least 24 hours. Afterward, heart samples were postfixed in 1% osmium. Before epoxy resin embedding, hearts were immersed en bloc in 50% saturated watery uranyl acetate, dehydrated by an ascending ethanol series, and embedded in Agar 100. An ultramicrotome was used to generate ultrathin sections. Images were acquired using a Zeiss EM902 transmission electron microscope. Digital images were recorded using a slow scan 2K CCD (charge-coupled device) camera (TRS, Tröndle, Moorenweis, Germany).

### Nonfailing and failing human heart muscle tissues

Human samples for immunofluorescent stainings were provided by the Kerckhoff Hospital, Bad Nauheim, Germany. All patients provided written informed consent for the research use of heart tissue, in accordance with the Medical Council’s requirements and the hospital’s ethics guidelines. The study was approved by the ethics committee of the Medical Council in Hessen (“Ethik-Kommission der Landesärztekammer Hessen”), Germany (FF 8/2011). Additional human samples for Western blot analysis were provided by the Heart and Diabetes Center NRW, Bad Oeynhausen, Germany. Biobanking of myocardial specimen received approval by the local ethics committee of the Ruhr-University Bochum, Germany (Reg.-No. 21/2013). Tissue specimens were dissected from the left ventricles of end-stage heart failure patients (ejection fraction < 30%), diagnosed with DCM. Myocardial samples from individuals with preserved cardiac function (ejection fraction ≥ 65%) served as controls. Control samples did not present signs of myocardial damage or myocardial dilatation. Following dissection, heart tissue samples were immediately shock-frozen in liquid nitrogen and stored at −80°C until further use.

### Transverse aortic constriction

Anesthesia was induced with 4.5% isoflurane and 100% oxygen for about 1 min. After endotracheal intubation with a 20-gauge catheter, anesthesia was maintained with 1.5% isoflurane using a MiniVent (220 strokes/min, 230-μl SV). The anesthetized animal was positioned laterally on a heated surgical table (37°C), and eye ointment was applied to prevent dryness. The “closed-chest” model (*82*) was used with access via the second left intercostal space. Bupivacaine (1 mg/kg) was injected before opening the intercostal space to prevent postoperative pain. The aortic arch was exposed, and a stenosis was created using a 26- or 27-gauge needle based on the animal’s weight, with nonabsorbable 6/0 Perma-Hand silk sutures: A 27-gauge needle was used as a spacer for TAC ligation for mice weighing between 19 and 25 g, and a 26-gauge needle for mice weighing more than 25 g (*82*). The wound was closed with absorbable 5/0 Vicryl sutures. Ventilation was changed to pure oxygen until the animal breathed independently. Postoperative pain management included subcutaneous administration of buprenorphine (0.1 mg/kg) and metamizole in drinking water for 5 days, starting the day before surgery.

### Adenoviral infection of adult CMs with mini-titins

Adenoviral constructs expressing fragments of titin (“mini-titin”) containing the A150 fibronectin III domain Fn3-119 (WT titin, C31712R, P30091L, or R32450W mutant titin variant) (*47*, *83*), which is linked to the A-band Fn3 A170 (Fn3-170), and subsequent titin kinase domain tagged with GFP at the N terminus were made using the Virapower expression system from Invitrogen. In addition, adenoviruses with GFP-tagged DCM-linked titin missense variants (p.Val22232Glu and p.Gly27849Val) were constructed and packaged by VectorBuilder. The corresponding vector IDs are VB240415-1505xkc, VB240415-1508dkm, VB240415-1514eee, and VB240415-1519rgk, which can be used to retrieve detailed information about the vector on https://en.vectorbuilder.com/. Isolated CMs of *XMLC2-cre* mice (Cre) or *USP5*-overexpressing mice (cOE) were infected with the respective adenovirus overnight and analyzed 48 hours after infection.

### Image acquisition and processing

Cardiac tissue sections and stained cells were examined with a fluorescence microscope (Keyence, BZ-9000) or a confocal microscope (Leica SP8). To assure comparability of the immunofluorescence signals, all laser excitation settings were kept constant among different groups. BZ-II Analyzer, ImageJ/Fiji, and GIMP (version 2.8) software were used for image acquisition and processing. Protein aggregates were either manually quantified or analyzed using the AggreCount macro (*84*) in ImageJ/Fiji. Figure 10 was created by using BioRender software (agreement number VK27H66LCW).

### Statistical analysis

All data are shown as mean ± SD. No statistical method was used to predetermine samples size. Randomization or blinding was not applied for analyses. To determine the statistical significance of differences between two groups, Welch’s unequal variances *t* test or Wilcoxon rank sum test (Mann-Whitney *U* test) was used. For multiple comparison tests, one-way analysis of variance (ANOVA) and subsequent Holm-Sidak’s multiple comparison test, Kruskal-Wallis test and subsequent Dunn’s, or Tukey’s multiple comparisons were used. The Kaplan-Meier log-rank (Mentel-Cox) test was applied for analysis of survival curves. TAC experiments were evaluated using mixed-effects analysis with Tukey’s multiple comparison test, due to some missing values from the MRI measurements. No animals were excluded from statistical analysis. All statistical calculations were performed using GraphPad Prism Software, versions 7, 8, and 9. The following values were considered to be statistically significant: *P ≤ 0.05, **P ≤ 0.01, ***P ≤ 0.001, and ****P ≤ 0.0001.

## Supplementary Materials

### The PDF file includes:

Figs. S1 to S7 Legends for tables S1 to S3 Images of original uncropped Western blots
