# Supplementary material for: The deubiquitinase USP5 prevents accumulation of protein aggregates in cardiomyocytes
Source: Sci Adv. 2025 Jan 22;11(4):eado3852. doi: 10.1126/sciadv.ado3852 (PMC11753375; doi:10.1126/sciadv.ado3852)

Science Advances
AAAS

Supplementary Materials for

The deubiquitinase USP5 prevents accumulation of protein aggregates in cardiomyocytes

Yvonne Eibach *et al.*

Corresponding author: Thomas Braun, thomas.braun@mpi-bn.mpg.de

Sci. Adv. **11**, eado3852 (2025)
DOI: 10.1126/sciadv.ado3852

The PDF file includes:

Figs. S1 to S7
Legends for tables S1 to S3
Images of original uncropped Western blots

Other Supplementary Material for this manuscript includes the following:

Tables S1 to S3

Supplementary Figures

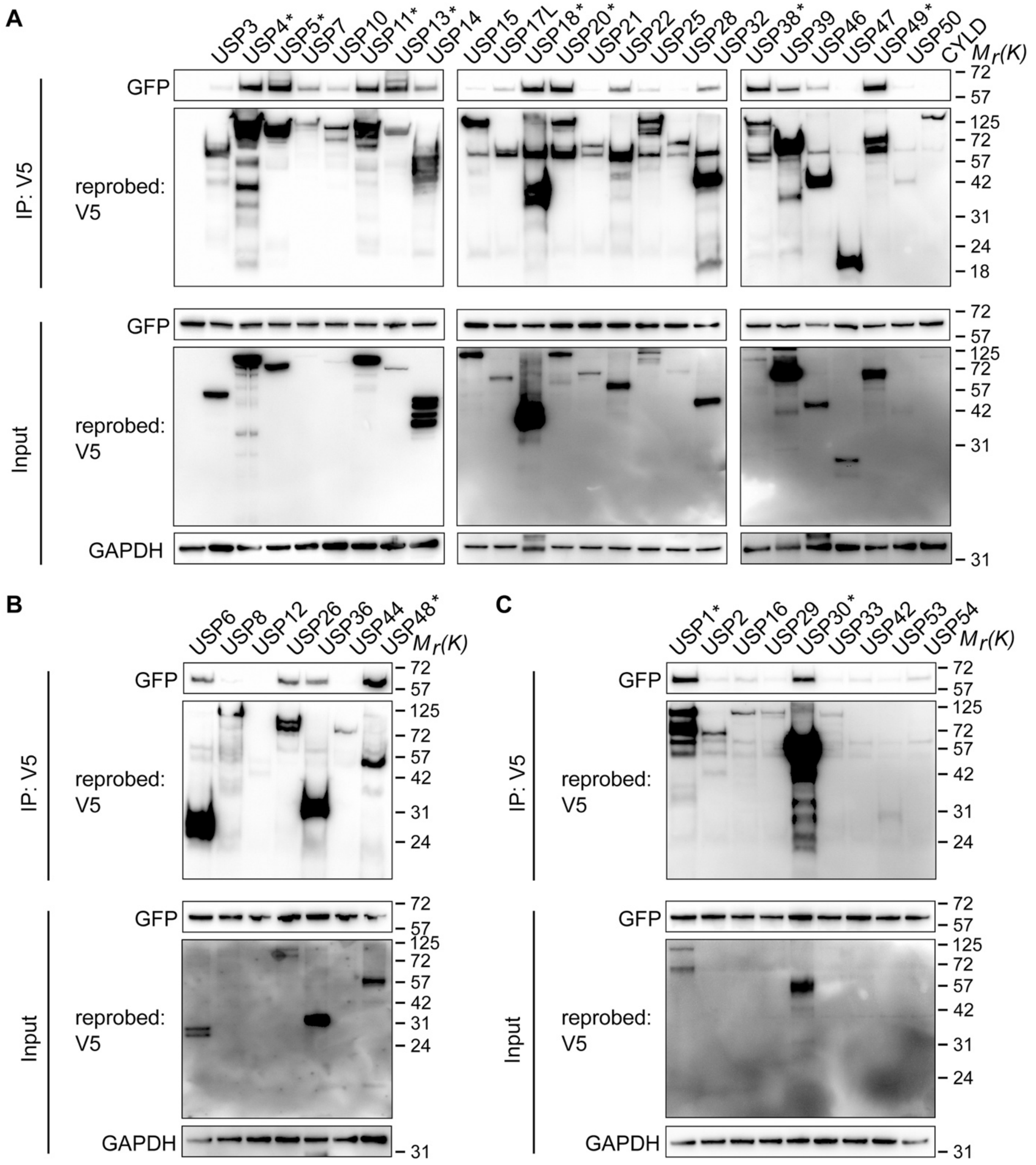

Fig. S1. Identification of DUBs interacting with PSMD14. Co-immunoprecipitation (IP) of GFP-tagged mouse PSMD14 with V5-tagged human ubiquitin-specific proteases (USPs) in HEK293 cells. **A-C** Immunoblot analysis of anti-V5 precipitated cell lysates (re)probed with anti-GFP and anti-V5 antibodies. Input and IP fractions are shown. *Represents DUBs/USPs including USP5 co-immunoprecipitated with PSMD14. GAPDH was used as a loading control.

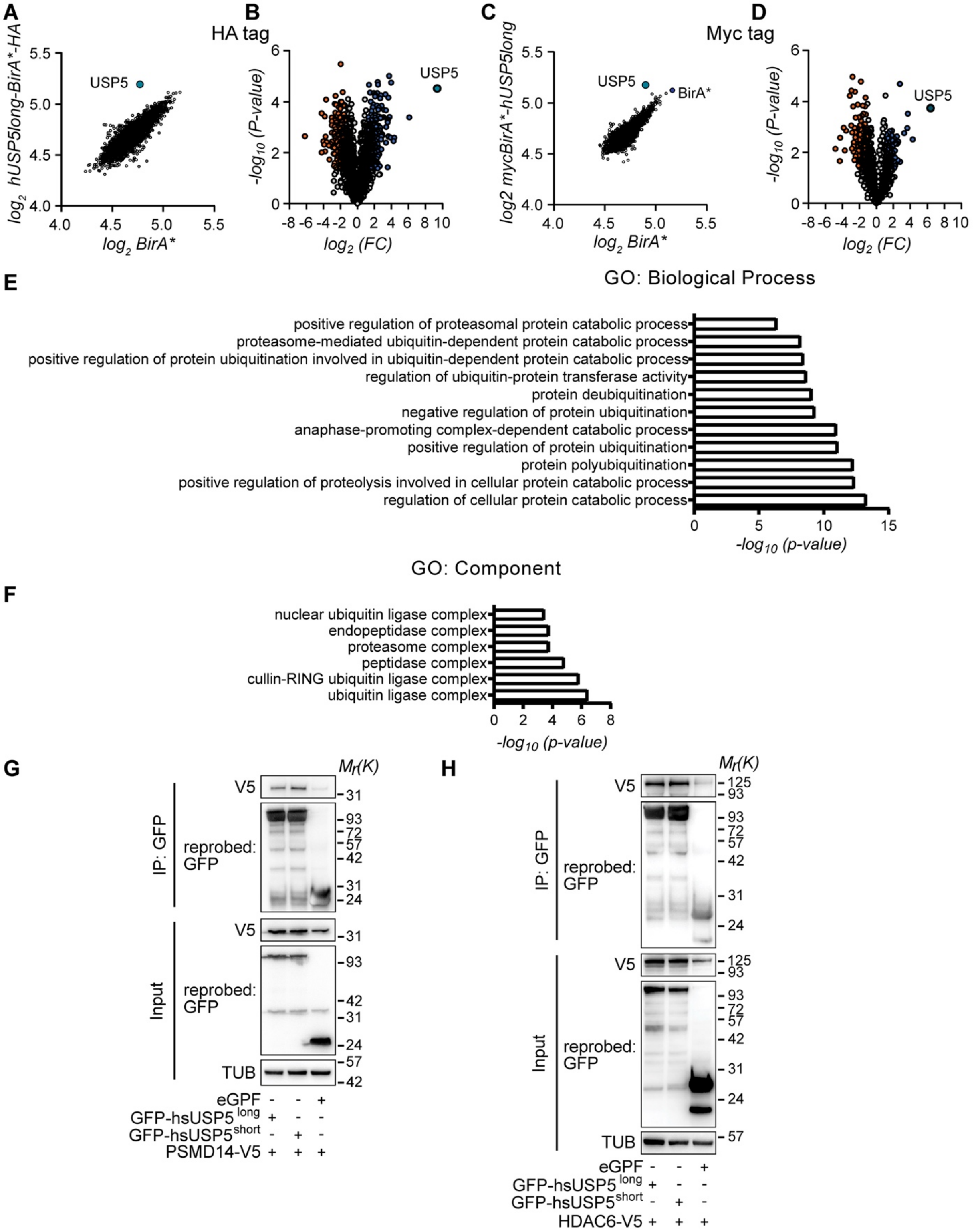

Fig. S2. Identification of USP5 interaction partners by BioID analysis and coIP in HEK293 cells. A, C Scatter plots of differently biotinylated proteins identified in triplicate experiments in BirA*-HA and USP5-BirA*-HA (A) or Myc-BirA* and Myc-BirA*-USP5 (C) transduced cells.

B, D Volcano plots of differentially biotinylated proteins in cells transduced with the BirA*-HA and USP5-BirA*-HA (B) or Myc-BirA* and Myc-BirA*-USP5 (D) fusion protein, respectively. Proteins biotinylated by USP5-BirA* are depicted in blue and proteins labeled by BirA* are depicted in orange. Grey indicates non-significantly changed proteins. **E, F** Enriched GO terms of proteins biotinylated by USP5-BirA* with respect to biological processes (E) and cellular components (F) as based on - log_{10} *P-values*. **G, H** Immunoblot analysis of anti-GFP precipitated cell lysates (re)probed with anti-V5 and anti-GFP antibodies, revealing the interaction of GFP-tagged hsUSP5 isoforms with V5-tagged PSMD14 (G) and V5-tagged HDAC6 (H). Tubulin (TUB) was used as a loading control.

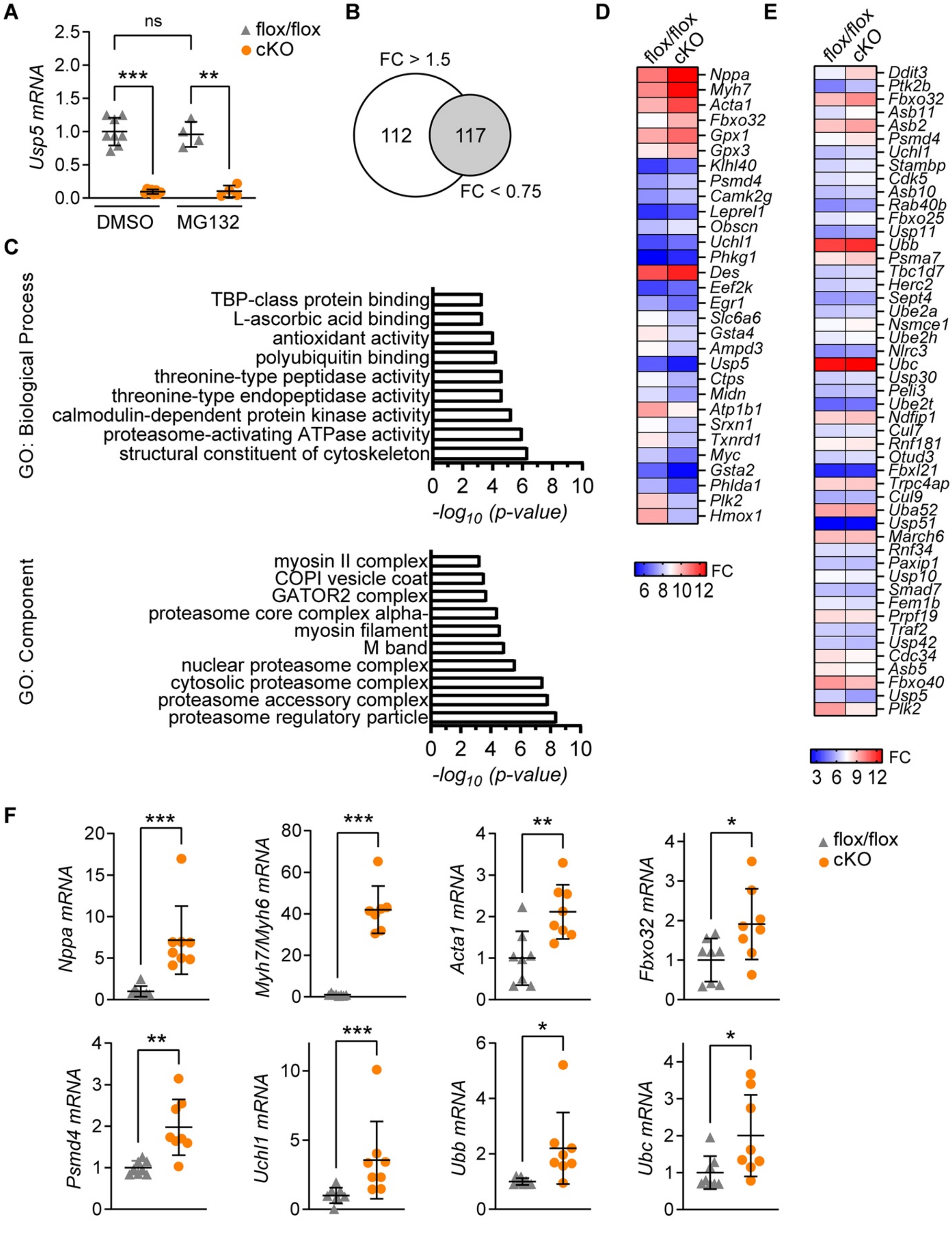

Fig. S3. Transcriptional profiling of *Usp5*-deficient cardiomyocytes. A qRT-PCR of *Usp5* mRNA expression in flox/flox and cKO cardiomyocytes treated with (n=4) or without MG132

(n=8). Values are normalized to *Gapdh*. Welch's ANOVA test. **B** Venn diagram showing the number of differentially expressed genes in USP5-deficient cardiomyocytes compared to flox/flox cardiomyocytes scored by microarray analysis (n=3). Significantly up-regulated genes are shown in white (FC > 1.5, $P < 0.05$), significantly down-regulated genes in grey (FC < 0.75, $P < 0.05$). FC: fold change. **C** GO term analysis of differentially expressed genes based on $-log_{10}$ *P-values.* GO terms refer to biological processes (top) and cellular components (bottom). **D, E** Heat map showing differentially expressed, cardiac stress response-related (D) and ubiquitin-related (E) transcripts in USP5-deficient cardiomyocytes. **F** qRT-PCR validation of *Nppa, Myh7/Myh6, Acta1, Fbxo32, Psmd4,* and *Uchl1* as well as polyubiquitin precursor genes *Ubb* and *Ubc* mRNA expression in flox/flox and cKO cardiomyocytes (n=7-8). Values were normalized to *Gapdh*. Welch's unequal variances *t*-test. *P ≤ 0.05; **P ≤ 0.01; ***P ≤ 0.001.

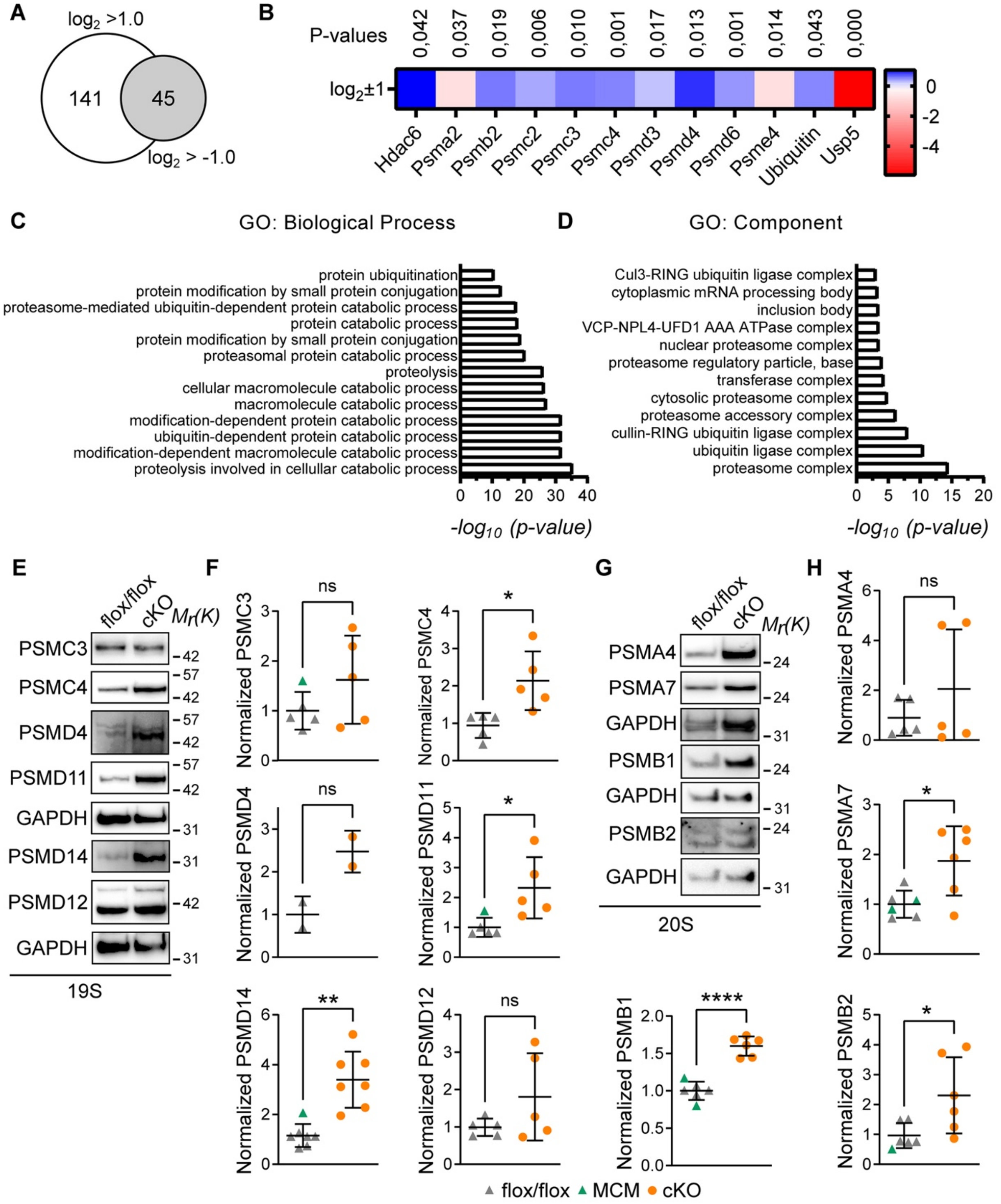

Fig. S4. Proteomic profiling of *Usp5*-deficient cardiomyocytes. A Venn diagram showing the number of differentially expressed proteins in USP5-deficient cardiomyocytes determined via whole proteome analysis. Significantly up-regulated proteins are shown in white ($log_2 > 1.0$, $P \leq 0.05$), significantly down-regulated proteins in grey ($log_2 > -1.0$, $P \leq 0.05$). flox/flox n=3, cKO n=4. **B** Heat map showing significantly deregulated, selected ubiquitin-related proteins (selected

from the GO Term "Ubiquitin") in USP5-deficient cardiomyocytes in alphabetical order. **C, D** GO term analysis of differentially expressed proteins based on *-log_{10} P-values* of LQF intensities. GO terms refer to biological process (C) and cellular components (D). **E, F** Immunoblot analysis (E) and quantification (F) of 19S proteasome subunits in flox/flox, MCM and cKO cardiomyocytes (n=2-7). The lower band in the respective image corresponds to PSMD4 (49 kDA). **G, H** Immunoblot analysis (G) and quantification (H) of indicated 20S proteasome subunits in flox/flox, MCM and cKO cardiomyocytes (n=5-6). The upper band in the respective image corresponds to PSMB2 (23 kDA). **E, G** GAPDH was used as loading control. Multiple antibodies were used to probe a single blot, allowing the use of a single loading control as a reference for different reactions. **F, H** Protein levels were normalized to GAPDH. Welch's unequal variances *t*-test. *P ≤ 0.05; **P ≤ 0.01; ****P ≤ 0.0001.

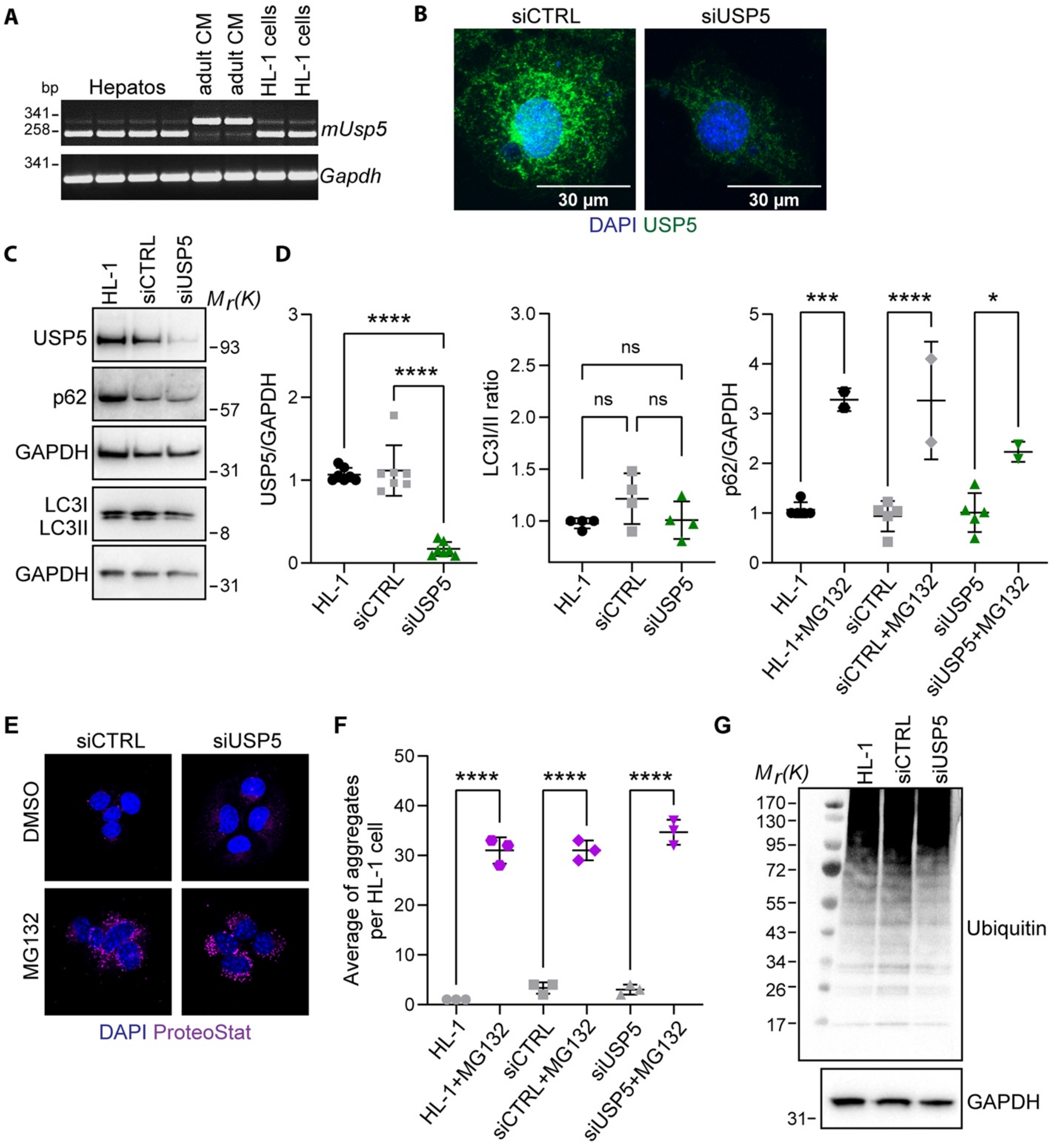

Fig. S5. Knockdown of *Usp5* in proliferating HL-1 cardiomyocytes is not affecting PQC. A Semi-quantitative RT-PCR showing long and short spliced variants of *mUsp5* in adult hepatocytes (hepatos, n=4), adult cardiomyocytes (CMs, n=2) and HL-1 cardiomyocytes (n=2). *Gapdh* was used as loading control. **B** Immunofluorescent staining for USP5 (green) and DAPI (blue) in HL-1 cardiomyocytes transfected with siCTRL or siUSP5. Scale bar: 30 µm. **C, D** Immunoblotting (C) and quantification (D) for USP5, LC3 and p62 protein levels in HL-1 cardiomyocytes without or with siCTRL or siUSP5 transfections. Cells were treated with MG132 as indicated (n=2-7). **E, F** Staining (E) and quantification (F) for protein aggregates using the ProteoStat assay (magenta)

and nuclei (DAPI, blue) in HL-1 cardiomyocytes transfected with siCTRL or siUSP5, and MG132 treatment as indicated (n=3). **G** Immunoblot for ubiquitin in USP5-deficient HL1 cardiomyocytes and respective controls. **C, G** GAPDH was used as loading control. **D, F** One-way ANOVA with Tukey's multiple comparison test. $^{*}P \leq 0.05$; $^{***}P \leq 0.001$, $^{****}P \leq 0.0001$.

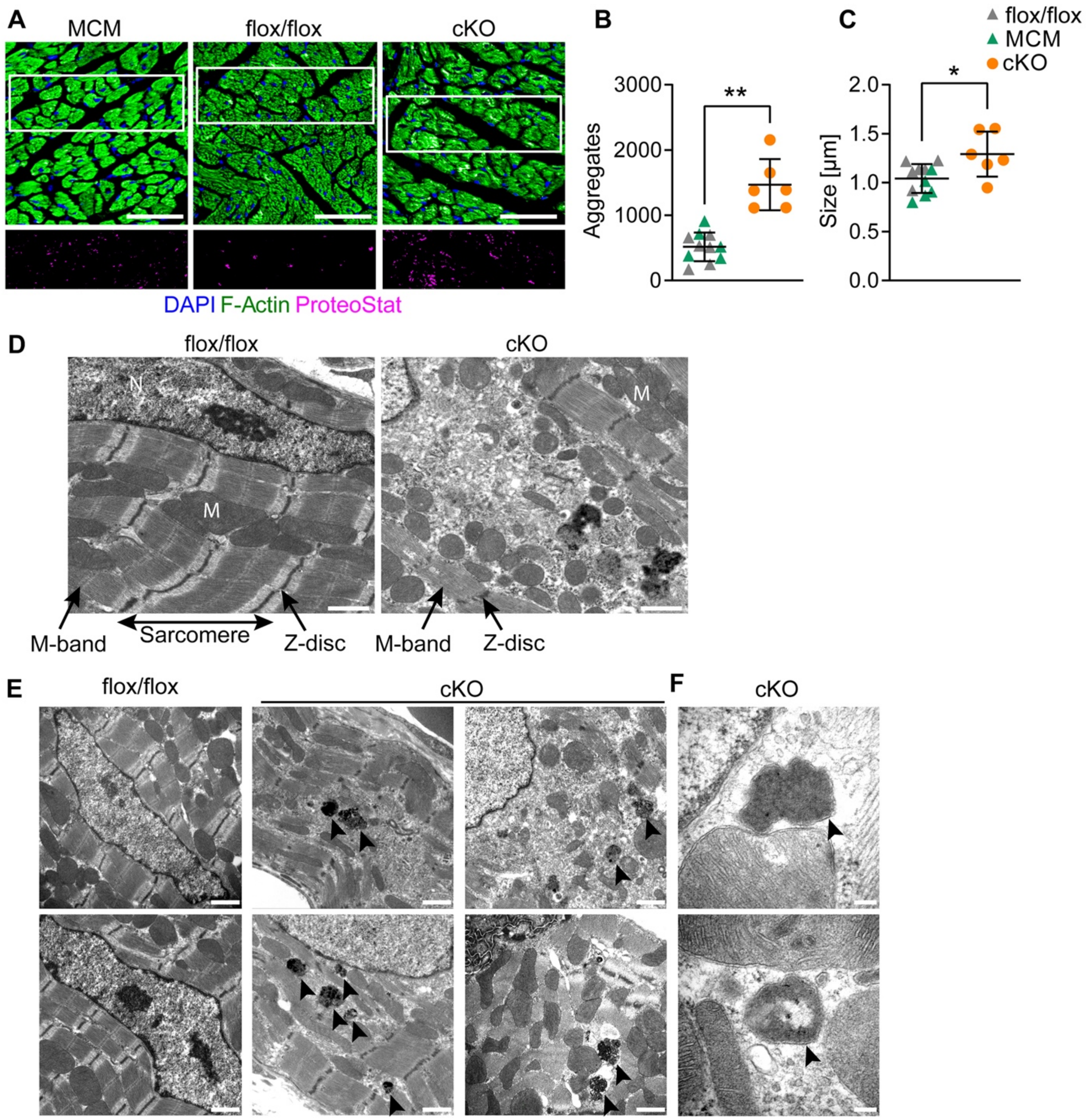

Fig. S6. Compromised PQC in *Usp5*-deficient cardiomyocytes increases protein aggregates. **A** Staining for protein aggregates (ProteoStat, magenta), F-Actin (green), and nuclei (DAPI, blue) of left ventricles from flox/flox, MCM, and cKO mice. Scale bar: 25 µm. **B, C** Number (B) and size (C) of protein aggregates in left ventricles from flox/flox (n=6), MCM (n=5), and cKO (n=6) mice. Welch's unequal variances t-test. **D** Ultrastructural images showing sarcomeres of left ventricles from flox/flox and cKO mice (n=3). Arrows in cKO mice indicate degenerating myofibrils. M: mitochondria. N: nucleus. Scale bar: 1000 nm. **E, F** Electron microcopy reveals more pronounced membrane-enclosed lysosomal structures (see arrowheads) in cKO compared to controls (n=3). **E** Scale bar: 1000 nm. **F** Scale bar: 100 nm. **D-F** The ultrastructure was analyzed 15 or 24 days after completion of the tamoxifen treatment. $^{*}P \leq 0.05$; $^{**}P \leq 0.01$.

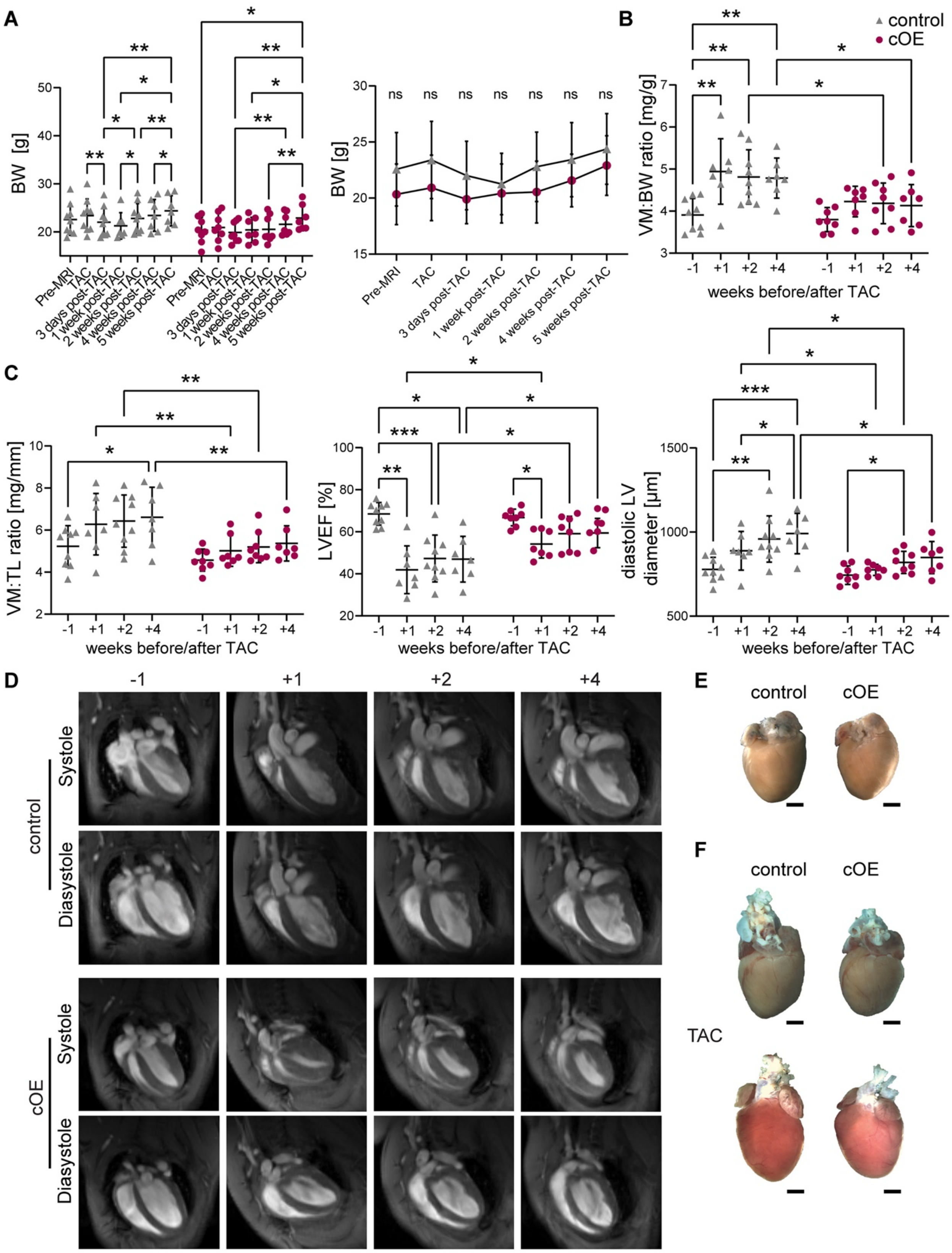
A
B
C
D
E
F
BW [g]
Pre-MRI
TAC
3 days post-TAC
1 week post-TAC
2 weeks post-TAC
4 weeks post-TAC
5 weeks post-TAC
ns
control
cOE
VM:BW ratio [mg/g]
weeks before/after TAC
VM:TL ratio [mg/mm]
LVEF [%]
diastolic LV diameter [µm]
-1
+1
+2
+4
Systole
Diasystole
TAC

Fig. S7. Overexpression of USP5 in the heart attenuates pressure overload-induced cardiac hypertrophy in mice. A Effect of cardiomyocyte-specific overexpression of *Usp5* on body weight (BW) at indicated time points before and after TAC surgery. **B** Ventricular mass (VM) to body weight (VM:BW) and ventricular mass to tibia length (VM:TL) ratios for cOE and control mice. **C** Quantification of the left ventricular ejection fraction (LVEF) and diastolic left ventricular (LV) diameter in cOE and control mice ($n \leq 9$). MRI-based TAC data were analyzed using mixed-effect analysis with Tukey's multiple comparison test. TAC surgeries were conducted with mice aged 14 ± 1 weeks. Control (n=9, 5 females, 4 males), cOE (n=8, 5 females, 3 males). **D** Long-axis views of hearts from control and cOE mice at end-systole and end-diastole after TAC surgery at indicated time points. **E, F** Representative images of hearts from control and cOE mice under physiological conditions (E) and 5 weeks after TAC surgery (F). Scale bar: 1000 µm. $^{*}P \leq 0.05$; $^{**}P \leq 0.01$; $^{***}P \leq 0.001$.

Table S1 Identification of USP5 interaction partners by BioID analysis in HEK293 cells (supplied as separate Excel tables). HEK293 cells were transiently transfected with BirA* fusion proteins: BirA*-USP5 to BirA* alone. BirA* is not conjugated to GFP. However, C-terminal fusions are HA-tagged (USP5-BirA*-HA und BirA*-HA) and N-Terminal fusions are Myc-tagged (Myc-BirA*-USP5 und Myc-BirA*). Biotinylated proteins were enriched using Streptavidin-beads and analyzed by LC-MS/MS. Raw data for the scatter plots (Supplementary Figure 2A, C) and volcano plots (Supplementary Figure 2B, D) are listed.

Table S2 Transcriptional profiling of *Usp5*-deficient cardiomyocytes. (supplied as separate Excel tables). Isolated cardiomyocytes of flox/flox (Ctrl) and cKO (mut) mice were processed for microarray and GO term analysis. List of up- and down-regulated genes. Genes listed in the GO term “Ubiquitin” are marked in an extra column.

Table S3 Shotgun mass spectrometry analysis of *Usp5*-deficient cardiomyocytes. (supplied as separate Excel tables). Isolated cardiomyocytes of flox/flox (Ctrl) and cKO (mut) mice were processed for whole proteome analysis combined with LC-MS/MS and GO term analysis. List of up- and down-regulated proteins. Proteins listed in the GO term “Ubiquitin” are marked in an extra register tab.

Images of original uncropped Western Blots for Figs. 1-7 and Figs. S1-S7

Figure 1E

ACTN2

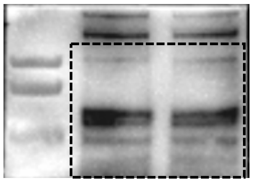

GAPDH

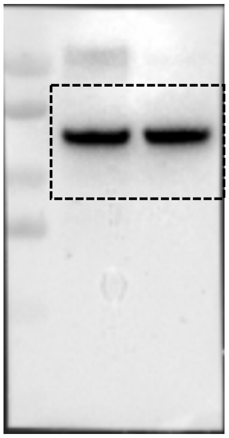

cTNT

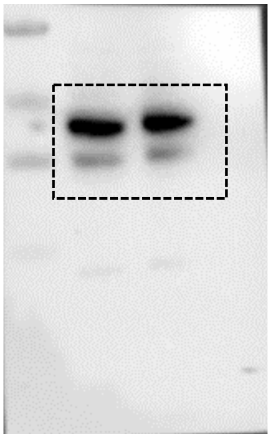

RALA

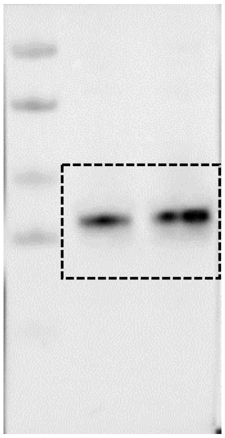

DESMIN

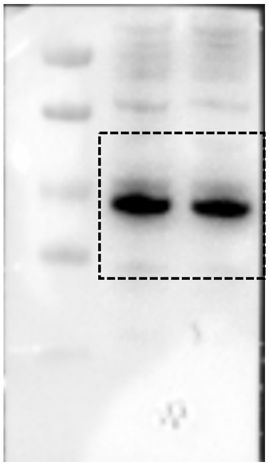

RALA

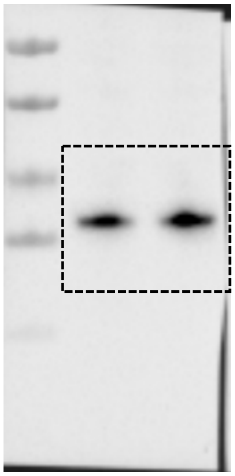

Figure 1F

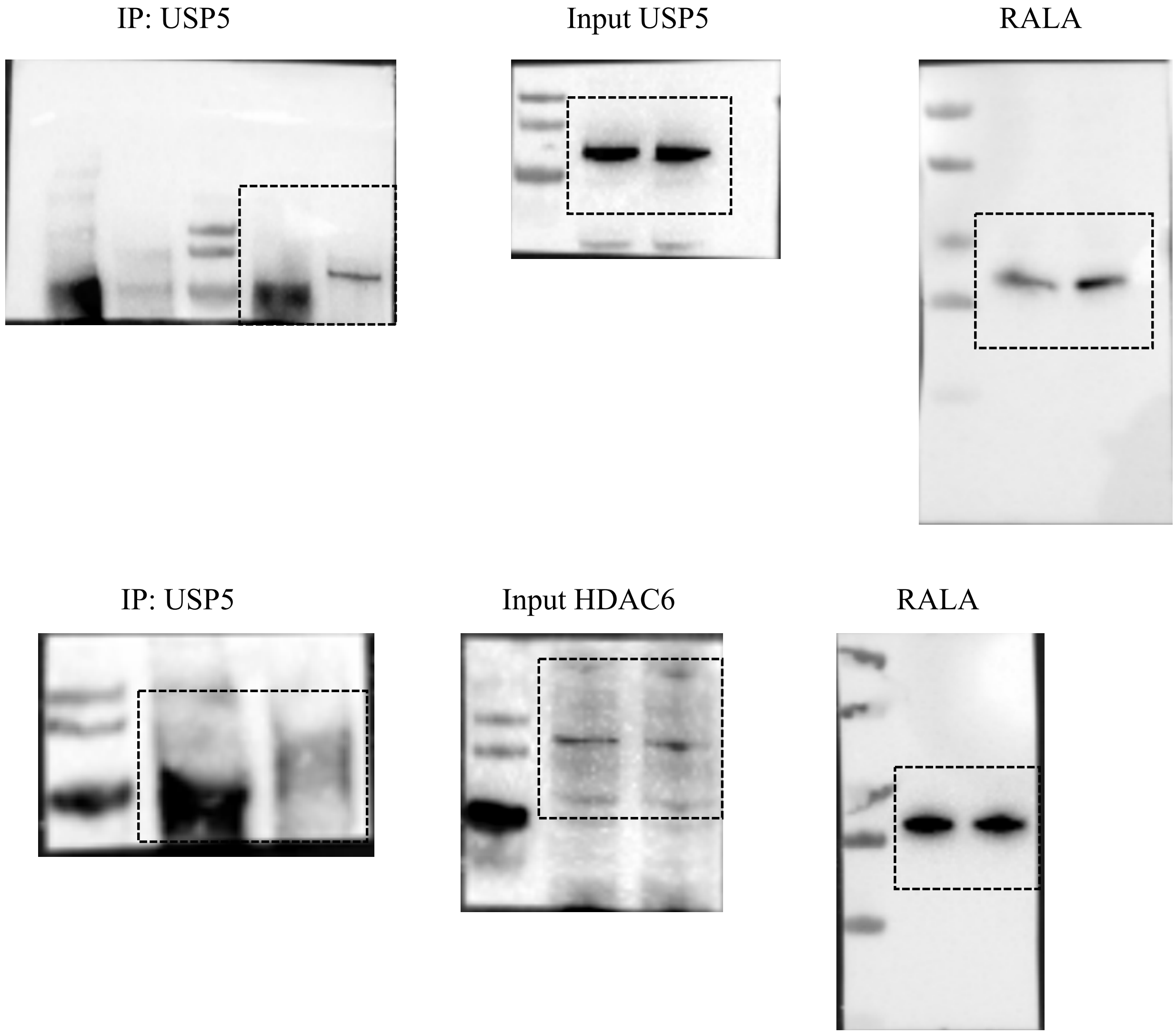

IP: USP5

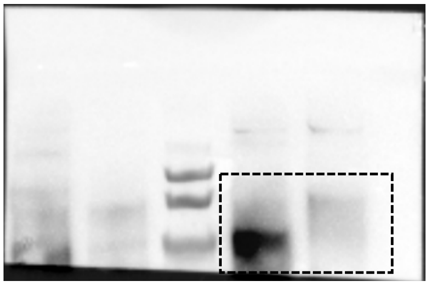

Input NBR1

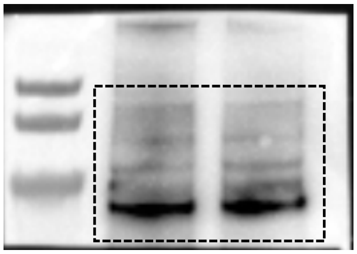

RALA

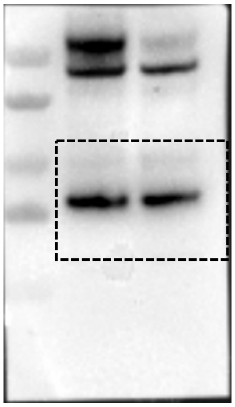

IP:USP5

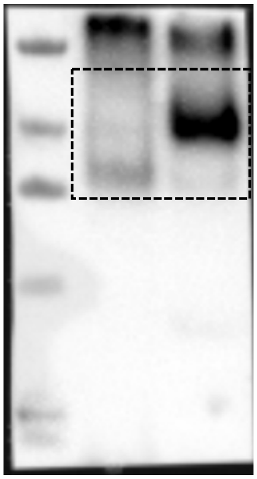

Input PSMC3

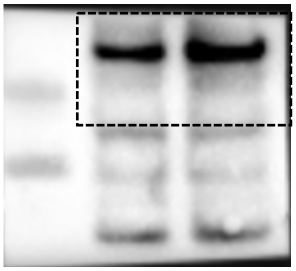

RALA

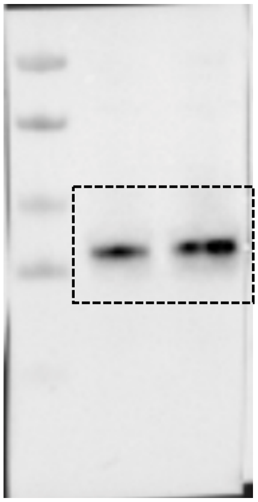

IP:USP5

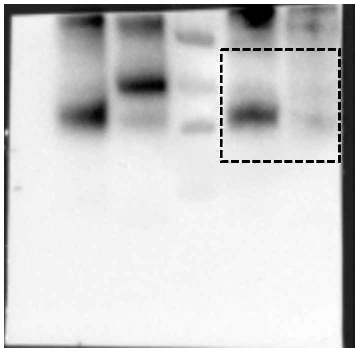

Input PSMA4

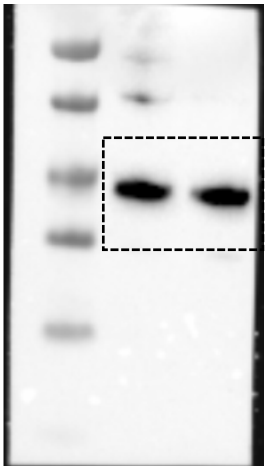

RALA

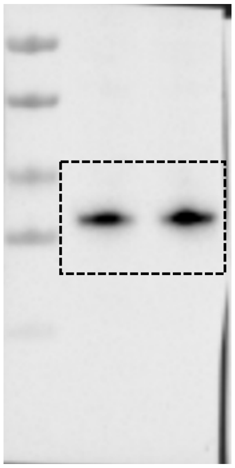

IP:USP5

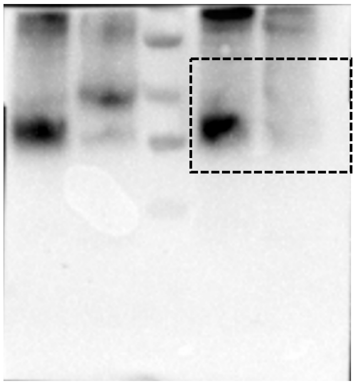

Input PSMC4

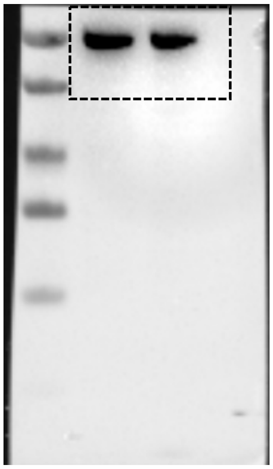

RALA

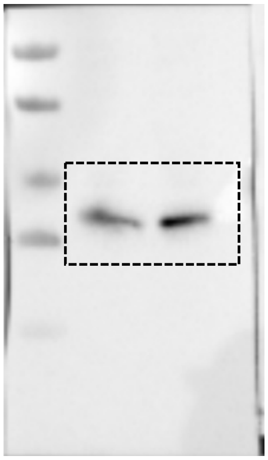

IP:USP5

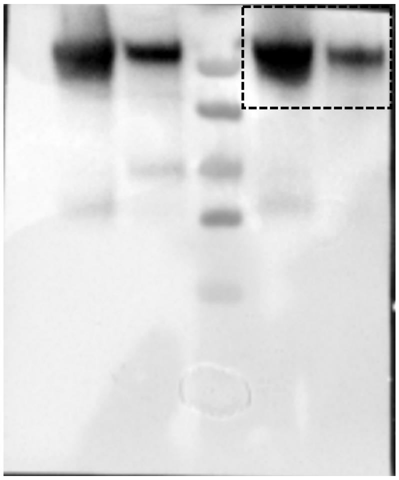

Input PSMD11

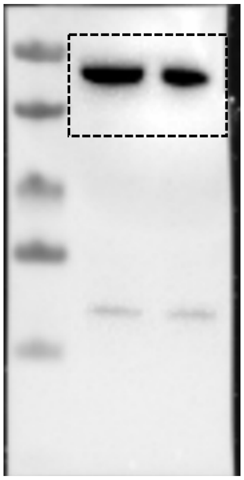

RALA

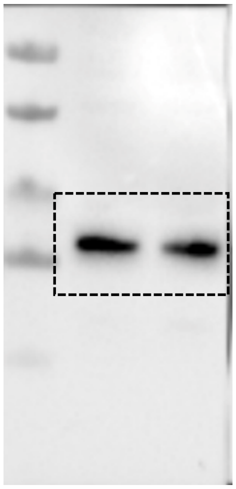

IP:USP5

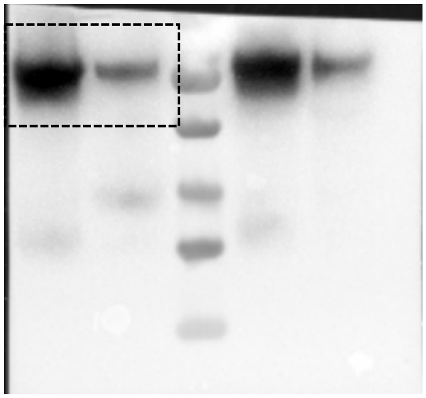

Input PSMD12

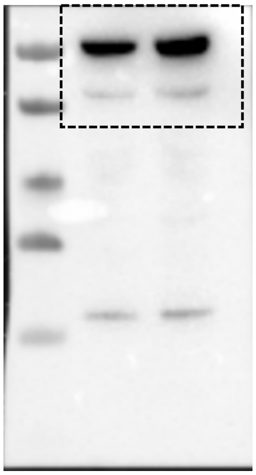

RALA

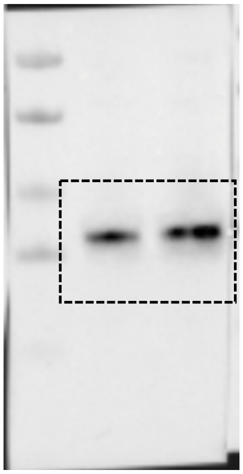

IP:USP5

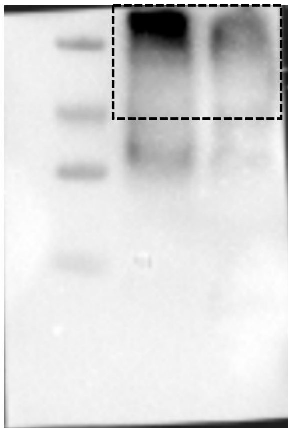

Input PSMD14

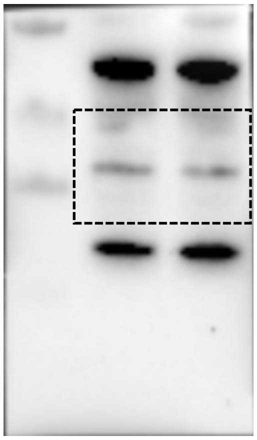

RALA

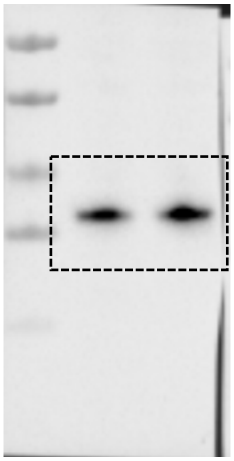

Figure 2B

USP5 GAPDH

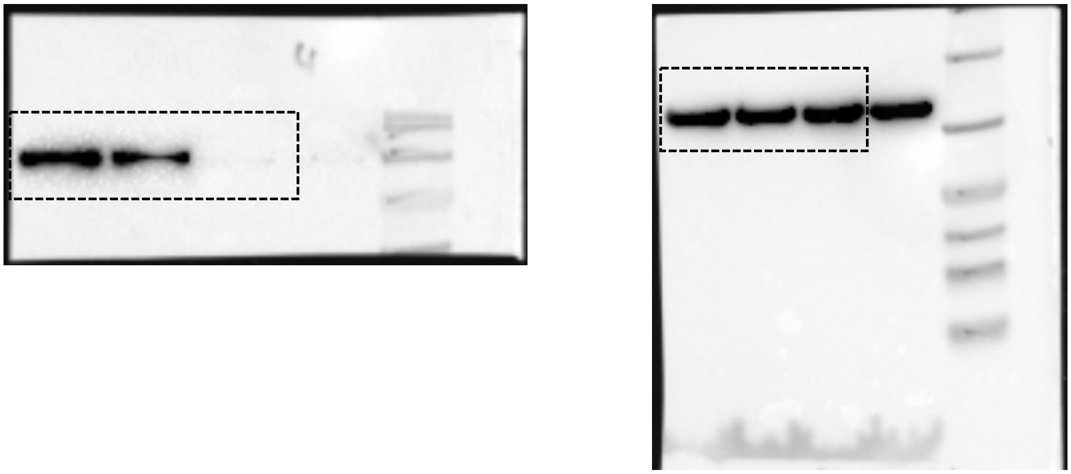

Figure 2E

Ubiquitin Ub3

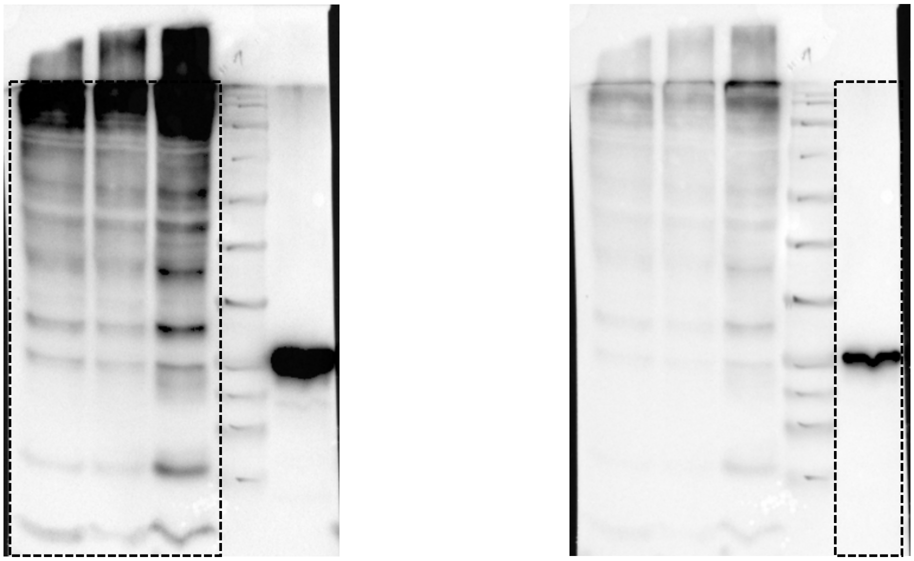

Figure 2G

K48-Ub K48 chains

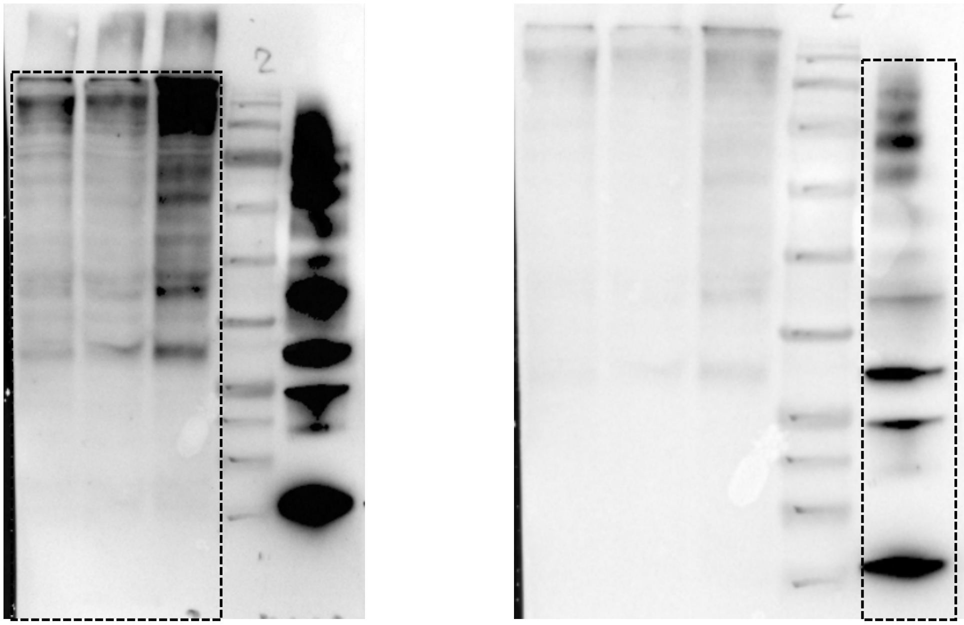

Figure 2I

Ubiquitin GAPDH Ubiquitin GAPDH

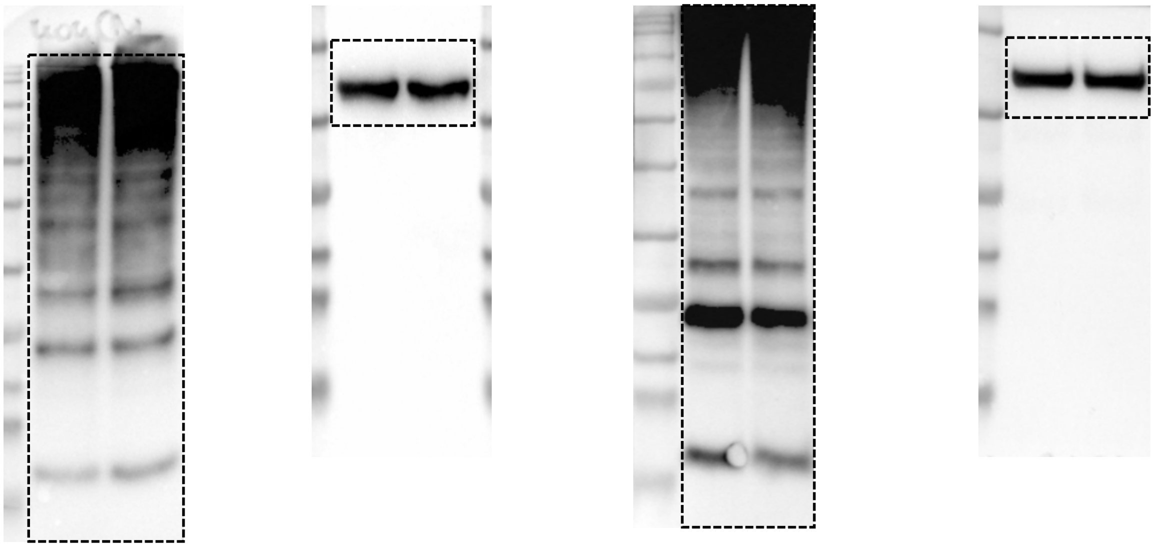

non-CMs MEFs

Ubiquitin GAPDH

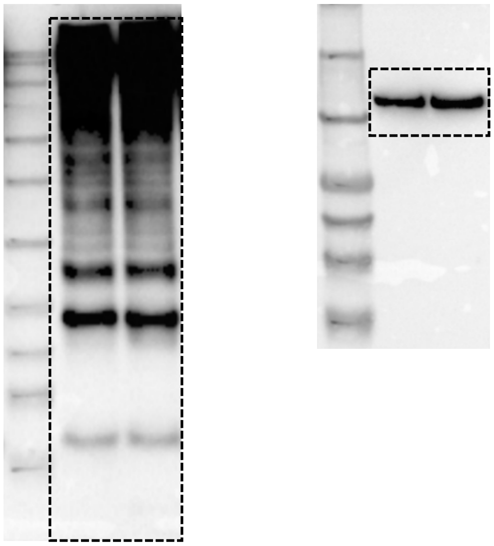

Hepatos

Figure 2K

USP5 GAPDH USP5 GAPDH

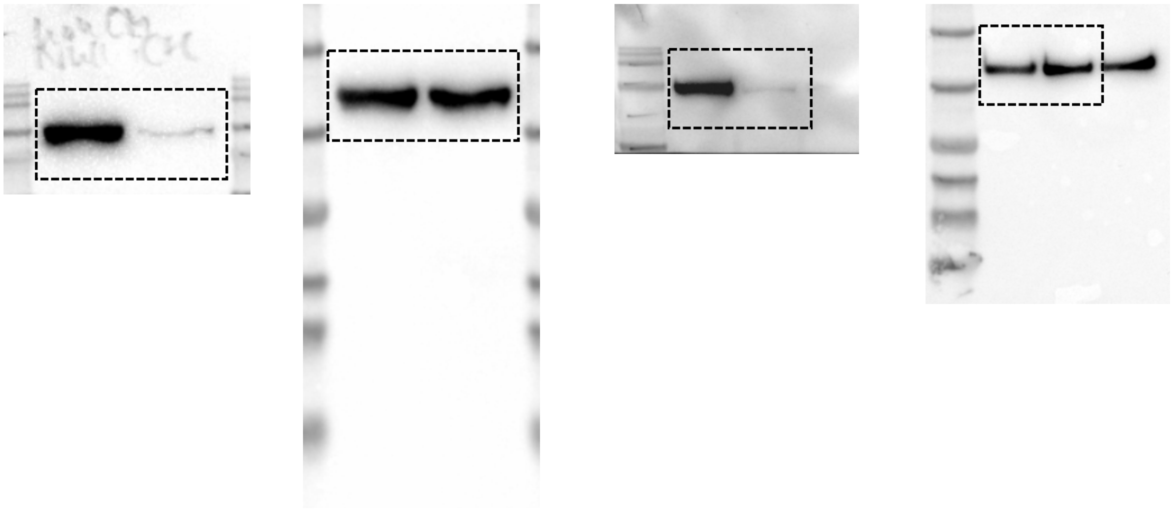

non-CMs MEFs

USP5 GAPDH

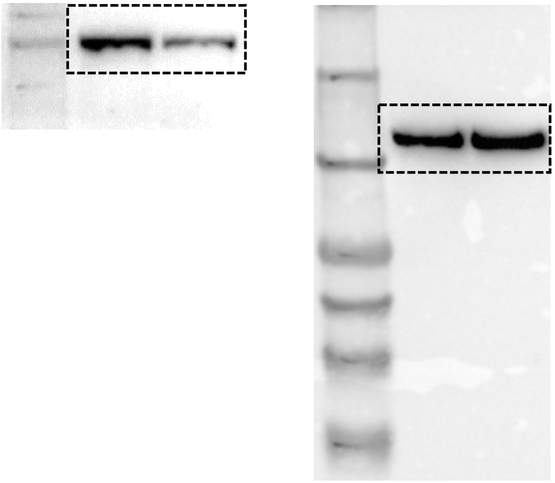

Hepatos

Figure 3B

Native Gel

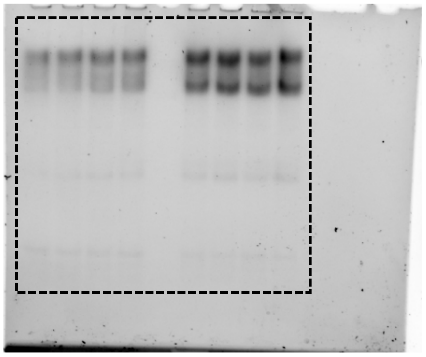

Figure 3E

Native Gel

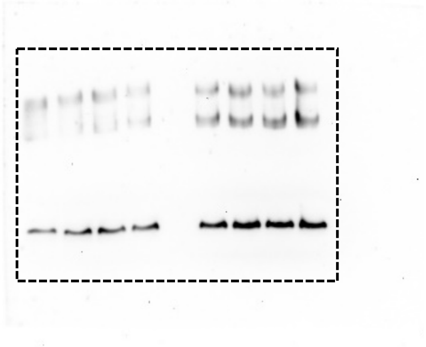

Figure 4A

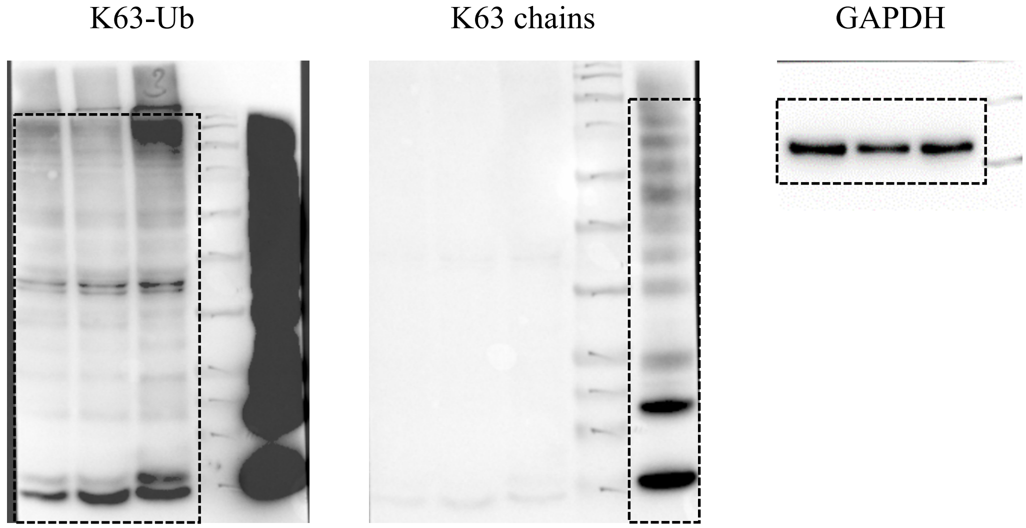

Figure 4C

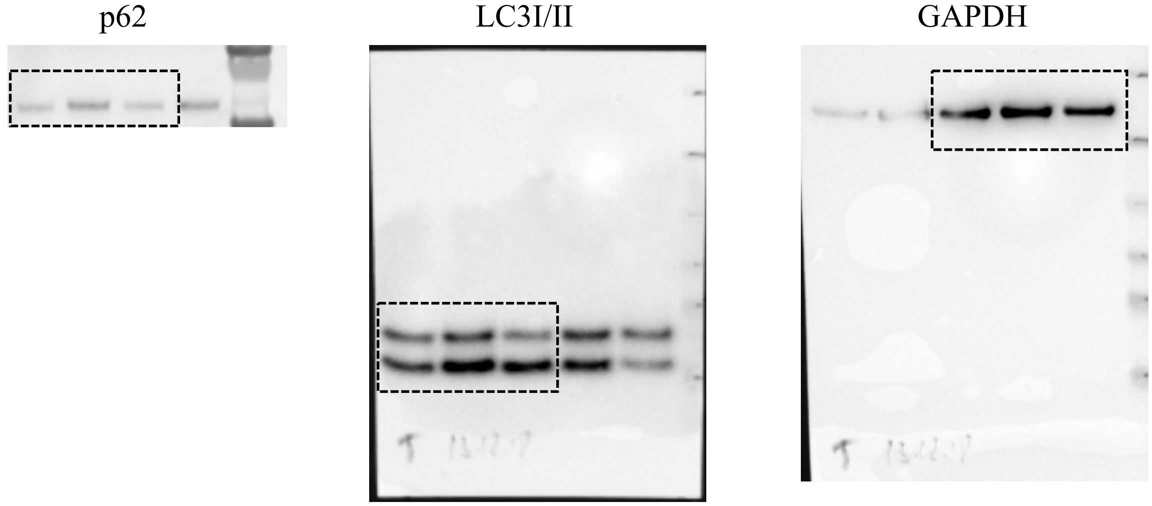

Figure 4F

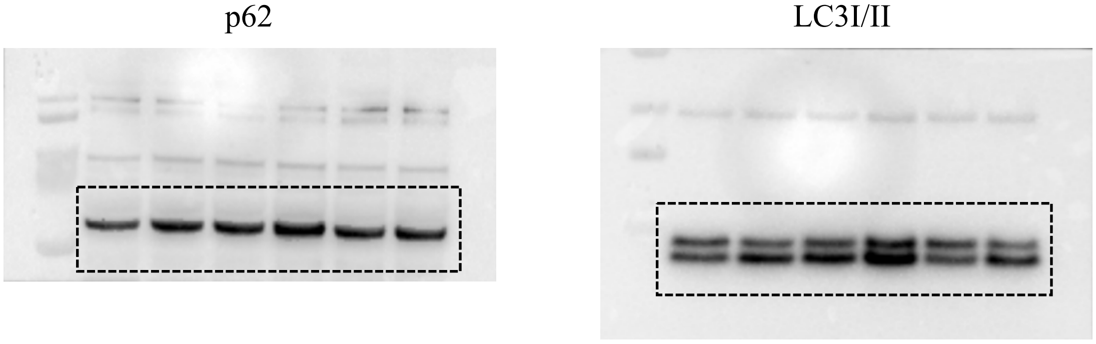

GAPDH

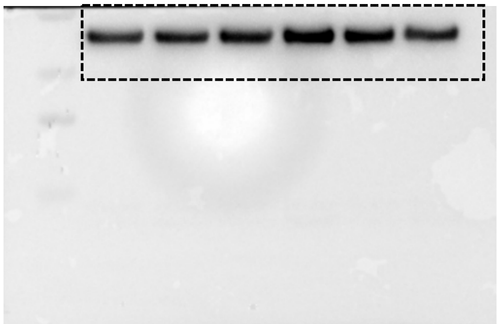

Figure 4J

p62

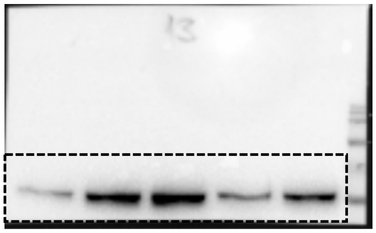

LC3I/II

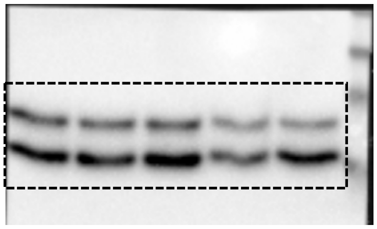

GAPDH

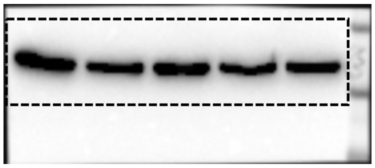

Figure 4K

(cleaved) Caspase 3

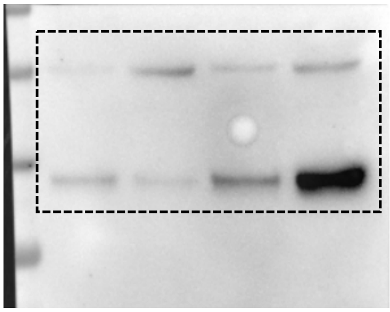

GAPDH

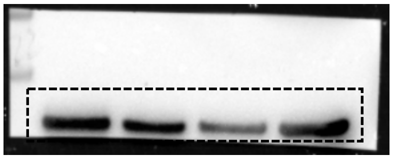

Figure 6E

Ubiquitin

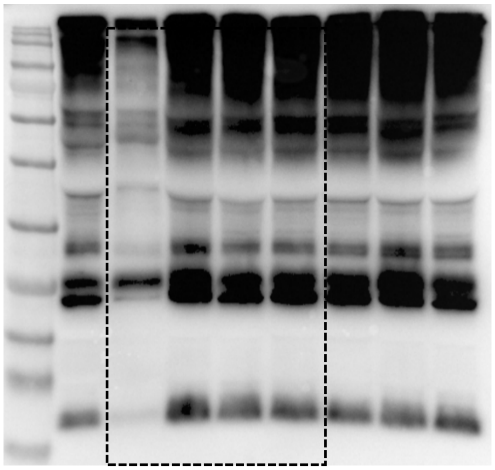

GAPDH

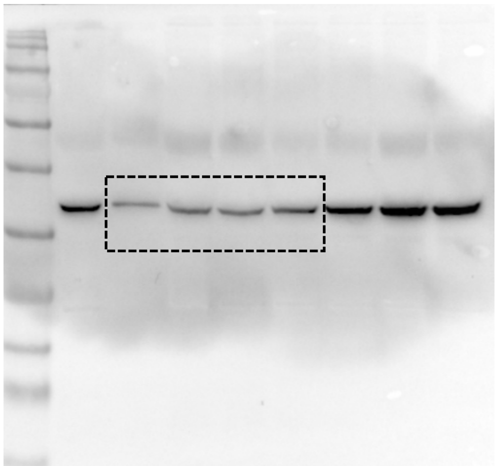

Figure 6I

USP5

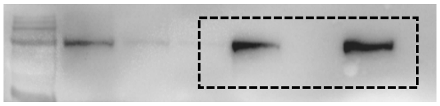

GAPDH

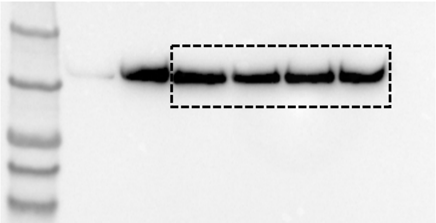

USP5

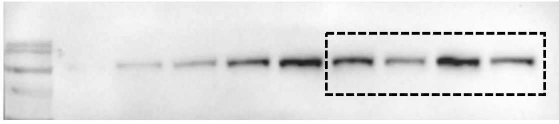

GAPDH

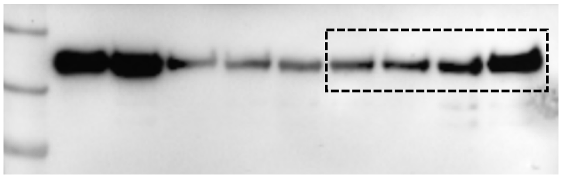

Figure 7A

FLAG

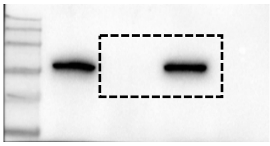

GAPDH

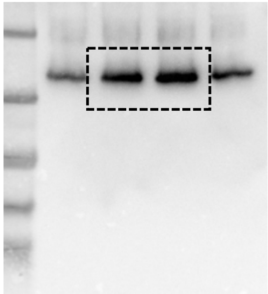

USP5

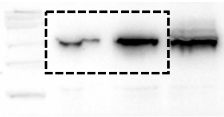

GAPDH

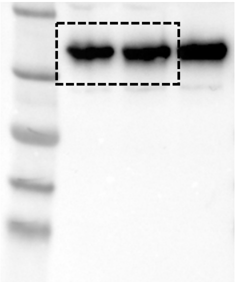

Figure 7C

Ubiquitin FLAG GAPDH

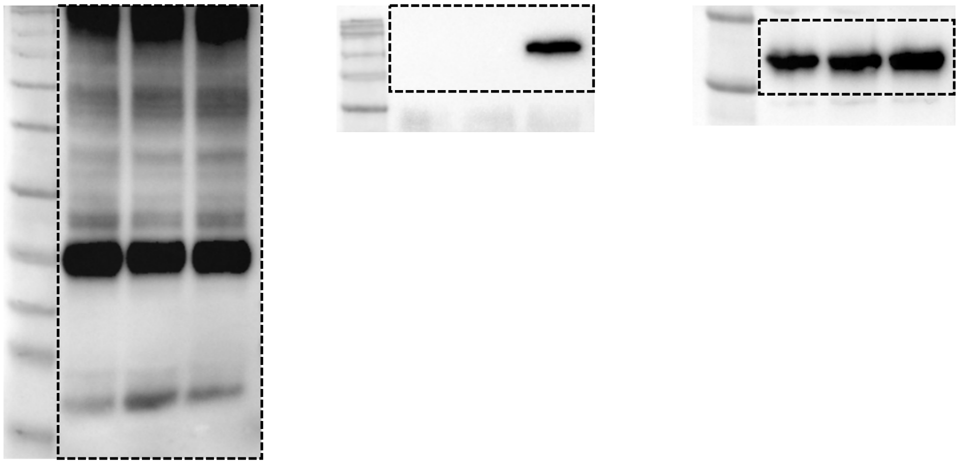

Figure 7L

Ubiquitin GAPDH

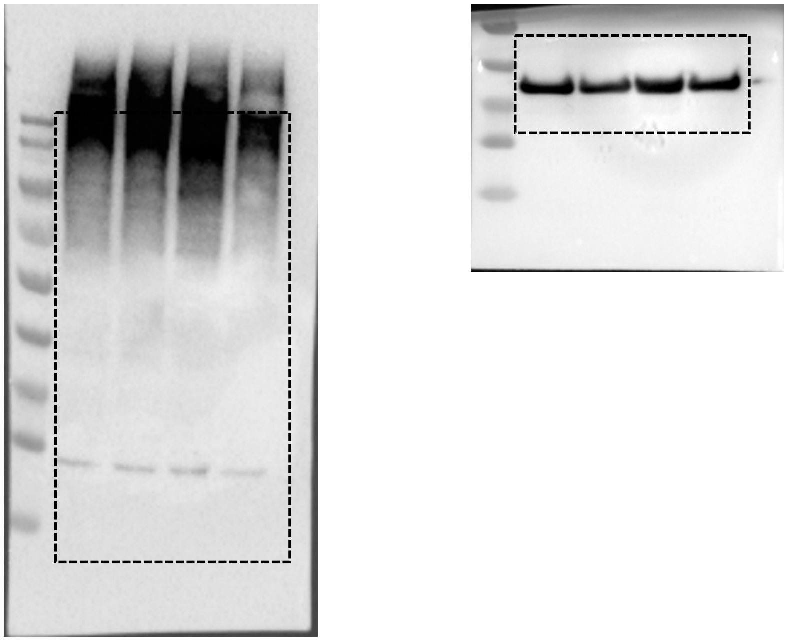

Figure 7N

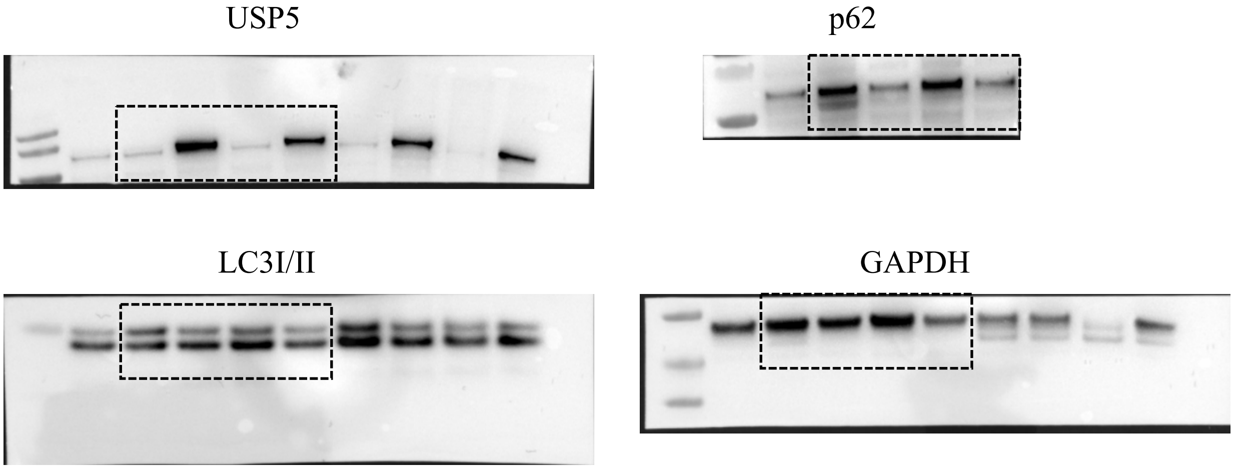

Figure 8B

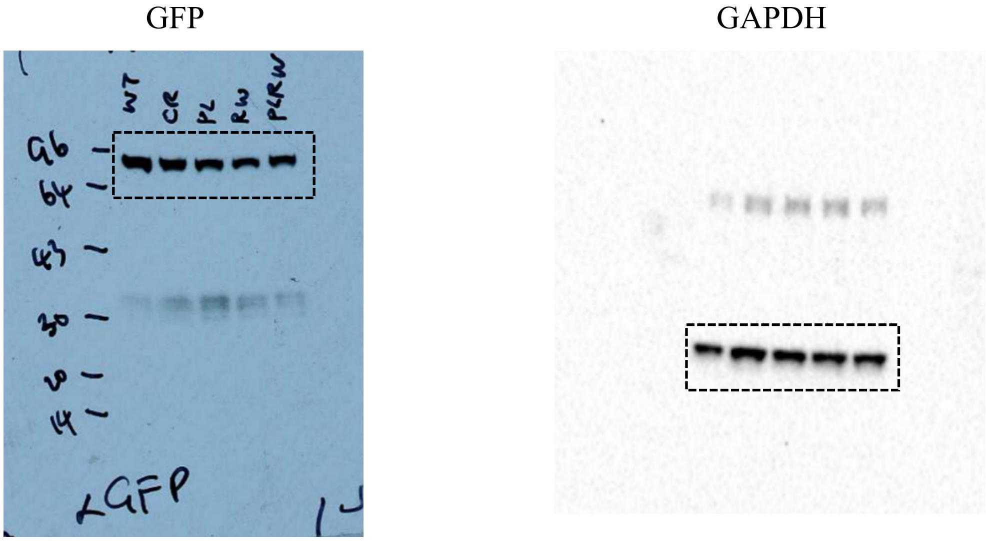

Figure 8E

GFP GAPDH

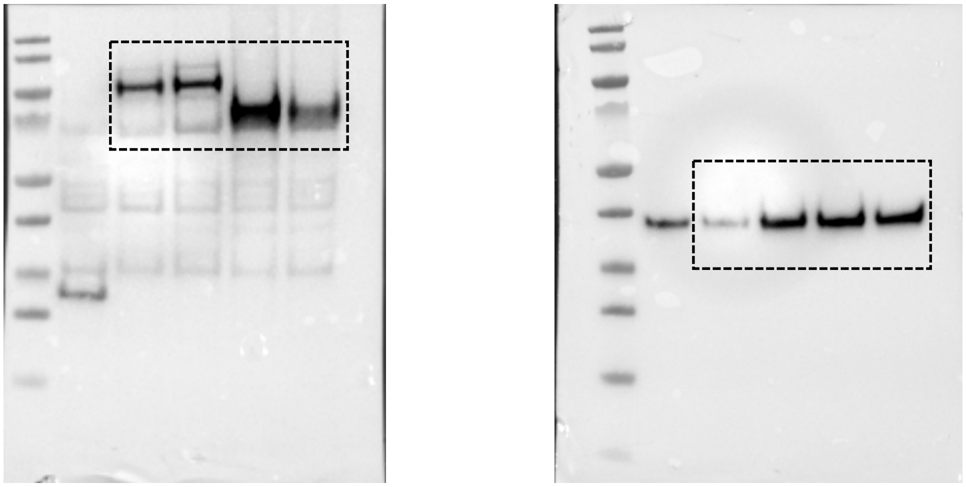

Figure 9A

USP5 GAPDH

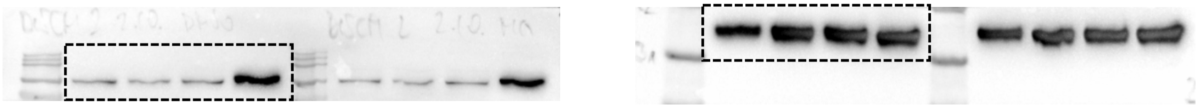

Figure 9C

Desmin GAPDH

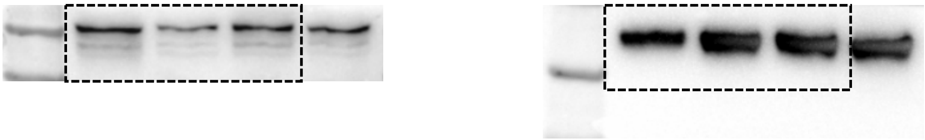

Figure 9G

Ubiquitin

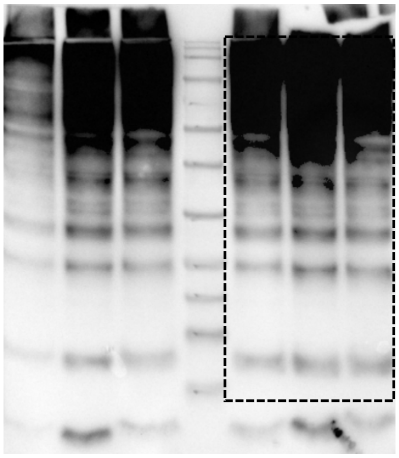

GAPDH

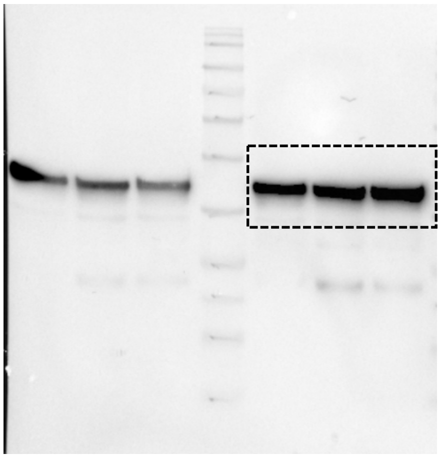

Figure 9J

p62

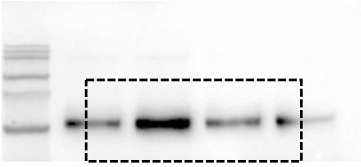

GAPDH

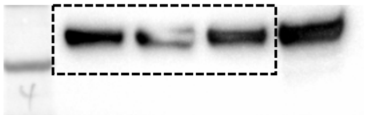

Figure 9L

LC3I/II

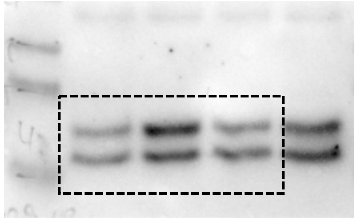

GAPDH

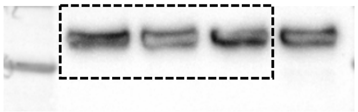

Figure S1A

IP:GFP

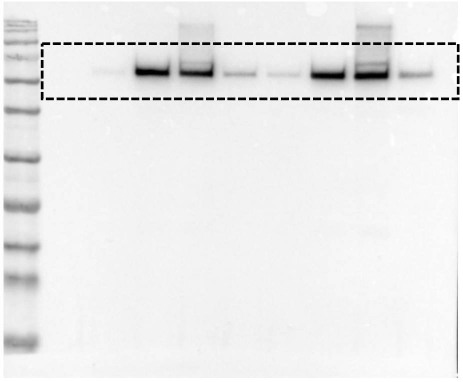

IP:V5

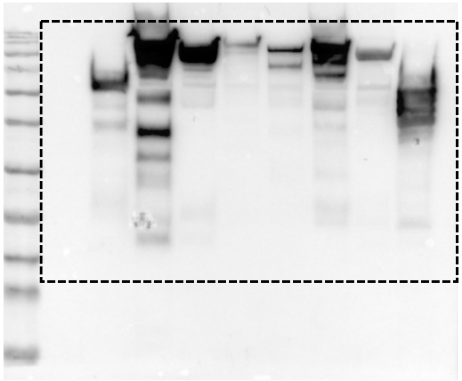

GFP

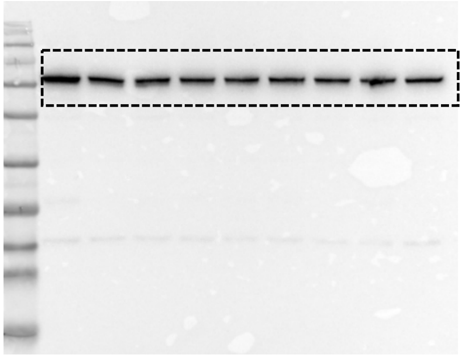

V5

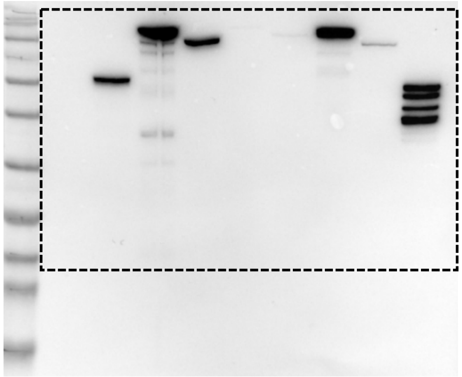

GAPDH

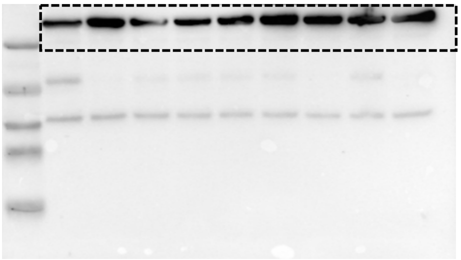

IP:GFP

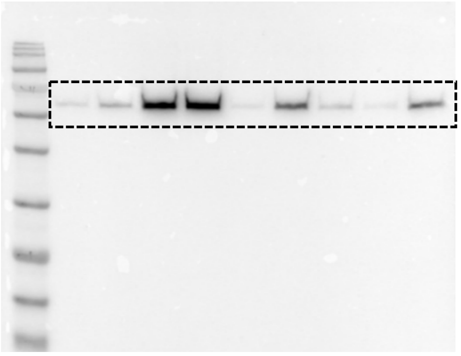

IP:V5

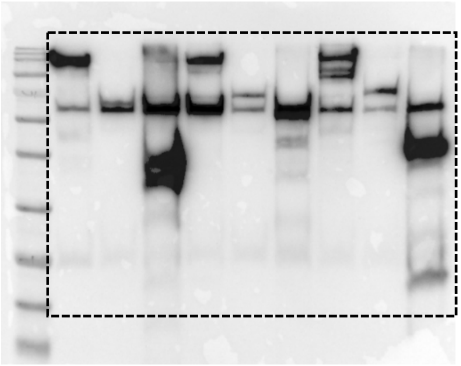

GFP

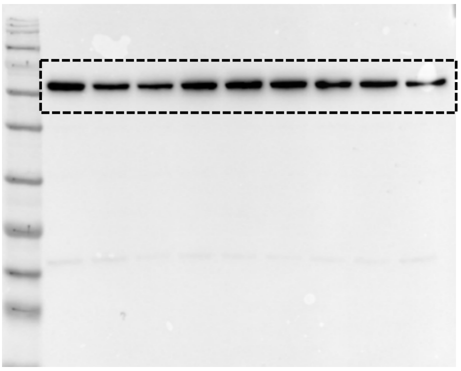

V5

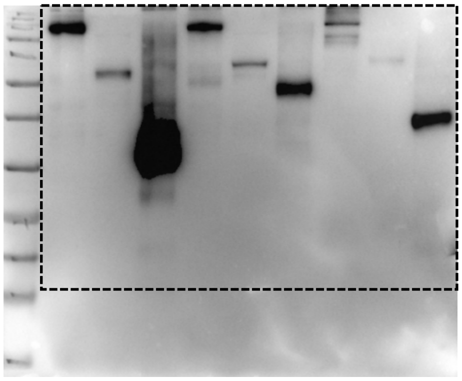

GAPDH

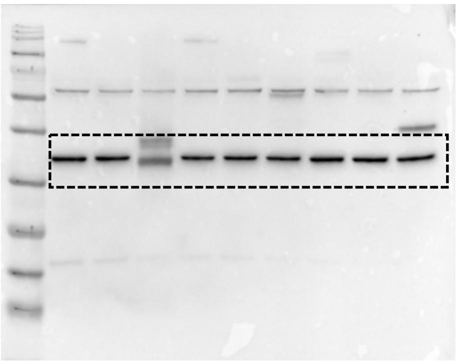

IP:GFP

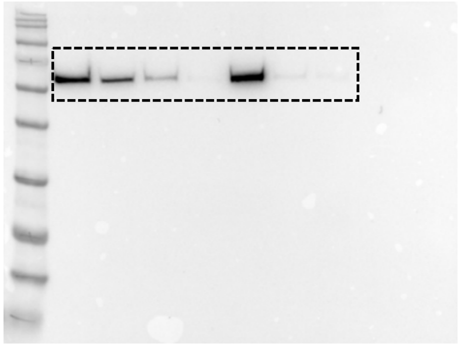

IP:V5

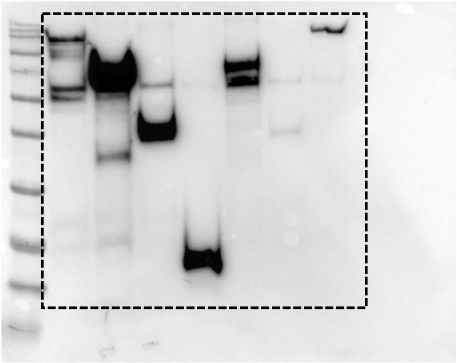

GFP

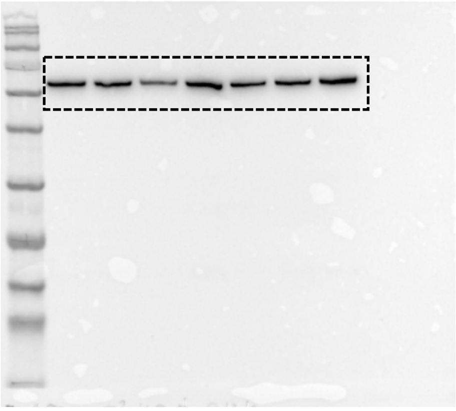

V5

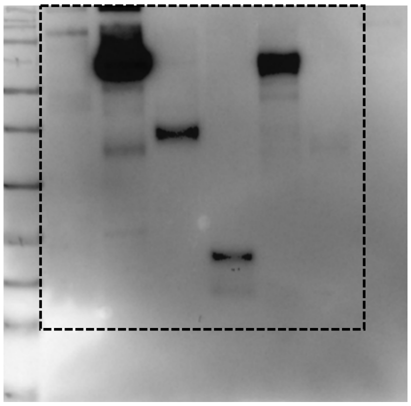

GAPDH

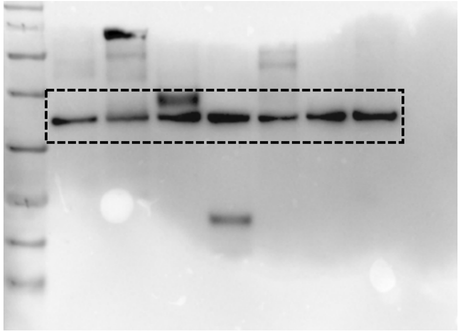

Figure S1B

IP:GFP

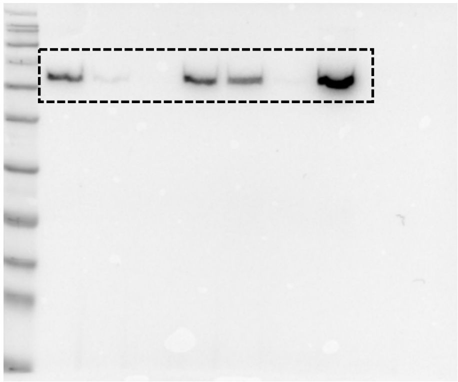

IP:V5

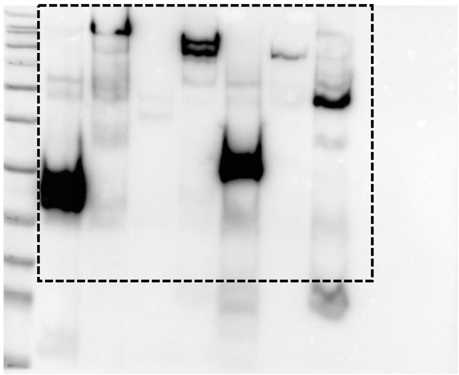

GFP

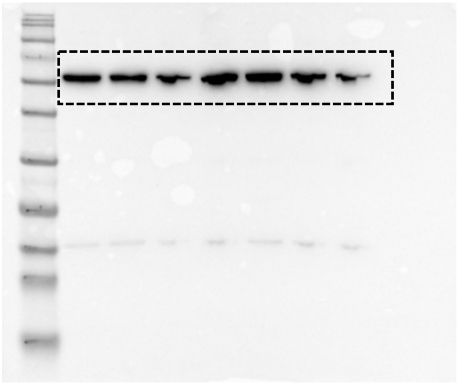

V5

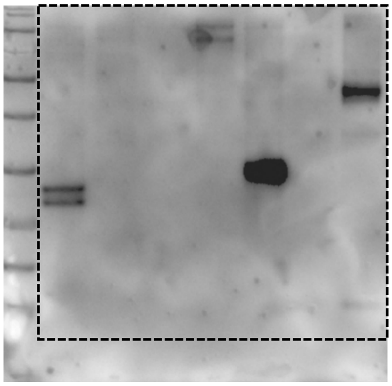

GAPDH

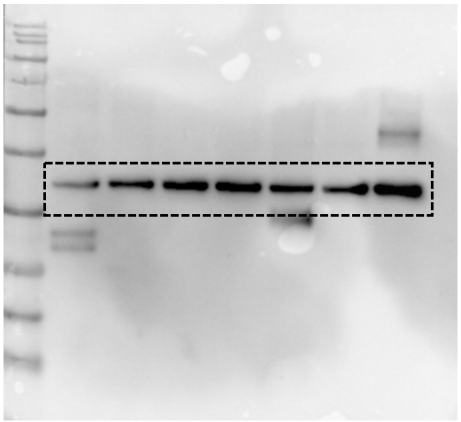

Figure S1C

IP:GFP

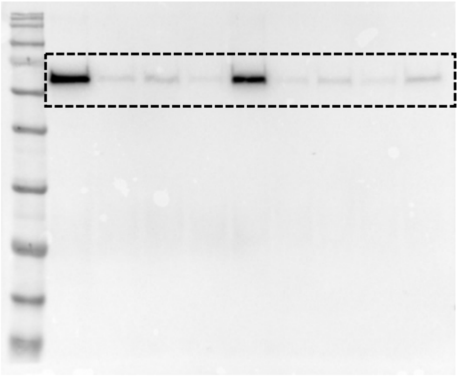

IP:V5

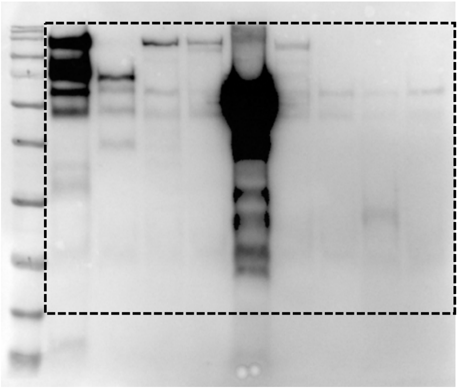

GFP

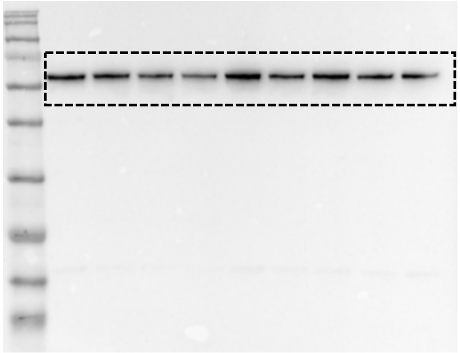

V5

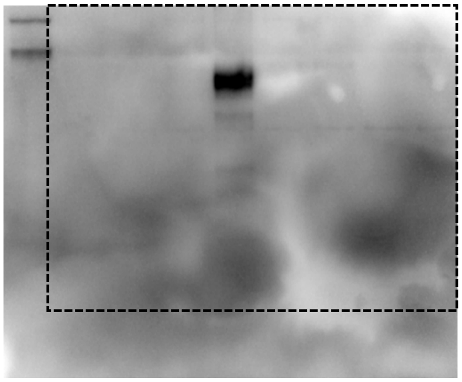

GAPDH

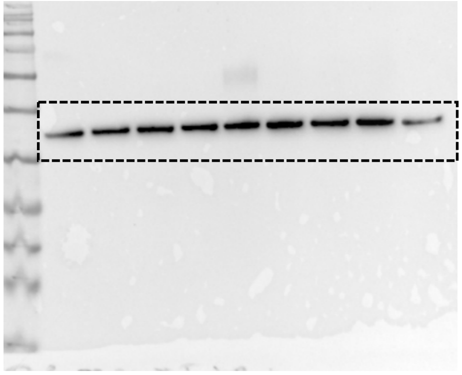

Figure S2G

IP:V5

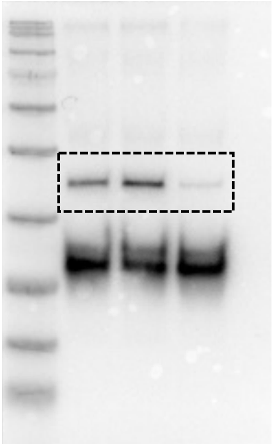

IP:GFP

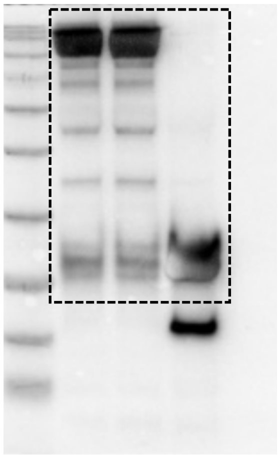

V5

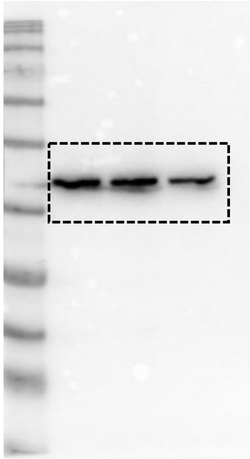

GFP

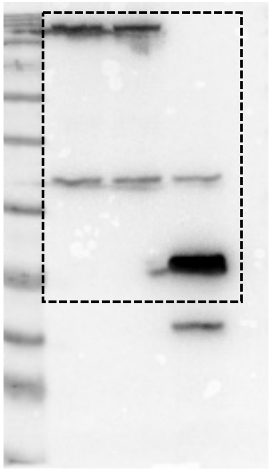

TUBULIN

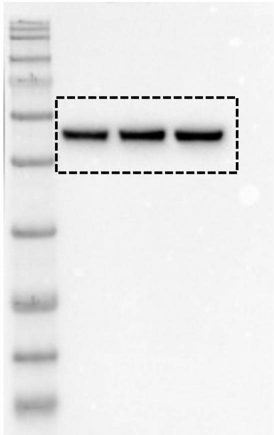

Figure S2H

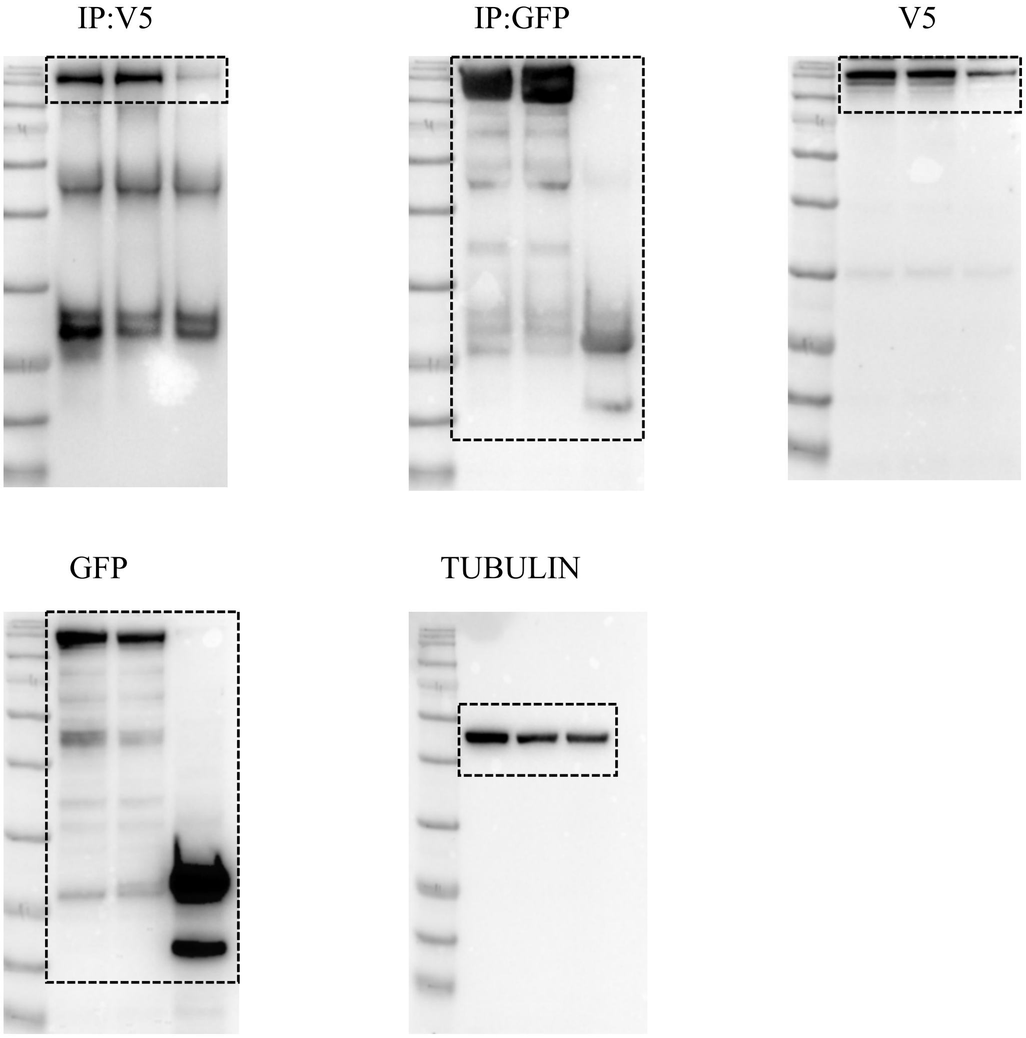

Figure S4E

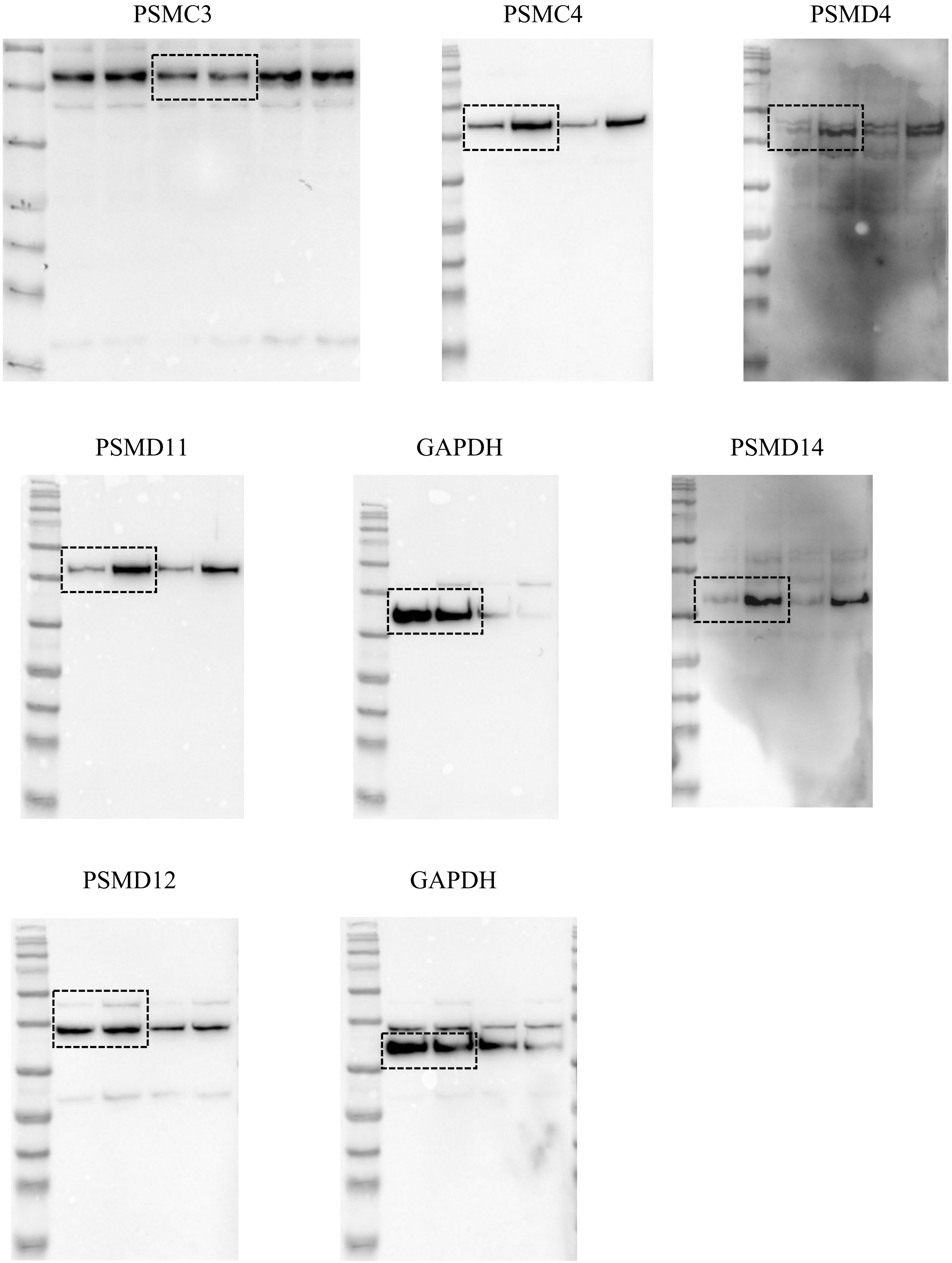

Figure S4G

PSMA4

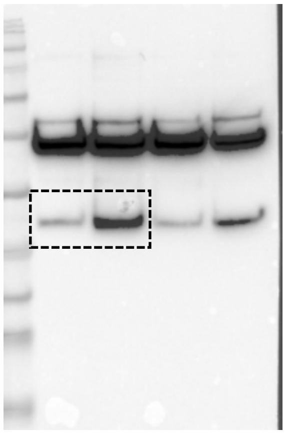

PSMA7

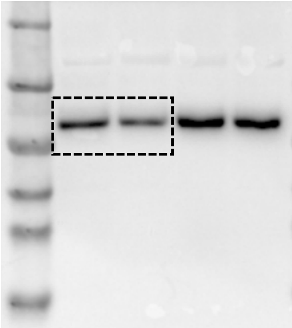

GAPDH

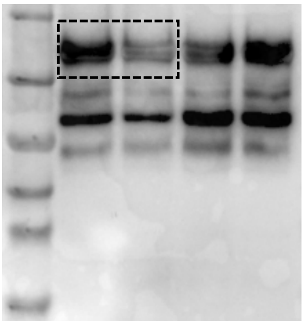

PSMB1

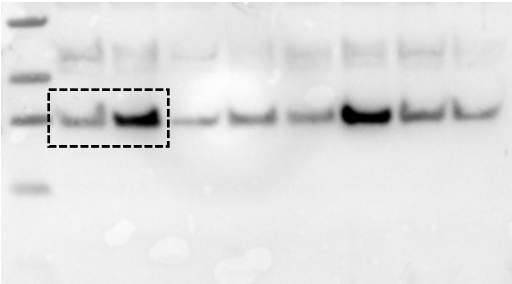

GAPDH

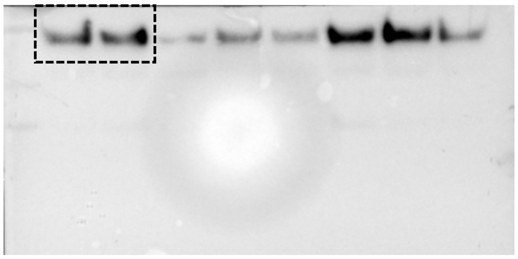

PSMB2

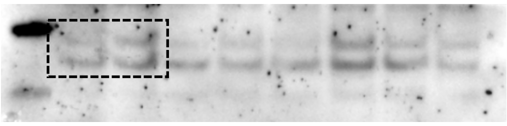

GAPDH

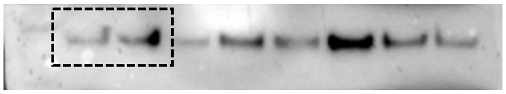

Figure S5C

USP5

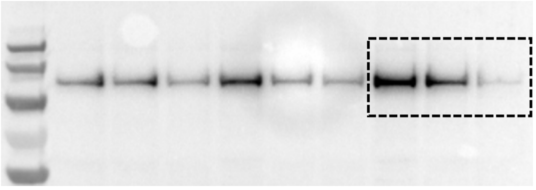

p62

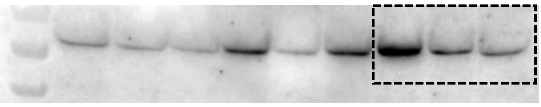

GAPDH

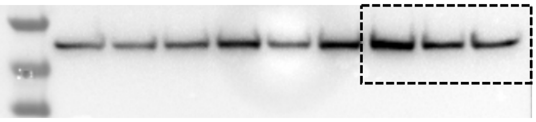

LC3I/II

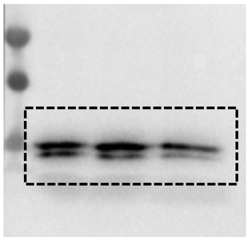

GAPDH

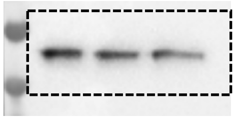

Figure S5G

Ubiquitin

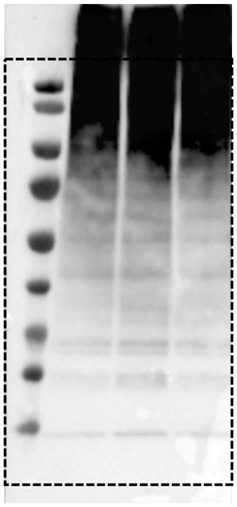

GAPDH

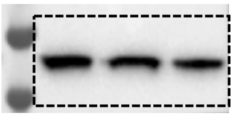

Supplement: Supplementary file 1 — Figs. S1 to S7 Legends for tables S1 to S3 Images of original uncropped Western blots [file sciadv.ado3852_sm.pdf]
